# Pharmacological assessment of drugs retained in the 1^st^ and 24^th^ WHO Essential Medicines List (1977–2025)

**DOI:** 10.1007/s00210-026-05555-8

**Published:** 2026-06-17

**Authors:** Lilly Josephine Bindel, Roland Seifert

**Affiliations:** Institute of Pharmacology, Hannover Medical School, 30625 Hannover, Germany

**Keywords:** WHO, Essential Medicines List, Essential medicines, Rational pharmacotherapy, Risk–benefit, Guideline recommendation, Drug efficacy, Drug safety, Drug selection

## Abstract

The Essential Medicines List (EML) provides international guidance for the selection of effective, safe, and essential drugs. Retained drugs were identified as medicines listed in both the 1^st^ and 24^th^ EML editions. The study aims to pharmacologically assess and to distinguish established therapeutic value from questionable or obsolete current relevance. Retained drugs were assessed by therapeutic value and indication-specific justification. Listed indications and the importance developments were compared between the 1^st^ (1977) and 24^th^ EML (2025). The justification of listing was assessed by guideline recommendations, efficacy and safety, scope of clinical usage, and availability of alternatives. A structured assessment algorithm was applied to assign drugs to therapeutic value categories. 129 retained drugs were assessed. Most EML indications did not change (*n* = 65), followed by specifications (*n* = 26), additions (*n* = 24), narrowing (*n* = 8), or change (*n* = 6). 71 drugs (55.0%) were classified as having constant importance, 20 (15.5%) as increasing, and 38 (29.5%) as declining. Drugs can be categorized in preserved and clearly justified value, niche value, requiring indication correction, and obsolescence. Nineteen drugs were identified where continued EML listing warrants discussion, mainly due to restricted or second-choice use, reduced efficacy, high pathogen resistance, and toxicity issues. Historical retention on the EML does not necessarily indicate unchanged therapeutic value. A structured pharmacological assessment can distinguish valuable from obsolete retained drugs. EML-listed drugs require regular reassessment to ensure that future EML editions remain clinically justified and relevant for public health.

**Supplementary Information:**

The online version contains supplementary material available at https://doi.org/10.1007/s00210-026-05555-8.

## Introduction

Every two years, the World Health Organization (WHO) publishes the “Model List of Essential Medicines”, which is also known as the “Essential Medicines List” (EML) (WHO 2025a). The first edition was introduced in 1977 (WHO 1977), while the most recent, the 24^th^ edition of the EML was published in 2025 (WHO 2025b). Initially, the EML aimed to “extend the accessibility of the most necessary drugs to those populations whose basic health needs could not be met by the existing system,” with the “main objective […] to extend the primary health care coverage of the population” (WHO 1977). The first EML was primarily developed through expert opinion and consensus (WHO 1977). Current key criteria for the selection of essential medicines include evidence of efficacy and safety, public health need, cost-effectiveness, and availability in appropriate quality and dosing (WHO 2025a), with the introduction of cost-effectiveness as a criterion for the selection since 2002 (WHO 2002). Applications for changes to the EML are submitted by external stakeholders and assessed every two years by an independent WHO Expert Committee, which reviews the available evidence and formulates recommendations on additions, deletions, or modifications (WHO 2025b). Current EML revisions therefore follow a more formalized evidence-based application and review process compared with the primarily consensus-based development of the first EML.

Over the years, the EML has developed into an important global guidance tool, serving as a reference for national EML development, drug supply priorities, choices for national health programs, and as an orientation for rational pharmacotherapy (Laing et al. 2003; Peacocke et al. 2022; WHO 2025a). Such guidance is particularly relevant in the context of substantial pharmacological innovation, expanding treatment options, and rising costs (Morgan et al. 2020; Ghinea 2024).

However, the increasing number of available and often highly specialized therapies also raises the question which medicines should be considered truly essential, and whether the large list of essential medicines still fulfil their role as essential therapeutics. Historical retention alone does not necessarily imply unchanged therapeutic importance. Some drugs may have remained relevant for the same indication, whereas others may have gained new indications, shifted to another usage field, become restricted to reserve or second-choice use, or lost importance due to toxicity, resistance, declining availability, or replacement by more favourable alternatives. This is particularly relevant for anti-infective drugs, where changes in pathogen resistance patterns, evolving treatment guidelines, and shifting epidemiology can lead to more frequent changes in efficacy and recommended use.

Retained drugs are defined as medicines listed in both the 1^st^ (1977) and 24^th^ (2025) EML edition and therefore retained for nearly five decades. Therefore, a reassessment of long-standing EML-listed drugs may provide further insight into how the concept of essential medicines has evolved over time and whether retained drugs still reflect current standards of rational and evidence-based pharmacotherapy. Such analysis may also help to identify drugs or indications requiring reconsideration, correction, or further discussion in future EML revisions.

The present study focuses on a pharmacologically orientated assessment of the retained drugs. For this purpose, listed indications, guideline recommendations, evidence of efficacy and safety, scope of clinical usage, public health need, and availability of more favourable alternatives were evaluated. Three related but distinct levels of assessment were applied. First, the development of listed indications was assessed by comparing the EML 1977 with the EML 2025. Second, the trend in therapeutic importance was classified as constant, increasing, or declining. Third, the justification of continued EML listing was evaluated and integrated into therapeutic value categories, distinguishing drugs that remain clearly valuable from drugs with low value or obsolescence. The study contributes to the understanding of the long-term development of the EML. Thereby, future EML revisions are supported with regard to rational, evidence-based, and clinically justified essential pharmacotherapy.

## Methods

### Setting and data

The 1^st^ (1977) and 24^th^ (2025) editions of the WHO Essential Medicines List (EML) were compared (WHO 2025b, 1977) to identify retained drugs. These are defined as drugs that were listed in both the 1^st^ EML in 1977 and the 24^th^ EML in 2025, thereby representing medicines with long-standing retention on the WHO EML.

The analysis is based on WHO technical reports and corresponding EML lists, with particular focus on the listed indications in the 1^st^ and 24^th^ editions (WHO 2026). Literature sources for the assessment of retained drugs included current guidelines, reviews, drug information sources, and selected clinical literature addressing efficacy, safety, scope of clinical usage, and public health relevance. The sources were gathered through a primary literature search in PubMed and consultation of governmental and international health organization websites. If available, current guidelines or several publications with a high level of evidence were considered. The literature search and drug assessment were performed between the 20^th^ November and the 8^th^ December 2025 by L.J.B., while both authors reviewed and verified the summarized evidence and final drug assessments.

**Fig. 1:**
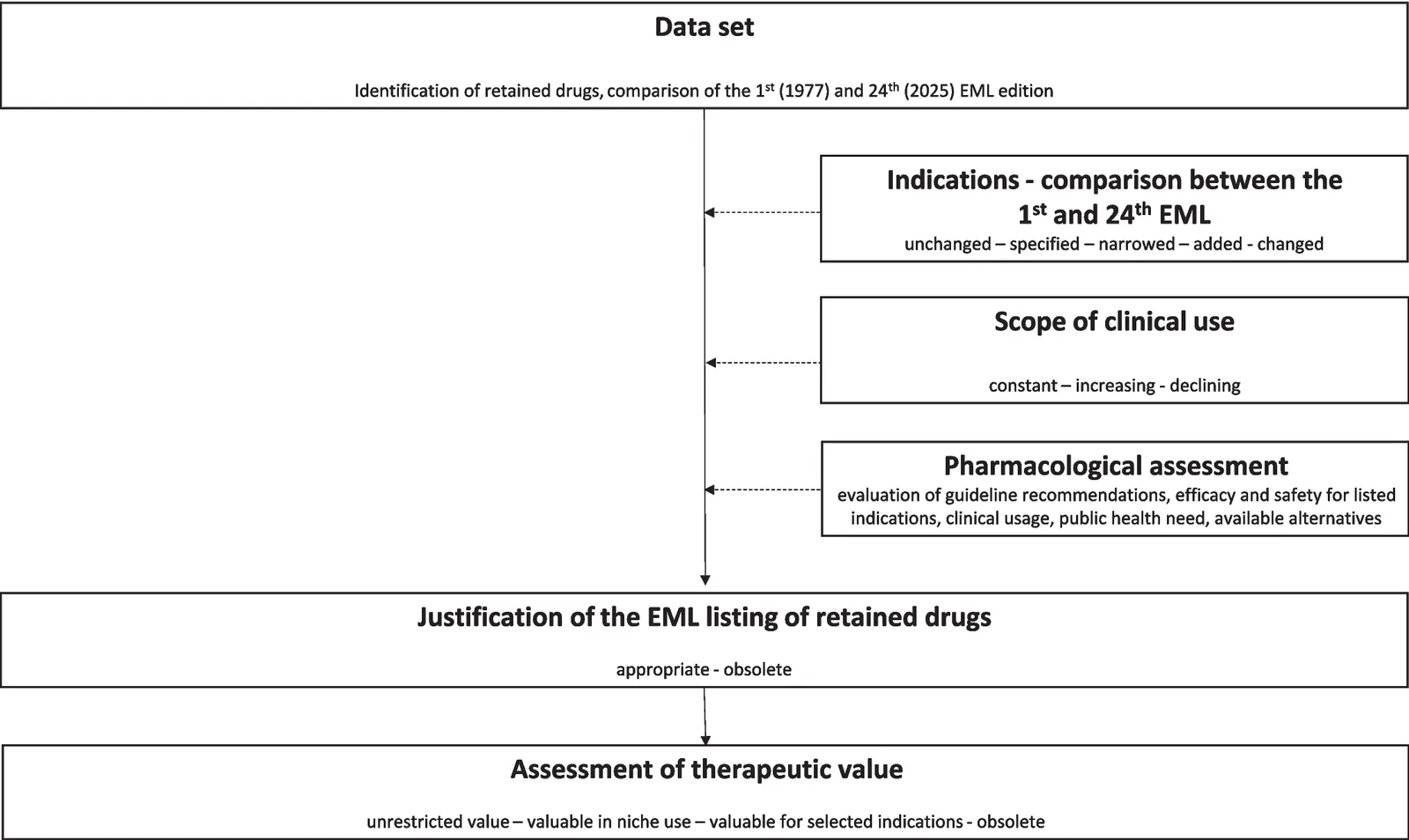
Methodological flowchart and overview about considered aspects for the assessment of retained drugs

Data extraction and classification were performed manually, and Excel was used for data organization. GraphPad Prism 11 and Microsoft Power Point were used for data visualization. In Fig. 1, a flowchart about the methodological procedure is provided, while Table 1 summarizes and explains the applied criteria for the drug assessment. The supplemental references correspond to the literature considered in the comprehensive assessment of the retained drugs presented in the supplemental material. These references were considered essential for an assessment that is as objective and verifiable as possible.

**Table 1:** Explanation of applied criteria and the characterisation of retained drugs in regard of the assessment of indication development, scope of clinical use, pharmacological assessment, and its justification of EML listing. For each overarching aspect, the included criteria and characterization options are provided, along with an explanation

Aspect	Criterion	Characterization	Explanation
**Indication development**		unchanged indication	same indication in EML 1977 and EML 2025
		specified indication	indication defined more precisely in EML 2025
		narrowed indication	restriction to fewer or more specific indications
		added indication	expansion by additional indications
		changed indication	replacement by substantially different indication
**Development of the scope in clinical use**		constant use	maintained therapeutic relevance and established use
		increasing use	growing therapeutic importance or expanded use
		declining use	reduced therapeutic importance or replacement by alternatives
**Pharmacological assessment**	Recommendation in guidelines	first-line	preferred standard treatment
		second-line	recommended after failure or unsuitability of first-line therapy
		alternative	acceptable substitute treatment
		reserve	restricted use for selected or refractory cases
		not recommended	no current recommendation in guidelines
	Effectiveness and safety	effective	proven clinical efficacy with acceptable safety
		limited efficacy	reduced or insufficient therapeutic effect
		resistance	reduced effectiveness due to microbial resistance
		toxicity	clinically relevant harmful effects
		narrow therapeutic index	small margin between effective and toxic dose
		adverse effects	clinically relevant tolerability or safety concerns
	Public health need	high disease burden	relevance due to prevalence or mortality
		program relevance	importance in public health or control programs
		LMIC importance	relevance in low- and middle-income countries
		availability	importance due to accessibility or supply
		cost-effectiveness	therapeutic value in relation to costs
**Justification of EML listing**		retained	continued EML listing considered justified
		consideration for deletion	continued EML listing considered questionable

### Assessment of retained drugs

The listed indication in the 1^st^ EML was compared with the listed indication in the 24^th^ EML for each retained drug. Indications were extracted from explicit descriptions or deduced from the EML category in which the drug was listed. The development of indications was categorized as unchanged indication, specified indication, narrowed indication, added indication, or changed indication.

Drugs were categorized as having constant, increasing, or declining scope in clinical use. Constant usage was assigned when a drug remained established for its listed indication. Increasing use was assigned when the drug gained relevance through broadened indications, pharmacological versatility, or increasing disease burden. Declining use was assigned when the drug showed restricted or second-choice use, reduced efficacy, resistance problems, relevant toxicity, declining availability, or replacement by more favourable alternatives. Declining use was not considered equivalent to obsolescence, because some drugs with declining importance may still have relevant niche, reserve, endemic, or low-resource use.

Finally, the current therapeutic value and indication-specific justification of the EML-listing for each retained drug were assessed. Drugs were considered retained when the listing appeared justified. Drugs were considered for deletion when the current benefit–risk profile, guideline position, scope in clinical usage, or availability of more favourable alternatives made continued listing questionable. For some drugs, the listing itself was not considered obsolete, but specific indications were identified as requiring correction. Broader therapeutic value patterns were derived from the combined assessment of indication development, evidence of efficacy and safety, and development of importance.

## Results

### Development of listed indications of retained drugs

Although all 129 retained drugs were listed in both the 1^st^ and 24^th^ editions of the EML, their indications were not necessarily unchanged. When comparing the EML 1977 with the EML 2025, the indication development could be categorized as unchanged, specified, narrowed, expanded, or changed to another indication or usage field (Table 2). A detailed overview of the indication development and all other assessed aspects for retained drugs is provided in Table S1.

**Table 2:** Overview about the absolute number of retained drugs and their relative distribution across the examined aspects. This includes changes in EML-listed indications, development of the scope in clinical use, and appropriateness of EML listing. In Fig. 2–4, a visualization of the statistics is provided

Aspect	Characterisation	Number of retained drugs	Share of retained drugs
EML-listed indication	no change	65	50.4%
	specification	26	20.2%
	narrowing	8	6.2%
	change	6	4.7%
	addition	24	18.6%
Development of the scope in clinical use	constant	71	55.0%
	increasing	20	15.5%
	declining	38	29.5%
Appropriateness of EML listing	justified	110	85.3%
	questionable	19	14.7%

**Fig. 2:**
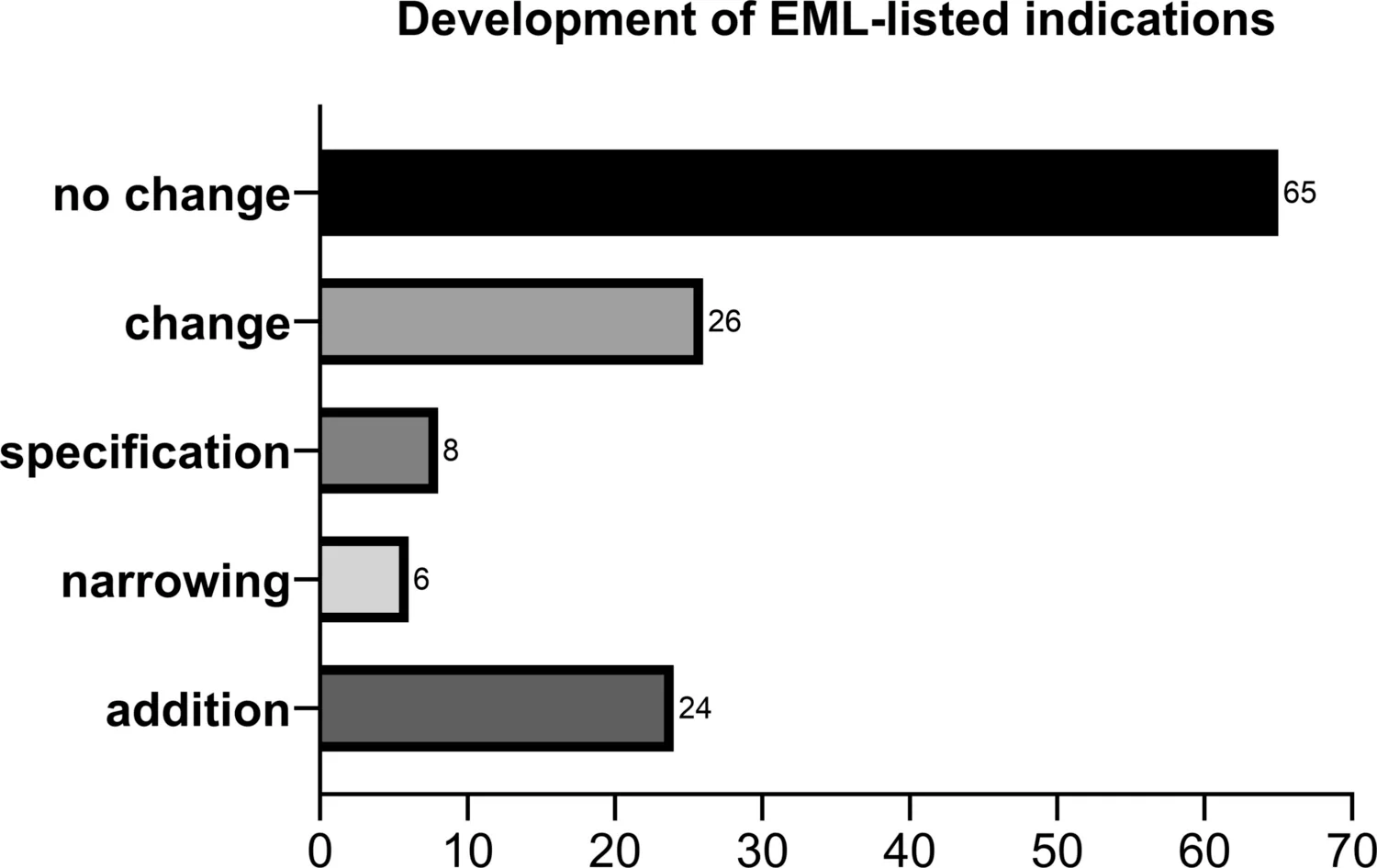
Overview about the indications of retained drugs for which they are considered essential, in comparison of the 1^st^ (1977) and 24^th^ (2025) edition of the EML (corresponding to Table S1). Drugs are categorized whether their indication changed, changed not, was specified, narrowed, or whether indications were added. For each category, the number of retained drugs are provided

The development of listed indications can be distinguished into unchanged indications, specified indications, narrowed indications, added indications, and changed indications or usage fields. Most retained drugs showed no relevant change in their listed indication between 1977 and 2025 (*n* = 65, 50.4%) (Fig. 2). Examples include folic acid for anaemia, rabies vaccine for immunization, ethambutol for tuberculosis, morphine for pain, oral rehydration salts for diarrhoea and rehydration, and potassium chloride for correction of electrolyte disturbances.

A specification of the indication without a major change in the general usage field was observed for 26 drugs (20.2%). Examples include amitriptyline, which is now listed for depressive disorders, doxycycline, which is now assigned to defined infectious indications, and doxorubicin, which is now listed for several specified malignant diseases. Similar specifications were observed for several anti-infective and antineoplastic drugs, including cloxacillin, gentamicin, cyclophosphamide, fluorouracil, vincristine, and methotrexate.

A narrowed or restricted indication (*n* = 8, 6.2%) was observed particularly among anti-infective drugs. Examples include ampicillin, now listed as a second-choice option for acute bacterial meningitis, chloramphenicol, now also restricted to a second-choice role for acute bacterial meningitis, procaine benzylpenicillin, now listed as a second-choice option for syphilis, and tetracycline, now listed for ophthalmological infections. Further examples of narrowed or specified use include amodiaquine, erythromycin, streptomycin, quinine, sulfadiazine, and pyrimethamine.

Some drugs experienced a major change of indication (*n* = 6, 4.7%). Isosorbide dinitrate was initially listed for hypertension and is now listed for angina. For example, diazoxide was initially listed for hypertension and is now listed for hypoglycaemia, propranolol changed from hypertension to migraine prophylaxis, and ephedrine changed from asthma to use in local anaesthesia-related settings.

The addition of indications was observed for 24 drugs (18.6%). Amphotericin B was initially listed for fungal infections and is now additionally listed for leishmaniasis. Epinephrine was initially listed for asthma and is now additionally listed for COPD, local anaesthesia, allergic reactions, arrhythmias, and mydriatic use. Acetylsalicylic acid gained additional indications in acute migraine attack, cardiovascular prevention, anticoagulant-related use, and juvenile joint disease. Further examples with added indications include methotrexate, dexamethasone, hydrocortisone, prednisolone, furosemide, spironolactone, calcium gluconate, potassium iodide, and salbutamol.

### Trends in the scope of clinical use of retained drugs

**Fig. 3:**
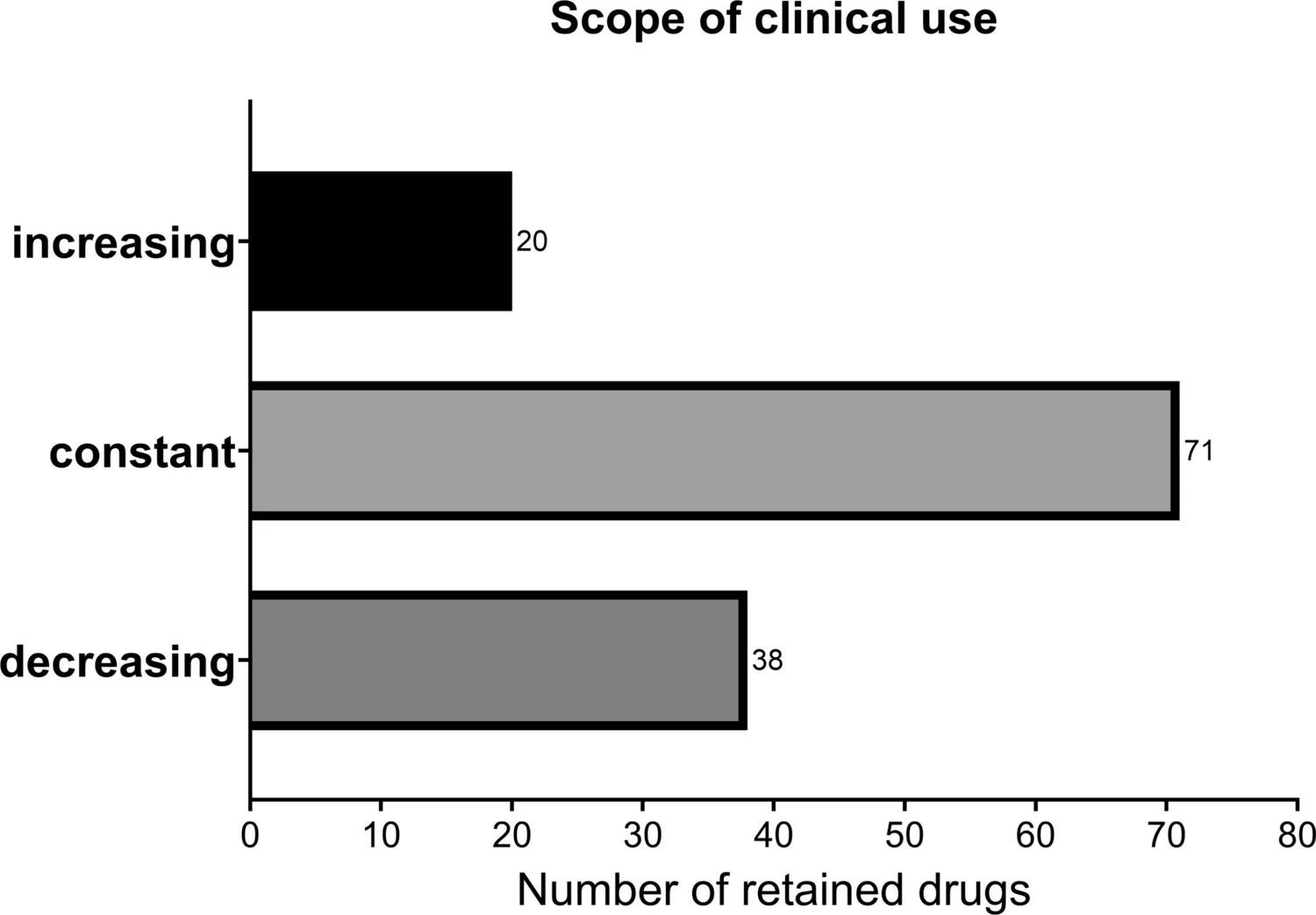
Overview about the assessment of the scope in clinical use for the retained drugs within current years (corresponding to Table S1). Drugs are categorized by increasing, constant, or decreasing use. Notably, also drugs with a decreasing scope of clinical use can be considered appropriate for listing on the EML, since they may be essential in particular settings and indications

The majority of drugs were classified as having a constant scope in clinical use in pharmacotherapy (*n* = 71, 55.0%) (Fig. 3, Table 2). This group includes drugs with continued relevance for their listed indications. Examples include activated charcoal for intoxication, benzathine benzylpenicillin for syphilis, doxycycline for several bacterial infections and malaria chemoprophylaxis, ethambutol for tuberculosis, cyclophosphamide and doxorubicin for cancer treatment, ferrous salt and folic acid for anaemia, lidocaine for arrhythmia and local anaesthesia, diazepam for anxiety disorders and seizures, morphine for pain, barium sulfate as radiocontrast medium, oral rehydration salts for diarrhoea, levothyroxine for thyroid hormone substitution, rabies vaccine for immunization, oxytocin as oxytocic, and glucose, sodium chloride, sodium lactate, and water for correction of water, electrolyte, and acid–base disturbances.

A smaller group of 20 drugs (15.5%) was classified as having increasing usage, mostly due to added indications, broadened usage fields, or increasing clinical demand. Examples include benzylpenicillin, flucytosine, paromomycin, rifampicin, methotrexate, acetylsalicylic acid, allopurinol, naloxone, betamethasone, dexamethasone, hydrocortisone, levothyroxine, prednisolone, measles vaccine, typhoid vaccine, furosemide, spironolactone, calcium gluconate, epinephrine, and salbutamol.

A further group was classified as having a declining scope of clinical use (*n* = 38, 29.5%). This group includes drugs with restricted indications, reduced preference, or a more limited current role compared with their original listing. Examples include amikacin, amphotericin B, sodium stibogluconate, sulfadiazine, sulfamethoxazole + trimethoprim, diazoxide, hydralazine, warfarin, amitriptyline, carbamazepine, phenobarbital, phenytoin, coal tar, tuberculin purified protein derivative, senna, ergometrine, mannitol, suxamethonium, and codeine.

### Identification of outdated and obsolete retained drugs

Most identified retained drugs remain established in clinical practice, justified by current evidence, guideline recommendations, and scope in clinical usage. However, 19 drugs were identified where continued listing on the EML warrants discussion. These drugs include dimercaprol, amodiaquine, ampicillin, chloramphenicol, erythromycin, griseofulvin, melarsoprol, oxamniquine, procaine benzylpenicillin, quinine, streptomycin, tetracycline, dextran, digoxin, dopamine, glyceryl trinitrate, miconazole, pilocarpine, and neostigmine (Table 3 and S1).

**Table 3:** Summary of retained drugs which are considered with questionable therapeutic relevance, requiring discussion for deletion in the next EML. For each drug, its current EML 2025 indication is provided, as well as information about guideline recommendations, evidence of efficacy and safety, and public health need. More detailed information can be found in Table S1. Drugs are sorted alphabetically

Obsolete retained drugs	Indication EML 2025	Guideline recommendation	Effectiveness and safety	Public health need
amodiaquine	malaria (curative, chemoprevention) in FDC	curative treatment of malaria, but not recommended for chemoprevention (MSF 2025c; Padberg 2015)	effective, but toxicity and fatal adverse events are an issue, increasing resistance (Schlitzer 2010; AlKadi 2007; White 2014; Nyunt And Plowe 2009)	not anymore recommended for long-term use, reserve malaria agent (Schlitzer 2010; Padberg 2015)
ampicillin	second choice for acute bacterial meningitis	only as an alternative for meningitis (Infectious Diseases Society of America 2025; Pfister et al. 2023)	decreasing efficacy due to high bacterial resistance rates, acceptable safety (Kaushik et al. 2014; Heinz 2018; FDA 2023)	not anymore recommended as first-choice, decreasing use (Infectious Diseases Society of America 2025; Pfister et al. 2023)
chloramphenicol	second choice for acute bacterial meningitis	only as reserve, not first choice for meningitis (EMC 2022; Moffa And Brook 2015; Sethi And Sethi 2025)	effective, but toxicity issues (e.g. aplastic anaemia) (Moffa And Brook 2015; Sethi And Sethi 2025; BfR 2014)	limited use (EMC 2022; Moffa And Brook 2015; Sethi And Sethi 2025)
dextran	plasma substitute	alternatives often preferred depending on indication (crystalloids, albumin) (Miao And Guthmiller 2025)	effective volume expander, but comparably risk of adverse events (anaphylaxis and coagulopathy), other crystalloids favoured for many indications (Isbister And Fisher 1980; Martin 2019; Rasmussen et al. 2015)	declining, historical use, now rarely used and crystalloids are first-line (Martin 2019; Baglin 2012)
digoxin	arrhythmia, heart failure	not first-line, partly used as an adjunctive therapy for atrial fibrillation and symptomatic heart failure, availability of superior alternatives (Crane et al. 2024; Chen et al. 2015)	evidence for symptomatic improvement but limited mortality benefit, narrow therapeutic index and arrhythmia risk require monitoring (Virgadamo et al. 2015; Jabri et al. 2022)	marginal importance because availability of superior alternatives (Ziff And Kotecha 2016; Crane et al. 2024; Chen et al. 2015)
dimercaprol	intoxication (antidote)	heavy metal intoxication (lead, arsenic, mercury) (Dawn And Whited 2023; Braitberg 2019)	effective but significant toxicity (Long 2020; Dawn And Whited 2023; REMM 2025)	declining usage because availability of more favourable alternatives like DMPS, problems with availability (Gerhardsson And Aaseth 2016; Whyte 2023)
dopamine	heart failure	not first-line in heart failure/acute treatment (Becerra et al. 2025; Bloom et al. 2023)	moderately effective but preference of norepinephrine and dobutamine, use is associated with higher mortality (Becerra et al. 2025; Varriale 1999; Hosoya et al. 2025; Zhou et al. 2017)	declining use, not recommended (Becerra et al. 2025)
erythromycin	ophthalmologic infections (*C. trachomatis, N. gonorrhoea*)	yes, but not effective in chlamydial infections, availability of alternatives (AAO 2025; Smith-Norowith et al. 2021; Duff 2015)	yes in susceptible pathogens in ocular use but increasing bacterial resistance, acceptable safety, availability of alternatives (AAO 2025; NHS 2022a; Kim And Toma 2011; Cabrera-Aguas et al. 2024; Hsueh 2005)	rarely used: already replaced in systemic therapy, availability of alternatives in ophthalmology (Hsueh 2005; Amsden 1996; Opthalmology Times 2009; Bremond-Gignac et al. 2011)
glyceryl trinitrate	angina	short-acting anti-anginal for acute chest pain, but preference of other alternatives (Arden 2011; Rousan And Thadani 2019; NICE 2016a)	symptomatic improvement, but contraindicated for long-term use (tolerance development), risks of severe adverse events (Pearson And Butler 2021; Arden 2011; Wee et al. 2015; Münzel et al. 2005)	declining use, alternatives like nitro-glycerine more favourable (NICE 2016a; Alyavi And Uzokov 2017)
griseofulvin	fungal infections	only in dermatophytes, replaced by other drugs in other indications (Aris et al. 2022; Bonifaz et al. 2024)	effective but comparable (dermatophytes) and inferior (other fungal infections) to newer drugs (Bar et al. 2019; Möhrenschlager et al. 2002)	declining use, not marketed anymore in many countries (DocCheck 2025; Bonifaz et al. 2024)
melarsoprol	trypanosomial infection (2nd stage African)	reserve in trypanosomiasis (Lindner et al. 2025)	decreasing effectiveness, high toxicity (Robays et al. 2008; Seixas et al. 2020)	declining, replacement by alternatives (Lindner et al. 2025)
miconazole	fungal infections, topical	alternative for topical treatment of superficial fungal infections but not first-line (Mayser And Niehaus 2023; Bortolussi and Martin 2024)	good efficacy but higher rate of adverse effects and drug interactions than available and favoured alternatives, available as OTC (GOV UK 2017; Gamil et al. 2024; Mijalijica et al. 2022; Watjer et al. 2023; Taudorf et al. 2019)	high global prevalence of superficial mycoses (Ma et al. 2025; Havlickova et al. 2008)
neostigmine	myasthenia gravis, muscle relaxant	alternative for reversal of neuromuscular blockade and myasthenia gravis, not first choice (Neely et al. 2025; Thilen et al. 2023)	inferior to suxamethonium in anesthesia and pyridostigmine in mystenia gravis, less effective and more adverse effects (Urban et al. 2018; Fiorda Diaz et al. 2022; Mehndiratta et al. 2014; Neely et al. 2025)	declining, but still used in some parts of the world (Urban et al. 2018)
oxamniquine	schistosomial infections	reserve, mostly replaced by praziquantel (Danso-Appiah et al. 2022)	effective in some settings, less broad spectrum than alternatives, increasing resistance (Alwan et al. 2023; Rugel et al. 2020; Chevalier et al. 2019)	rarely used, praziquantel preferred (Sisay et al. 2025; Danso-Appiah et al. 2022)
pilocarpine	miotic/glaucoma	historic alternative for acute angle-closure and chronic glaucoma, not recommended as first choice (Wagner et al. 2022)	effective but high number of adverse effects, availability of more favourable alternatives, not recommended as first choice (NICE 2022a; Li et al. 2025; Ludwig And Seifert 2025)	declining, replacement by newer drugs (Ludwig And Seifert 2025)
procaine benzylpenicillin	Access group: second choice syphilis	alternative but not first choice in syphilis (WHO 2016; CDC 2024b; MSHC 2021)	increasing bacterial resistance, inferior to other second choice alternatives and need of frequent injections (CDC 2024b; Public Health Agency of Canada 2024; Greenwood And Irving 2012)	rarely used, only if no other treatment is available (MSHC 2021)
quinine	malaria (curative)	not recommended in WHO guidelines for malaria, listed as alternative in some national guidelines (Visser et al. 2022)	substantial disadvantages in comparison to alternatives like artesunate (high toxicity, narrower therapeutic index, resistance issues and decreased efficacy, dosing) (Shanks 2016; Yeka et al. 2009; Cramer et al. 2011)	use not recommended, inferior to alternatives (Yeka et al. 2009; SEAQUAMAT 2005)
streptomycin	tuberculosis	second-line for tuberculosis, but preference of other reserve drugs (Ministry of Health And Family Welfare 2025; Cohen et al. 2020; Michalik et al. 2025)	high resistance rates, toxicity issues (ototoxicity, nephrotoxicity), preference of newer alternatives as first line and amikacin as second line (Cohen et al. 2020; Rocha et al. 2021; Guo et al. 2025; Niemann 2023; Waters And Tadi 2023)	reserve, declining use and withdrawal from guidelines (Cohen et al. 2020; Ministry of Health And Family Welfare 2025)
tetracycline	ophthalmologic infections (bacterial blepharitis, conjunctivitis, keratitis, trachoma)	only recommended as use in eye drops in ophthalmology but not recommended in systemic therapy (MSF 2025e)	effective in ophthalmologic conditions but availability of newer and favoured drugs, high bacterial resistance rates, unfavourable adverse effects in comparison with alternatives (Pearson et al. 2024; Xiong et al. 2024; Griffin et al. 2010; Bremond-Gignac et al. 2011)	niche, declining use and importance, replaced by more favourable antibacterial drugs (Semenova et al. 2025; LaPlante et al. 2022)

Several of the drugs considered for deletion belong to the anti-infective category. This includes amodiaquine, ampicillin, chloramphenicol, erythromycin, griseofulvin, melarsoprol, oxamniquine, procaine benzylpenicillin, quinine, streptomycin, and tetracycline. Other drugs considered for deletion are located in antidotes, blood components or plasma substitutes, cardiovascular treatment, dermatology, ophthalmology, and peripheral muscle relaxants.

Among the drugs considered for deletion, several are listed only for narrow or second-choice indications in the EML 2025. Examples include ampicillin and chloramphenicol for acute bacterial meningitis, procaine benzylpenicillin for syphilis, streptomycin for tuberculosis, quinine for malaria, oxamniquine for schistosomiasis, melarsoprol for trypanosomiasis, pilocarpine for glaucoma, and neostigmine for myasthenia gravis or reversal of neuromuscular blockade (Infectious Diseases Society of America 2025; EMC 2022; MSHC 2021; Ministry of Health And Family Welfare 2025; Visser et al. 2022; Danso-Appiah et al. 2022; Lindner et al. 2025; Mayser And Niehaus 2023; Becerra et al. 2025; Crane et al. 2024; Wagner et al. 2022; Neely et al. 2025; Thilen et al. 2023). In contrast to drugs with a declining scope of clinical use but still justified niche use, these drugs showed additional concerns such as weak current guideline position, relevant toxicity, reduced efficacy, or the availability of more favourable alternatives.

In addition to drugs considered for deletion, several drugs were identified where adjustment of specific listed indications may be required rather than deletion of the drug itself. This applies to metronidazole, sulfadiazine, hydrocortisone, and prednisolone. For these drugs, the assessment indicates that the drug remains relevant, while individual listed indications require reassessment.

## Discussion

### Interpretation of changes in listed indications of retained drugs

The present assessment shows that retained drugs do not represent a uniform group of unchanged medicines in terms of their therapeutic context and scope of clinical use. Retention on the EML between 1977 and 2025 demonstrates historical continuity, but not necessarily unchanged pharmacological relevance. Some drugs remained listed for essentially the same indication, while others were retained because their indication was specified, narrowed, expanded, or shifted to a different therapeutic field (Table S1, Fig. 2).

The specification of indications can partly be explained by the development of the EML itself. Earlier editions often used broader therapeutic categories, whereas later editions provide more precise categorizations. This is especially relevant for anti-infective and antineoplastic drugs, where rational pharmacotherapy requires exact assignment to pathogens, resistance patterns, tumour entities, or treatment regimens. For example, doxycycline is now assigned to defined infectious indications, while doxorubicin, cyclophosphamide, fluorouracil, vincristine, and methotrexate are linked to specified malignant diseases (Pearson et al. 2024; Holmes and Charles 2009; Kciuk et al. 2023; Gephart et al. 2025; Longley et al. 2003; Dhyani et al. 2022; Koźmiński et al. 2020). Therefore, specification does not necessarily indicate a change in importance, but rather a more precise description of current therapeutic use.

Narrowed indications were observed particularly for anti-infective drugs. A narrowed listing often reflects that a drug is no longer suitable for broad use, but may still retain value in a specific indication. This is evident for several anti-infective drugs. For example, ampicillin and chloramphenicol are listed in the EML 2025 only as second-choice options for acute bacterial meningitis, while procaine benzylpenicillin is restricted to second-choice use in syphilis (Infectious Diseases Society of America 2025; Pfister et al. 2023; EMC 2022; Moffa And Brook 2015; WHO 2016; CDC 2024b; MSHC 2021). Similar narrowing was observed for tetracycline, erythromycin, quinine, streptomycin, sulfadiazine, and amodiaquine (NHS 2022a; Bremond-Gignac et al. 2011; Semenova et al. 2025; AAO 2025; Duff 2015; Visser et al. 2022; Cohen et al. 2020; RKI 2018; Dunay et al. 2018; Padberg 2015). For these drugs, the current EML listing no longer reflects broad therapeutic use, but a restricted or reserve indication.

A change of usage field represents another important pattern. In these cases, continued listing is not primarily justified by the original indication, but by a newer therapeutic role. Diazoxide is now listed for hypoglycaemia, isosorbide dinitrate for angina, propranolol for migraine prophylaxis, and ephedrine for anaesthesia-related use (Chen et al. 2021; Newman-Lindsay et al. 2022; Rao et al. 2025; Qaseem et al. 2025; Versijpt et al. 2024; Kinsella et al. 2017). Conversely, drugs with added indications, such as epinephrine, salbutamol, spironolactone, furosemide, dexamethasone, hydrocortisone, and prednisolone, illustrate that older medicines may gain importance when their pharmacological mechanisms are useful across several clinical situations (Pashar et al. 2025; Wang And Lipner 2020; GINA 2025; GOLD 2025; Sinha et al. 2025; Resuscitation Council UK 2021; Heidenreich et al. 2022; Kitai et al. 2025; MSF 2025f; NHS 2025b; Pazderska And Pearce 2017; Kashyap And Chabri 2023; Walsh et al. 2025).

### Patterns in the change of scope in clinical usage

The assessment of the scope in clinical use between the 1^st^ and 24^th^ EML allowed a differentiation of drugs in constant, increasing, and declining developments (Table 2 and S1, Fig. 3). This categorization is more informative than historical retention alone, because it separates drugs with stable therapeutic reliance from drugs with expanded demand and from drugs whose utilization has become limited.

Drugs with constant scopes in clinical use usually address stable and fundamental therapeutic needs. Examples include activated charcoal for intoxication, lidocaine for arrhythmia and local anaesthesia, morphine for pain, oxytocin for obstetric use, oral rehydration salts for diarrhoea and rehydration, and potassium chloride, sodium chloride, sodium lactate, glucose, and water for correction of water, electrolyte, and acid–base disturbances (Zellner et al. 2019; Hartmann et al. 2025; Bahar And Yoon 2021; Güler et al. 2023; WHO 2018a; NICE 2016b; Kehl et al. 2021; Parry Smith et al. 2020; WHO 2006b; Munos et al. 2010; FDA 2014; Tinawi 2020; Miller et al. 2023; Ichai et al. 2014; Verma et al. 2024; EMA 2020). This is likely explainable through evidenced efficacy, acceptable safety, broad clinical applicability, and the absence of a need for more complex alternatives in many basic-care situations.

Drugs with increasing scopes of clinical use often show either expanded indications or growing relevance due to disease burden and treatment standards. Methotrexate is now important not only in cancer treatment but also in rheumatologic and dermatologic conditions, acetylsalicylic acid gained relevance in cardiovascular prevention, naloxone gained importance as a life-saving antidote in MOR agonist overdose, and calcium gluconate has an important role in hypocalcaemia, hyperkalaemia, and calcium-channel inhibitor intoxication (Dattani 2025; DEA 2025); Friedman And Cronstein 2019; Perrotta et al. 2024; Koźmiński et al. 2020; Rao et al. 2025; Kumbhani et al. 2025; Hennessy et al. 2024; EMCDDA 2015; Chakraborty et al. 2024; St-Onge et al. 2017). The increasing importance of levothyroxine, salbutamol, furosemide, and spironolactone is also related to high or increasing prevalence of hypothyroidism, chronic airway diseases, and cardiovascular diseases (Kahaly And Gottwald-Hostalek 2022; Centanni et al. 2025; GINA 2025; GOLD 2025; Savarese et al. 2023; Wu et al. 2024). Consequently, increasing scope of clinical use often reflects a combination of pharmacological versatility and epidemiological relevance. Table 4 summarizes information about the retained drugs that are considered as experiencing increasing scopes in their clinical usage.

**Table 4:** Overview about retained drugs which are considered with increasing scopes in clinical usage. For each drug, its current EML 2025 indication is provided, as well as information about guideline recommendations, evidence of efficacy and safety, and public health need. More detailed information can be found in Table S1. Drugs are sorted alphabetically

Retained drugs	Indication EML 2025	Guideline recommendation	Effectiveness and safety	Public health need
acetylsalicylic acid	pain, anti-inflammatory, acute migraine attack, anticoagulant, prevention of atherosclerotic CD, juvenile joint	yes, first-choice for prevention of CV, anticoagulation, moderate pain and rheumatic disorders (Rao et al. 2025; Kumbhani et al. 2025; Ciurtin et al. 2025; Amaechi et al. 2021)	strong evidence for analgesia and secondary prevention of CV, long-term adverse effects require risk–benefit assessment for primary prevention (Garcia And Aguiar 2025; Ciurtin et al. 2025; La Torre et al. 2025; Schachtel et al. 2010; Yilmaz et al. 2022)	widely used for analgesia and CV prevention (Montinari et al. 2019; Garcia And Aguiar 2025)
allopurinol	tumour lysis syndrome, gout	yes (NICE 2022b; Chan et al. 2025)	evidence for gout prophylaxis and preventing hyperuricaemia in tumour lysis syndrome contexts, acceptable safety, hypersensitivity syndrome rare but serious (Cortes et al. 2010; Yang et al. 2015)	widely used in gout and importance in oncology (Yang et al. 2015)
benzylpenicillin	first-choice severe community-acquired pneumonia, complicated severe acute malnutrition, sepsis in neonates and children, syphilis	first line for susceptible streptococcal infections and neonatal sepsis (Fuchs et al. 2018; Marum et al. 2021; Bennett et al. 2012)	efficacy and good safety, narrow-spectrum (Rote Liste 2025a; Marum et al. 2021)	high need and widely used (Bennett et al. 2012; Bos et al. 2017)
betamethasone	antiinflammatory, antipruritic, topical	topical GCR agonist for inflammatory dermatoses (Wollenberg et al. 2025)	strong efficacy for inflammatory skin diseases, local atrophy and systemic absorption risks with prolonged use, steroid-sparing strategies considered for long-term use (Wollenberg et al. 2025; Gisondi et al. 2024; Pofe et al. 2023)	widely used in dermatology and rheumatology, importance in many short-term inflammatory conditions (Benzon et al. 2025)
calcium gluconate	antidote, nutritional supplement	treatment of hyperkalaemia and Ca-channel blocker toxicity, correction of hypocalcaemia, osteoporosis treatment (NOGG 2024; Chakraborty et al. 2024; Medicines And Healthcare products Regulatory Agency 2023; St-Onge et al. 2017)	benefit in emergencies, effective in deficiencies, part of prevention regimes for osteoporosis in risk groups, mild adverse effects but risk of drug interactions (Shea et al. 2004; UKCPA 2025; Chakraborty et al. 2024; St-Onge et al. 2017)	relevance in emergency and osteoporosis treatment
dexamethasone	allergic reaction, hormone replacement, cancer treatment (ALL, anaplastic large cell lymphoma, Burkitt lymphoma, multiple myeloma), emesis	widely recommended for multiple indications: as part of chemotherapy regimens, for severe allergic reactions, cerebral edema, and in specified severe respiratory inflammatory conditions (MSF 2025f; NHS 2025b)	robust evidence across many indications including oncology adjuncts and life-saving in pediatric emergency, adverse effects significant but acceptable in indicated regimens (Giolito et al. 2025; Ottaiano et al. 2025; Mo et al. 2024; An et al. 2024; Weinstein et al. 2024)	high need in oncology and emergency medicine (Ottaiano et al. 2025; Weinstein et al. 2024)
epinephrine	asthma, COPD, local anaesthetic, allergic reactions, arrhythmias, mydriatic	first-line vasopressor in cardiogenic shock and anaphylaxis (Sinha et al. 2025; Resuscitation Council UK 2021; GINA 2025)	effective in cardiogenic shock and symptom relief in acute respiratory obstruction, rapid onset, adverse effects acceptable (Bernstein et al. 2025; Lüsebrink et al. 2024; Numa et al. 2000)	life-saving, widely used in emergency settings
flucytosine	fungal infections	yes (Sigera And Denning 2023; Harsanyi et al. 2017)	effective but toxicity requires monitoring (Folk et al. 2016)	importance in severe fungal infections (Harsanyi et al. 2017; Sprute et al. 2023)
furosemide	diuretics, heart failure	first-line NKCC inhibitor in heart failure, symptomatic treatment in chronic kidney disease (KDIGO 2024; Kitai et al. 2025)	effective in diuresis and reduction of fluid volume, symptom relief, mortality/morbidity benefit in heart failure, caution about adverse effects (e.g. hypovolemia, hypokalemia) and drug interactions (Song et al. 2025; Ho And Power 2010)	widely used, globally high disease prevalence and need (Wu et al. 2024; Magdy et al. 2022)
hydrocortisone	adrenal hormone substitution, anti-inflammatory (gastrointestinal, dermatology/topical), cancer treatment (ALL, Burkitt lymphoma), allergic reaction	first-line replacement for adrenal insufficiency, cancer treatment and inflammatory conditions, but not recommended as standard in allergic shock (Pazderska And Pearce 2017; Whyte et al. 2022; Das a+nd Panda 2017; Ornetti et al. 2025)	lifesaving adrenal replacement and effective in short-term anti-inflammatory effects, strong probability of chronic systemic adverse effects (Martinez And Sabatier 2025; Aldea et al. 2020; Das And Panda 2017; Ornetti et al. 2025; Yasir et al. 2023)	essential in inflammatory conditions, endocrine disorders and part of cancer treatment regimens (Yasir et al. 2023)
levothyroxine	thyroid hormone substitution	hypothyroidism (Centanni et al. 2025)	effective and safe when dosed and monitored appropriately, essential for brain development in children, need of individual dosage adjustment and control of TSH values (Centanni et al. 2025; Colucci et al. 2013; Esposito et al. 2022)	essential in maintenance treatment of hypothyroidism, prevention of mental retardation in congenital hypothyroidism, high and expanding prevalence (Kahaly And Gottwald-Hostalek 2022; Seo et al. 2017)
measles vaccine	vaccination	routine childhood immunization (WHO 2013)	effective and safe, prevents high morbidity/mortality, mild and transient adverse effects (e.g. fever) possible (Dattani 2025; Benn And Aaby 2023; Dattani And Spooner 2025; Government of Canada 2025b)	indispensable for global measles elimination goals (WHO 2013)
methotrexate	cytotoxic (ALL, acute promyelocytic leukaemia, anaplastic large cell lymphoma, Burkitt lymphoma, early stage breast cancer, gestational trophoblastic neoplasia, Langerhans cell histiocytosis, osteosarcoma), dermatology (not specified), anti-rheumatic (DMARDs), juvenile joint diseases	yes (Perrotta et al. 2024; Koźmiński et al. 2020)	efficacy in cancer and rheumatology, but adverse effects and toxicity require monitoring (Perrotta et al. 2024; Koźmiński et al. 2020; Taylor et al. 2021; Hamed et al. 2022)	widely used (Koźmiński et al. 2020; Friedman and Cronstein 2020)
naloxone	antidote	antidote for MOR agonist overdose/induced respiratory depression (WHO 2025c)	evidence for life-saving reversal of opioid overdose, rapid onset but short half-life, minimal adverse effects relative to benefit (Hennessy et al. 2024; EMCDDA 2015; Rzasa Lynn And Galinkin 2018)	important in emergency care and public health/opioid use disorder (DEA 2025); Hennessy et al. 2024; EMCDDA 2015)
paromomycin	leishmaniac infections	yes (Zhang et al. 2025)	effective and acceptable safety (Kim et al. 2009; Salah et al. 2013; Matos et al. 2020)	increasing importance in endemic areas (Zhang et al. 2025; Sundar And Chakravarty 2008)
prednisolone	adrenal hormone substitution, anti-inflammatory (gastrointestinal, ophthalmology/topical), cancer treatment (ALL, anaplastic large cell lymphoma, Burkitt lymphoma, CLL, diffuse large B-cell lymphoma, follicular lymphoma, Hodkin lymphoma, Langerhans cell histiocytosis, metastatic castration-resistant prostate cancer, multiple myeloma), allergic reaction, seizues, cluster headache	systemic GCR agonist for many inflammatory, oncologic supportive and neurologic symptomatic uses, but not initial therapy in anaphylactic shock (Faggiano et al. 2022; Kashyap And Chabri 2023; Walsh et al. 2025)	robust across many acute and chronic inflammatory and oncologic indications, in chronic use adverse effects expectable (Faggiano et al. 2022; Wijdicks 2024; Obermann And Holle 2020; Kashyap And Chabri 2023; Becker And Kaindl 2023; Offor et al. 2025)	widely used across many medical fields
rifampicin	leprosy, tuberculosis	yes (Sivakumaran et al. 2024; Haigh et al. 2024)	effective and acceptable safety, use in combination therapies because of bacterial resistance risk (Sivakumaran et al. 2024; Richardus et al. 2021; Li et al. 2022; Kim et al. 2023a; Menzies et al. 2023)	widely used globally (Haigh et al. 2024; Government of Canada 2025a, b)
salbutamol	asthma, COPD	rescue bronchodilator for asthma and COPD (GINA 2025; GOLD 2025)	short-acting beta-agonist for acute symptom relief, effective in short-term treatment but possible cardiovascular adverse effects and tolerance development in chronic use (GOLD 2025; GINA 2025; Ma et al. 2023; Haney And Hancox 2005; Marques And Vale 2022)	high prevalence, widespread use and important treatment in acute situations, risk of overuse and worsening outcomes (Nwaru et al. 2020)
spironolactone	diuretics, heart failure	first-line MRA for heart failure with reduced ejection fraction, treatment-refractory hypertension (Heidenreich et al. 2022; Carey et al. 2018)	universal recommendation in heart failure, mortality reduction, but adverse effects (e.g. hypercalemia) require caution (Heidenreich et al. 2022; Chen et al. 2020; Prashar et al. 2025; Kosmas et al. 2018; Wang And Lipner 2020)	widely used, high prevalence and burden of disease (Savarese et al. 2023; Kosmas et al. 2018)
typhoid vaccine	vaccination	prevention of typhus in endemic settings and high-risk travel (Brendan et al. 2015; WHO 2018c)	safe and effective, reduction of morbidity and mortality (Gloeck et al. 2025; Batool et al. 2024)	important in typhoid-endemic countries (Khan et al. 2017)

Drugs with declining scopes in clinical use form a heterogeneous group. However, they should not be interpreted as automatic obsolescence, because they may remain relevant in low-resource settings, or if first-choice alternatives are not available. This applies to phenobarbital, phenytoin, carbamazepine, warfarin, hydralazine, suxamethonium, and mannitol (Brodie And Kwan 2012; Bankole et al. 2024; Nevitt et al. 2019; ILAE 2020; Chin And Doogue 2025; Tran et al. 2025; NICE 2023; Mech And Seifert 2025; Ahmad et al. 2025; Kim et al. 2023b).

In this context, it should also be acknowledged that changes in the scope of the clinical use or replacement by more favourable alternatives are not exclusively determined by intrinsic therapeutic value. Usage patterns may additionally be influenced by external factors such as pricing, availability, reimbursement policies, guideline adoption, and pharmaceutical marketing, which may affect prescribing behaviour independently of comparative efficacy or safety (Bindel and Seifert 2025a, 2025b).

### Reasons of obsolescence of initially essential retained drugs

Several drugs were identified for which continued EML listing warrants discussion (Table 3 and S1). The most prominent group consists of older anti-infective drugs, because antimicrobial usefulness changes over time as resistance patterns, pathogen epidemiology, and preferred treatment regimens change. Reduced efficacy or resistance is central for ampicillin, erythromycin, tetracycline, streptomycin, quinine, and amodiaquine (Kaushik et al. 2014; Heinz 2018; Hsueh 2005; Bremond-Gignac et al. 2011; Cabrera-Aguas et al. 2024; Semenova et al. 2025; Cohen et al. 2020; Rocha et al. 2021; Guo et al. 2025; Shanks 2016; Yeka et al. 2009; SEAQUAMAT 2005; Visser et al. 2022). In these cases, the argument for deletion is based on a weaker benefit–risk and efficacy profile compared with available alternatives.

Toxicity is another major reason. Dimercaprol is limited by significant toxicity and declining use due to the availability of less toxic alternatives, chloramphenicol is limited by rare but severe adverse effects such as aplastic anaemia, melarsoprol by high toxicity, and digoxin by limited mortality benefit, a narrow therapeutic index, and arrhythmia risk (Long 2020; Dawn And Whited 2023; Gerhardsson and Aesseth 2016; Mückter et al. 1997; Moffa and Brook 2015; Sethi And Sethi 2025; BfR 2014; Seixas et al. 2020; Lindner et al. 2025; Virgadamo et al. 2015; Jabri et al. 2022; Ziff And Kotecha 2016). A toxic drug can remain essential if it fills an unmet need, but it is considered inferior or obsolete when safer and similarly or more effective alternatives become available.

A further pattern is inferiority compared with treatment alternatives. Dextran, dopamine, pilocarpine, and neostigmine remain pharmacologically effective, but are often less favourable than available alternatives in their respective indications (Martin 2019; Miao And Guthmiller 2025; Becerra et al. 2025; Bloom et al. 2023; Wagner et al. 2022; NICE 2022a; Urban et al. 2018; Hristovska et al. 2017; Jacob et al. 2025). Compromised availability also contributes to reduced relevance, as seen with griseofulvin and dimercaprol (DocCheck 2025; Bonifaz et al. 2024; Gerhardsson and Aesseth 2016; Whyte 2023).

Furthermore, some drugs are not obsolete themselves, but specific listed indications require correction. Metronidazole remains important in anaerobic and protozoal infections, but its role in *C. difficile* infection has changed (NICE 2021; van Prehn et al. 2021; Hernández Ceruelos et al. 2019). Sulfadiazine remains relevant in toxoplasmosis but is not equally supported for all listed anti-infective indications (RKI 2018; Dunay et al. 2018). Hydrocortisone and prednisolone remain essential GCR agonists, but are not the initial life-saving treatment in anaphylactic shock, where epinephrine is considered as first-line (Whyte et al. 2022; Liyanage et al. 2017; Sinha et al. 2025; Resuscitation Council UK 2021).

### Categorization by therapeutic value

**Fig. 4:**
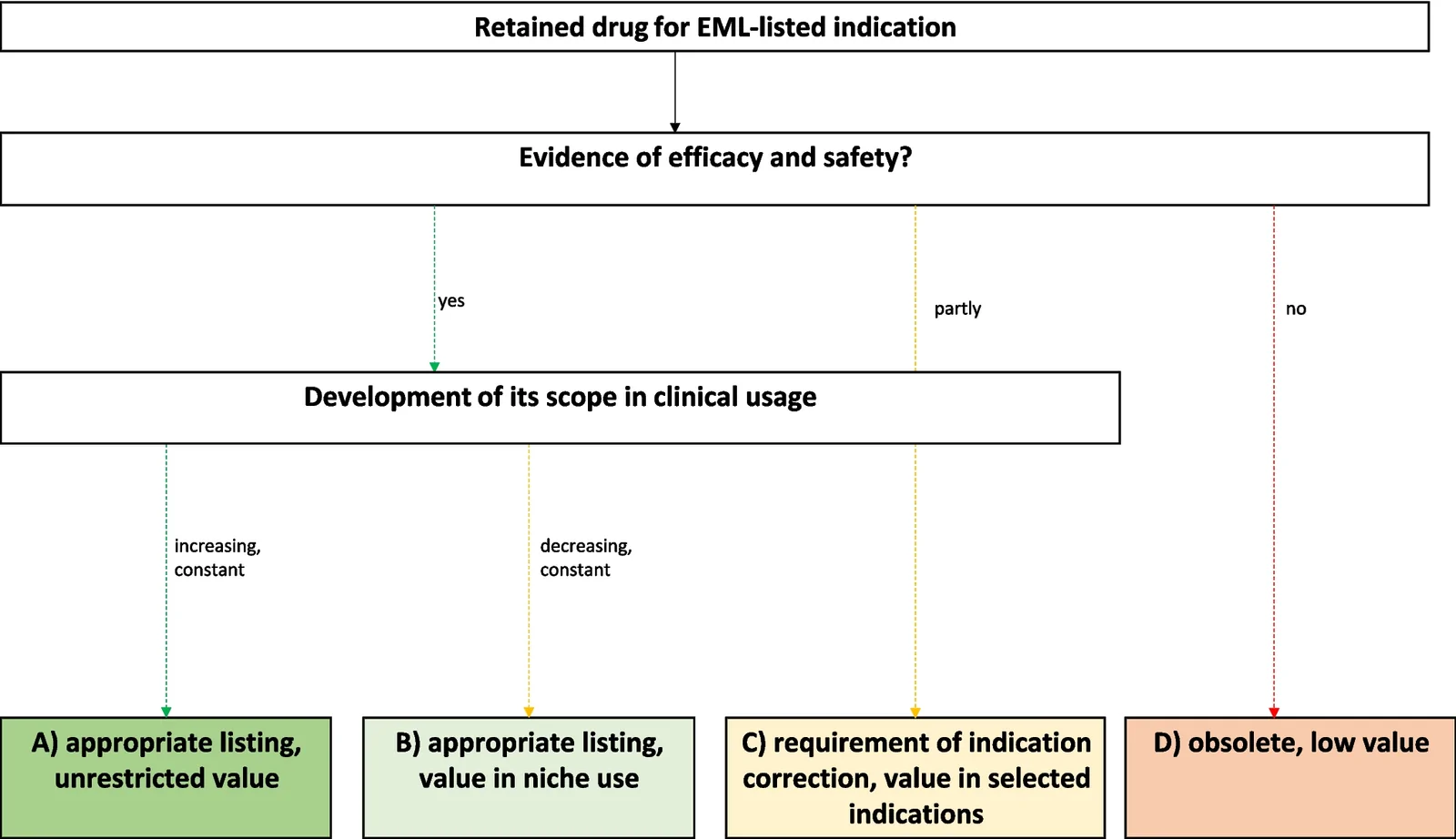
Overview of the assessment algorithm used to categorize retained drugs according to their therapeutic value for EML-listed indications (corresponding to Table 5). Drugs were first assessed regarding evidence of efficacy and safety for their listed indication. If evidence and safety were supported, the development of the scope of clinical use was considered and drugs were categorized as having appropriate listing with unrestricted value or appropriate listing with value in niche use. If evidence and safety were only partly supported, drugs were categorized as requiring correction of listed indications but retaining value in selected indications. If evidence and safety were not supported, drugs were categorized as obsolete and of low therapeutic value

Beyond the assessment of individual criteria, such as indications, development of the scope in clinical use and appropriateness of EML listing, broader patterns of current therapeutic value can be derived for retained drugs. These patterns allow a more transparent interpretation, resulting in a categorization in four groups: (A) unrestricted valuable, (B) valuable in niche use, (C) valuable for selected indications but requiring indication correction, and (D) low value and obsolete. Figure 4 visualizes the algorithm, while Table 5 summarizes characteristic patterns of the four categorizations.

**Table 5:** Typical patterns of therapeutic value observed among retained drugs. Summarized are characteristic patterns that were observed in the four assigned categories. Patterns are described according to changes in EML-listed indications, evidence of efficacy, benefit–risk profile, guideline recommendations, development of the scope in clinical use, and appropriateness of continued EML listing (corresponding to Table 2). Furthermore, a brief explanation and drugs are provided. A simplified algorithm for the assignment of therapeutic value categories can be found in Fig. 4

Criterion	A) Listing justified and clearly valuable	B) Listing justified, but niche	C) Listing justified, but indication correction needed	D) Listing not justified, obsolete
Change in EML-listed indications	unchanged, specified, added	unchanged, specified, narrowed	unspecific	unspecific
Evidence of efficacy	robust evidence for the listed indication	robust evidence for use in selected settings and reserve situations	evidence for some indications; but weak or no evidence for other specific EML-listed indications	weak evidence or reduced efficacy (loss of efficacy or inferior in comparison with alternatives)
Benefit–risk profile	acceptable safety profile, favourable benefit–risk ratio	acceptable safety profile, benefit–risk ratio depending on indication and setting	acceptable safety profile, favourable benefit–risk ratio for appropriate indications	issues in safety profile, relevant toxicity, narrow therapeutic index, poor benefit–risk ratio
Guideline recommendation	first-line, standard treatment, broadly recommended option	reserve option, second-choice treatment, first-choice in specific settings	guideline support exists for selected uses, but not for the problematic listed indication	no longer recommended, rarely used, replaced by better alternatives, only historical use
Development of scope of clinical use	constant, increasing	constant, declining	unspecific	declining
Appropriateness of EML listing	retained	retained	retained, but changes in listed indications	deleted
Interpretation	justified EML listing, essential therapeutic role	justified EML listing only with clearly defined indication or setting-specific justification	justified EML listing, but correction/specification of listed indications	questionable EML listing, requires discussion for deletion
Examples of retained drugs	acetyl salicylic acid, epinephrine, measles vaccine, methotrexate, naloxone, spironolactone	coal tar, phenobarbital, diazoxide, ergometrine, hydralazine, phenytoin	metronidazole, sulfadiazine, hydrocortisone, tuberculin (PPD), suxamethonium	dextran, chloramphenicol, quinine, tetracycline, pilocarpine, neostigmine

Retained drugs with broad and preserved therapeutic value (category A) are characterized by robust evidence of efficacy for their listed indications, an acceptable safety profile, established scope in clinical use, and a favourable benefit–risk ratio (magnitude of benefit). Acetylsalicylic acid illustrates this pattern. It has retained and expanded its relevance because it is used not only as an analgesic and antipyretic drug, but also in cardiovascular prevention, acute cardiovascular care, and selected rheumatic disorders (Rao et al. 2025; Kumbhani et al. 2025; Ciurtin et al. 2025; Amaechi et al. 2021). Its continued relevance is also reflected by a broad scope in clinical use in pain management and by its established role in the prevention of cardiovascular events (Montinari et al. 2019; Garcia And Aguiar 2025). Another example is epinephrine, which is essential in emergency situations such as anaphylaxis and resuscitation, but is also used in obstructive airway disease, local anaesthesia, and ophthalmological indications. Due to its rapid onset of effect and life-saving pharmacological actions, epinephrine remains a highly valuable drug in acute care and emergency pharmacotherapy (Bernstein et al. 2025; Lüsebrink et al. 2024; Numa et al. 2000).

Drugs that remain valuable, but mainly in selected indications, reserve situations, or specific settings (category B) are usually characterised by declining general importance, but this does not make them automatically obsolete. Coal tar is an example. It is effective in psoriasis, but its use has declined in high-resource settings because of low convenience for patients, cosmetic disadvantages, and the availability of other effective alternatives such as calcipotriol (Chan 2025; Slutsky et al. 2010; Paghdal And Schwartz 2009; Elmets et al. 2021; Singh et al. 2013). Nevertheless, due to low costs, availability, and efficacy, coal tar is relevant in low-income settings (Paghdal And Schwartz 2009). Phenobarbital represents another example. In many high-income countries, it has been replaced by newer antiseizure drugs for long-term seizure prevention and is mainly used in specific situations, such as status epilepticus (Pressler et al. 2023; NICE 2025b). However, because of its efficacy, low cost, availability, and long clinical experience, phenobarbital remains important for seizure prevention in many LMICs (Brodie And Kwan 2012; Bankole et al. 2024).

Some drugs remain pharmacologically valuable, but for individual EML-listed indications require correction or specification (category C), as seen in metronidazole and sulfadiazine. Metronidazole remains important for anaerobic and protozoal infections, but it is not anymore recommended in the treatment of *C. difficile* infection (NICE 2021; van Prehn et al. 2021). However, for its first-choice indications, it is widely used and has an acceptable safety and efficacy (Hernández Ceruelos et al. 2019). Sulfadiazine remains relevant in toxoplasmosis, where it is part of recommended combination treatment regimens, but it is not equally supported for broader anti-infective indications such as malaria (RKI 2018; Dunay et al. 2018; CDC 2024c); Castaño et al. 2022; Konstantinovic et al. 2019).

Drugs that are considered obsolete (category D) have a low current therapeutic value, compared with treatment alternatives. This includes dextran, which has largely been replaced by safer and equally effective plasma substitutes (Miao And Guthmiller 2025). Although it is effective as a plasma volume expander, its use is limited by serious adverse effects, making it more a historical than a current treatment option (Martin 2019; Baglin 2012). Chloramphenicol represents another example. It remains effective against susceptible pathogens, but is now mainly restricted to reserve use, for example in bacterial meningitis (EMC 2022). Its clinical role is strongly limited by relevant toxicity, including rare but severe haematological adverse effects, and by the availability of safer and more favourable alternatives (Moffa and Brook 2015; Sethi And Sethi 2025; BfR 2014).

The categorization by therapeutic value separates two central patterns. On the one hand, many retained drugs are still clearly valuable and remain important because of reproducible efficacy, clinical indispensability, and broad therapeutic use. On the other hand, some retained drugs show limited current value, either because their role has become restricted to niche situations or because their benefit–risk profile is no longer favourable compared with available alternatives. Therefore, the assessment of EML-listed indications does not only support conclusions on appropriate or questionable EML listing, but also allows a broader interpretation of the current therapeutic value of long-retained drugs.

### Limitations and strengths

The analysis has several limitations, mainly related to scope, assessment criteria, and comparability of sources. It focuses on retained drugs listed in both the 1^st^ and 24^th^ editions of the EML and therefore does not provide a full longitudinal assessment of all intermediate EML editions. The pharmacological assessment was based on listed indications, guideline recommendations, evidence of efficacy and safety, scope of clinical usage, and public health need. However, these aspects differ between countries, guidelines, and health-care settings, and the classification into constant, increasing, or declining scope of clinical use and therapeutic value cannot be completely objective. Because of the large number of drugs and aspects included in the analysis, it was not feasible to perform a fully systematic literature search. Although publications representing different healthcare settings and viewpoints were considered where possible, bias in the included references cannot be completely excluded. Excluded aspects of the assessment include dosing, formulations, paediatric use, cost-effectiveness, national EML modifications, and the distinction between core and complementary lists. Furthermore, a conceptual limitation relates to the operationalisation of “therapeutic value” categories. In particular, the distinction between “robust evidence” and “magnitude of benefit” cannot be fully separated within the applied framework, and the categorisation of drugs was primarily based on available evidence certainty, guideline interpretation, and overall benefit–risk considerations. In this context, the assessment of “favourable benefit–risk ratio” should be understood as a pragmatic categorisation rather than a strict binary distinction.

A key strength of this analysis is the pharmacologically orientated reassessment of long-standing retained drugs beyond their historical preservation on the EML. Instead of considering retention alone as proof of unchanged therapeutic value, the analysis evaluates the current listed indications, evidence of efficacy and safety, guideline support, and scope of clinical usage. This allows an evaluation of drugs and indications, whether they require discussion for deletion or correction. Furthermore, an algorithm-based, transparent assignment to therapeutic value categories was applied. The analysis highlights both the importance of many long-established drugs and the need for regular reassessment to ensure that retained drugs remain clinically justified, therapeutically relevant, and consistent with the fundamental purpose of the EML: to provide a rational basis for essential pharmacotherapy.

## Conclusions

The present analysis shows that retained drugs can be characterised by distinct patterns (Table 1, 5 and S1, Fig. 2–4). Many drugs retained constant scopes in clinical use and remain part of basic care. Their therapeutic value is therefore not primarily based on novelty, but evidence of pharmacological efficacy and broad scope in clinical use. Some drugs gained relevance through expanded indications, pharmacological versatility, or increasing epidemiological demands. Conversely, several drugs show declining use. However, declining scopes in clinical uses is not equivalent to obsolescence, since some drugs retain relevant niche, reserve, or low-resource value. Therefore, the trend in the scope of clinical use has to be distinguished from the therapeutic value of a drug. 19 retained drugs were identified where a deletion from the EML requires discussion. Main reasons for obsolescence are restricted or second-choice indications, reduced efficacy, pathogen resistance, toxicity, declining availability, or replacement by more favourable alternatives (Table S1).

**Fig. 5:**
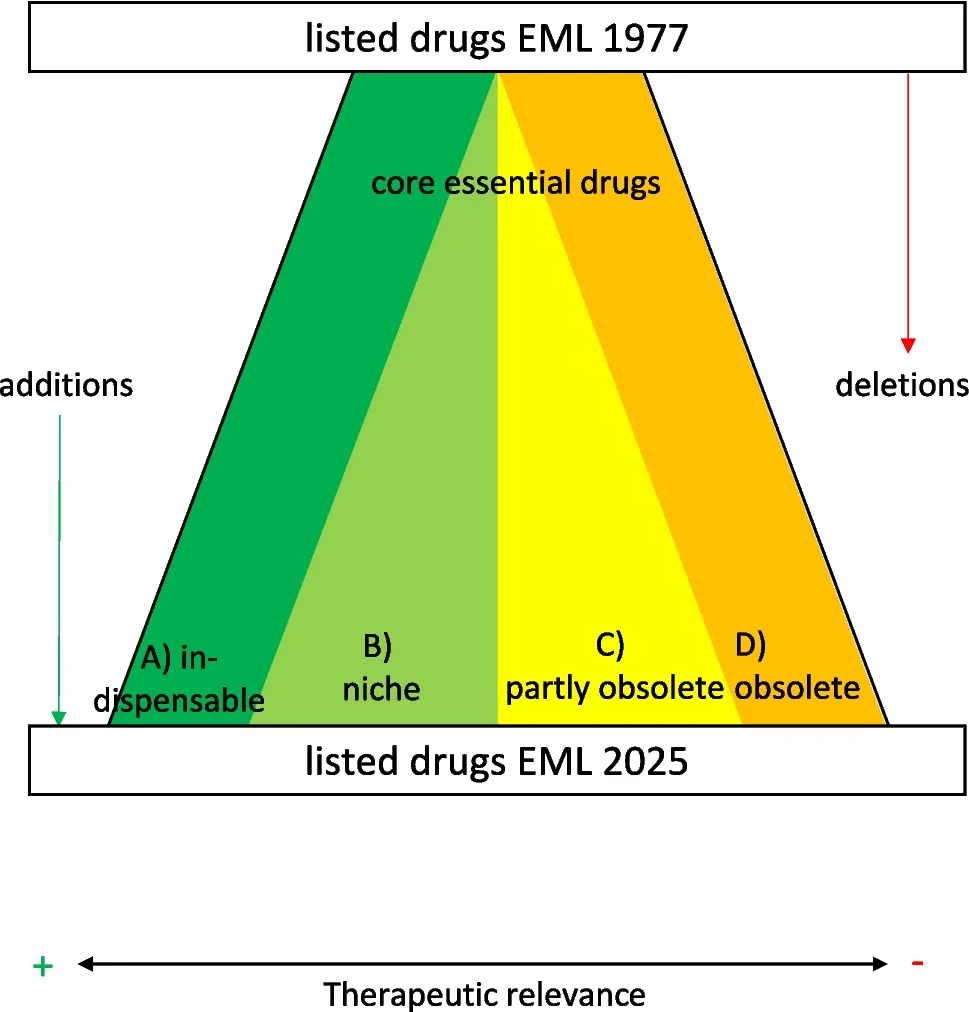
Spectrum of therapeutic relevance among retained drugs. The trapezoid model visualizes that long-term EML retention does not exclusively represent universally accepted and indispensable medicines, but rather an increasing heterogeneous continuum of therapeutic relevance. The horizontal axis represents decreasing therapeutic relevance from left to right. The left side of the spectrum (dark/light green) reflects drugs with high current relevance and strong justification for continued EML inclusion, whereas the right side (yellow/orange) reflects drugs with declining relevance and questionable continued EML listing

The structured assessment algorithm of therapeutic value (Fig. 4) resulted in four groups of retained drugs: clearly valuable, valuable in niche use, indication correction needed, and low value or obsolete. This distinction is important because long-term retention can indicate true therapeutic value, but can also preserve drugs whose current role is restricted, outdated, or no longer sufficiently justified (Table 5). Contrary to the expectation that retained drugs would exclusively represent universally accepted and indispensable medicines, the analysis demonstrated a heterogeneous spectrum ranging from indispensable to obsolete drugs. Figure 5 visualizes a simplified model which summarizes the central finding of the study that historical retention on the EML should not be interpreted as a direct equivalent of unchanged therapeutic importance.

Future EML revisions should not only evaluate whether a drug remains on the EML, but whether each listed indication is still supported by current evidence, guideline recommendations, risk–benefit ratios, scope of clinical usage, and therapeutic value. Drugs with clearly unfavourable profiles and available superior alternatives should be discussed for deletion. Drugs relevant mainly in niche or low-resource settings should remain listed when justified, but their setting-specific role should be explicitly stated. Beyond clinical and pharmacological considerations, it should also be acknowledged that incentive structures may influence the expansion of listed drugs on the EML. In particular, the development of new drugs or the generation of additional evidence supporting expanded indications is highly supported by academic research and commercial interest. In contrast, there are comparatively limited or less explicit incentives for the systematic reassessment and potential removal of older medicines, even when their relative therapeutic value may have declined. This asymmetry may contribute to a tendency towards expansion of the EML over time.

In the future, the EML will require a careful balance between rational drug selection and the inclusion of innovative therapies. This is particularly relevant with the increasing introduction of highly specialized and costly treatments, raising the question of whether such medicines can be considered essential in addition to being rational for a specific condition. Therefore, it is proposed that EML chapters are reviewed on a regular basis rather than primarily through ad hoc recommendations to the Committee. Furthermore, additions and deletions should not be initiated solely through external applications, but the Committee should also proactively identify and address relevant issues, for example through structured consensus-based approaches such as a Delphi process (WHO 2025a; Hasson et al. 2025). Lastly, the decision-making process should be made transparent and clearly explained. While this is generally well addressed in technical reports, the most recent technical report from 2025 lacks comprehensiveness in this regard (WHO 2025a). Such approaches would preserve the global function of the EML while improving its pharmacological precision and ensuring that retained drugs and all other included drugs on the EML remain not only historically important, but clinically and therapeutically justified.

## Supplementary Information

Below is the link to the electronic supplementary material.

ESM 1 (DOCX 219 KB)

## Data Availability

All source data for this study are available upon reasonable request from the authors.

## References

[CR1] Ahmad I, El-Boghdadly K, Iliff H et al (2025) Difficult airway society 2025 guidelines for management of unanticipated difficult tracheal intubation in adults. Br J Anaesth. 10.1016/j.bja.2025.10.006 41203471

[CR2] Aldea M, Orillard E, Mansi L, Marabelle A, Scotte F, Lambotte O, Michot JM (2020) How to manage patients with corticosteroids in oncology in the era of immunotherapy? Eur J Cancer 141:239–251. 10.1016/j.ejca.2020.09.032 33212339

[CR3] AlKadi HO (2007) Antimalarial drug toxicity: a review. Chemotherapy 53(6):385–91. 10.1159/000109767 17934257

[CR4] Alwan SN, Taylor AB, Rhodes J, Tidwell M, McHardy SF, LoVerde PT (2023) Oxamniquine derivatives overcome Praziquantel treatment limitations for Schistosomiasis. PLoS Pathog 19(7):e1011018. 10.1371/journal.ppat.1011018 37428793 PMC10359000

[CR5] Alyavi A, Uzokov J (2017) Treatment of stable angina pectoris: focus on the role of calcium antagonists and ACE inhibitors. e-J Cardiol Pract 15(N° 9). https://www.escardio.org/Journals/E-Journal-of-Cardiology-Practice/Volume-15/Treatment-of-stable-angina-pectoris-focus-on-the-role-of-calcium-antagonists-and-ACE-inhibitors#. Last updated 5 July 2017. Last accessed 1 Dec 2025

[CR6] Amaechi O, McCann M, Featherstone K (2021) Pharmacologic therapy for acute pain. Am Fam Physician 104(1):63–72. https://www.aafp.org/pubs/afp/issues/2021/0700/p63.html 34264611

[CR7] American Academy of Opthalmology (AAO) (2025) Use of erythromycin in the prevention of ophthalmia neonatorum - 2025. American Association for Pediatric Ophthalmology and Strabismus and Academy Secretary for Quality of Care. https://www.aao.org/education/clinical-statement/use-of-erythromycin-in-prevention-of-neonatal-opht. Last updated Oct 2025. Last accessed 29 Nov 2025

[CR8] Amsden GW (1996) Erythromycin, clarithromycin, and azithromycin: are the differences real? Clin Ther 18(1):56–55. 10.1016/s0149-2918(96)80179-2 8851453

[CR9] An AW, Chen X, Urbauer DL, Bruera E, Hui D (2024) Impact of dosing and duration of dexamethasone on serious corticosteroid-related adverse events. JPSM 67(1):59–68. 10.1016/j.jpainsymman.2023.09.021 37769822

[CR10] Arden C (2011) The management of stable angina. Br J Cardiol 18(Suppl 3):s1-s12. 10.5837/bjc.2011.s02

[CR11] Aris P, Wei Y, Mohamadzadeh M, Xia X (2022) Griseofulvin: an updated overview of old and current knowledge. Molecules (Basel, Switzerland) 27(20):7034. 10.3390/molecules27207034 36296627 PMC9610072

[CR12] Baglin T (2012) Chapter 29 - Drugs and haemostasis. Clinical Pharmacology (Eleventh Edition). Churchill Livingstone pp 482–495. 10.1016/B978-0-7020-4084-9.00068-9

[CR13] Bahar E, Yoon H (2021) Lidocaine: a local anesthetic, its adverse effects and management. Medicina (B Aires) 57(8):782. 10.3390/medicina57080782 PMC8399637 34440986

[CR14] Bankole NDA, Dokponou YCH, De Koning R, Dalle DU, Kesici Ö, Egu C, Ikwuegbuenyi C, Adegboyega G, Yang Ooi, SZ, Dada OE, Erhabor J, Mukambo E, Olobatoke TA, Takoutsing BD Bandyopadhyay S (2024) Epilepsy care and outcome in low- and middle-income countries: a scoping review. J Neurosci Rural Pract 15(1):8–15. 10.25259/JNRP_527_2023 PMC10927051 38476408

[CR15] Bar J, Samuelov L, Sprecher E, Mashiah J (2019) Griseofulvin vs terbinafine for paediatric tinea capitis: When and for how long. Mycoses 62(10):949–953. 10.1111/myc.12970 31343780

[CR16] Batool R, Qamar ZH, Salam RA et al (2024) Efficacy of typhoid vaccines against culture-confirmed Salmonella Typhi in typhoid endemic countries: a systematic review and meta-analysis. Lancet Global Health 12(4):e589-e598. 10.1016/S2214-109X(23)00606-X 38485426

[CR17] Becerra AF, Amanamba U, Lopen JE, Blaker NJ, Winchester DE (2025) The current use of vasoactive agents in cardiogenic shock related to myocardial infarction and acute decompensated heart failure. Am Heart J Plus Cardiol Res Pract 52:100524. 10.1016/j.ahjo.2025.100524 PMC11960524 40170689

[CR18] Becker LL, Kaindl AM (2023) Corticosteroids in childhood epilepsies: a systematic review. Front Neurol 14:1142253. 10.3389/fneur.2023.1142253 36970534 PMC10036579

[CR19] Benn CS, Aaby P (2023) Measles vaccination and reduced child mortality: Prevention of immune amnesia or beneficial non-specific effects of measles vaccine? J Infect 87(4):295–304. 10.1016/j.jinf.2023.07.010 37482223

[CR20] Bennett PN, Brown MJ, Sharma P (2012) Clinical Pharmacology. 11th edition. Churchilll Livingstone, 2012. ISBN: 978–0–7020–4084–9

[CR21] Benzon HT, Provenzano DA, Nagpal A et al (2024) Use and safety of corticosteroid injections in joints and musculoskeletal soft tissue: guidelines from the American Society of Regional Anesthesia and Pain Medicine, the American Academy of Pain Medicine, the American Society of Interventional Pain Physicians, and the International Pain and Spine Intervention Society. Regional Anesthesia & Pain Medicine, 26 February 2025. 10.1136/rapm-2024-105656 40015722

[CR22] Bernstein DI, Blaiss M, Dellon ES et al (2025) Benefits of epinephrine for anaphylaxis outweigh potential harm—a safety review. J Allergy Clin Immunol Pract. 10.1016/j.jaip.2025.04.018 40254271

[CR23] Bindel LJ, Seifert R (2025a) Determinants of prescribing behaviour of antibacterial drugs in Europe and use of appropriate nomenclature in the literature. Naunyn-Schmiedeberg's Arch Pharmacol. 10.1007/s00210-025-04511-2 PMC12894117 40802007

[CR24] Bindel LJ, Seifert R (2025b) Evidence of lithium underuse in bipolar disorder: analysis of lithium and antipsychotic consumption, prediction of future trends, regional disparities and indicators of rational and inappropriate use in Europe. Naunyn-Schmiedeberg's Arch Pharmacol. 10.1007/s00210-025-04389-0 PMC12678625 40580313

[CR25] Bloom JE, Chan W, Kaye DM, Stub D (2023) State of shock: contemporary vasopressor and inotrope use in cardiogenic shock. JAHA 12(15). 10.1161/JAHA.123.029787 PMC10492962 37489740

[CR26] Bonifaz A, Lumbán-Ramírez P, García-Sotelo RS, Vidaurri de la Cruz H, Toledo-Bahena M, Valencia-Herrera A (2024) Now that griseofulvin is not available, what to do with tinea capitis treatments? Expert Rev Anti Infect Ther 22(12):1017–1022. 10.1080/14787210.2024.2405936 39297581

[CR27] Bortolussi R, Martin S (2024) Antifungal agents for common outpatient paediatric infections. Canadian Paediatric Society. https://cps.ca/en/documents/position/antifungal-agents-common-infections. Last updated 21 Nov 2024. Last accessed 6 Dec 2025

[CR28] Bos JC, van Hest RM, Mistíco MC, Nunguiane G, Lang CN et al (2017) Pharmacokinetics and pharmacodynamic target attainment of benzylpenicillin in an adult severely Ill Sub-Saharan African patient population. Clin Infect Dis 66(8):1261–1269. 10.1093/cid/cix961 29112711

[CR29] Braitberg G (2019) Chapter 98 - Drugs and antidotes in acute intoxication. Crit Care Nephrol, 3rd edition, pp 574–588.e3. 10.1016/B978-0-323-44942-7.00098-4

[CR30] Bremond-Gignac D, Chiambaretta F, Milazzo S (2011) A European perspective on topical ophthalmic antibiotics: current and evolving options. Ophthalmology and Eye Diseases 3:29–43. 10.4137/OED.S4866 23861622 PMC3661455

[CR31] Brendan RJ, Iqbal S, Mahon B (2015) Updated recommendations for the use of typhoid vaccine — advisory committee on immunization practices. Morbidity and Mortality Weekly Report (MMWR) 64(11):305–308. CDC, United States. https://www.cdc.gov/mmwr/preview/mmwrhtml/mm6411a4.htm. Last updated 27 Mar 2015. Last accessed 6 Dec 2025 PMC4584884 25811680

[CR32] Brodie MJ, Kwan P (2012) Current position of phenobarbital in epilepsy and its future. Epilepsia Volume 53(Issues 8, Special Issue: Phenobarbital: The Centenary ‐ 10th European Congress on Epileptology, London ‐ October 1, 2012):Pages 40-46. 10.1111/epi.12027

[CR33] Bundesinstitut für Risikobewertung (BfR) (2014) Toxikologische Bewertung von Chloramphenicol. BfR, Germany. https://www.bfr.bund.de/cm/343/toxikologische-bewertung-von-chloramphenicol.pdf. Last updated 20 Mar 2014. Last accessed 28 Nov 2025

[CR34] Cabrera-Aguas M, Chidi-Egboka N, Kandel H, Watson SL (2024) Antimicrobial resistance in ocular infection: a review. Clin Exp Ophthalmol Volume52(Issue3):Pages 258-275. 10.1111/ceo.14377 38494451

[CR35] Carey RM, Calhoun DA, Bakris GL et al (2018) Resistant hypertension: detection, evaluation, and management: a scientific statement from the American Heart Association. Hypertension 72(5). 10.1161/HYP.0000000000000084 PMC6530990 30354828

[CR36] Castaño BL, Silva AA, Hernandez-Velasco LL, Pinheiro APDS, Gibaldi D, Mineo JR, Silva NM, Lannes-Vieira J (2022) Sulfadiazine Plus Pyrimethamine Therapy Reversed Multiple Behavioral and Neurocognitive Changes in Long-Term Chronic Toxoplasmosis by Reducing Brain Cyst Load and Inflammation-Related Alterations. Front Immunol 13:822567. 10.3389/fimmu.2022.822567 35572567 PMC9091718

[CR37] Centanni M, Duntas L, Feldt-Rasmussen U, Koehrle J, Peeters RP, Razvi S, Trimboli P, Virili C (2025) ETA guidelines for the use of levothyroxine sodium preparations in monotherapy to optimize the treatment of hypothyroidism. European Thyroid Journal 14(4):e250123. 10.1530/ETJ-25-0123 40622204 PMC12323320

[CR38] Chakraborty A, Patel P, Can AS (2024) Calcium Gluconate. StatPearls Publishung, Treasure Island (FL) 32491395

[CR39] Chan YL, Sharkawi DE, Chanouzas D, O'Connor D, et al (2025) British society for haematology updated guidelines for the diagnosis and management of tumour lysis syndrome in adults and children with haematological malignancies: A focus on patient safety. BJHaem, Volume 207, Issue 4, October 2025, Pages 1248–1258. 10.1111/bjh.70092 40903841

[CR40] Chan JJ (2025) Psoriasis: an update on topical and systemic therapies. Aust Prescr 48(3):87–92. 10.18773/austprescr.2025.026 40568686 PMC12187478

[CR41] Chen S, Khusial T, Patel D, Sing S, Yakubova T, Wang A, Nguyen T (2015) Digoxin use in modern medicine. US Pharm. 2015;40(2):44–48. https://www.uspharmacist.com/article/digoxin-use-in-modern-medicine#:~:text=3-,Conclusion,rather%20than%20first%2Dline%20therapy.

[CR42] Chen X, Feng L, Yao H et al (2021) Efficacy and safety of diazoxide for treating hyperinsulinemic hypoglycemia: a systematic review and meta-analysis. PLOS One. 10.1371/journal.pone.0246463 PMC7877589 33571197

[CR43] Chevalier FD, Le Clec’h W, McDew-White M, Menon V, Guzman MA, Holloway SP, Cao X, Taylor AB, Kinung’hi S, Gouvras AN, Webster BL, Webster JP, Emery AM, Rollinson D, Garba Djirmay A, Al Mashikhi KM, Al Yafae S, Idris MA, Moné H, Mouahid G, Anderson TJC (2019) Oxamniquine resistance alleles are widespread in Old World *Schistosoma mansoni* and predate drug deployment. PLoS Pathog 15(10):e1007881. 10.1371/journal.ppat.1007881 31652296 PMC6834289

[CR44] Chin PKL, Doogue MP (2025) Oral anticoagulation for adults with atrial fibrillation or venous thromboembolism. Aust Prescr 48:161–6. 10.18773/austprescr.2025.047 41164099 PMC12566413

[CR45] Ciurtin C, Sprachez M, Elephtheriou D, Broagan P (2025) The evolving landscape of rheumatic drugs and their indications in paediatric and adolescent care. Rheumatology (Oxford) 64:6048–6070. 10.1093/rheumatology/keaf455 40845184 PMC12671880

[CR46] Cohen KA, Manson AL, Earl AM, Pym AS (2020) Evidence for expanding the role of streptomycin in the management of drug-resistant *Mycobacterium tuberculosis*. Antimicrob Agents Chemother 64:10.1128/aac.00860-20. 10.1128/aac.00860-20 PMC7449167 32540971

[CR47] Colucci P, Yue CS, Ducharme M, Benvenga S (2013) A review of the pharmacokinetics of levothyroxine for the treatment of hypothyroidism. European endocrinology 9(1):40–47. 10.17925/EE.2013.09.01.40 30349610 PMC6193522

[CR48] Cortes J, Moore JO, Maziarz RT, Wetzler M, Craig M, Matous J, Luger S, Dey BR, Schiller GJ, Pham D, Abboud CN, Krishnamurthy M, Brown A Jr, Laadem A, Seiter K (2010) Control of plasma uric acid in adults at risk for tumor lysis syndrome: efficacy and safety of rasburicase alone and rasburicase followed by allopurinol compared with allopurinol alone- -results of a multicenter phase III study. J Clin Oncol 28(27):4207–4213. 10.1200/JCO.2009.26.8896 20713865 PMC4979236

[CR49] Cramer JP, López-Vélez R, Burchard GD et al (2011) Treatment of imported severe malaria with artesunate instead of quinine - more evidence needed?. Malar J 10:256. 10.1186/1475-2875-10-256 PMC3224352 21899729

[CR50] Crane AD, Militello M, Faulx MD (2024) Digoxin is still useful, but is still causing toxicity. Cleve Clin J Med 91(8):489–499. 10.3949/ccjm.91a.23105 39089856

[CR51] Danso-Appiah A, Olliaro PL, Donegan S, Sinclair D, Utzinger J (2022) Drugs for treating *Schistosoma mansoni* infection. Cochrane Database Syst Rev 3:CD000528. 10.1002/14651858.CD000528.pub2 PMC6532716 23450530

[CR52] Das A, Panda S (2017) Use of Topical Corticosteroids in Dermatology: An Evidence-based Approach. Indian J Dermatol 62(3):237–250. 10.4103/ijd.IJD_169_17 28584365 PMC5448257

[CR53] Dattani S (2025) How effective and safe are measles vaccines? Our World in Data. https://ourworldindata.org/measles-vaccine-effectiveness-safety. Last updated 21 Apr 2025. Last accessed 6 Dec 2025

[CR54] Dattani S, Spooner F (2025) Measles vaccines save millions of lives each year. Our World in Data. https://ourworldindata.org/measles-vaccines-save-lives. Last updated 19 May 2025. Last accessed 6 Dec 2025

[CR55] Dawn L, Whited L (2023) Dimercaprol. StatPearls, Treasure Island (FL) 31747211

[CR56] Dhyani P, Quispe C, Sharma E et al (2022) Anticancer potential of alkaloids: a key emphasis to colchicine, vinblastine, vincristine, vindesine, vinorelbine and vincamine. Cancer Cell Int 22:206. 10.1186/s12935-022-02624-9 35655306 PMC9161525

[CR57] DocCheck (2025) Griseofulvin. https://flexikon.doccheck.com/de/Griseofulvin. Last updated 23 Apr 2025. Last accessed 29 Nov 2025

[CR58] Duff P (2015) Is azithromycin a good alternative to erythromycin for PPROM prophylaxis?. OBG Management 27(5). https://www.mdedge.com/obgyn/article/99948/obstetrics/azithromycin-good-alternative-erythromycin-pprom-prophylaxis. Last updated 27 May 2015. Last accessed 29 Nov 2025

[CR59] Dunay IR, Gajurel K, Dhakal R, Liesenfeld O, Montoya JG (2018) Treatment of toxoplasmosis: historical perspective, animal models, and current clinical practice. Clin Microbiol Rev 31. 10.1128/cmr.00057-17 PMC6148195 30209035

[CR60] Electronic medicines compendium (EMC) (2022) Chloramphenicol 1.0% w/w Antibiotic Eye Ointment. EMC, UK. https://www.medicines.org.uk/emc/product/9770/smpc#gref. Last updated: March 29 2022. Accessed 28 Nov 2025

[CR61] Electronic medicines compendium (EMC) (2022) Chloramphenicol 1.0% w/w Antibiotic Eye Ointment. EMC, UK. https://www.medicines.org.uk/emc/product/9770/smpc#gref. Last updated 29 Mar 2022. Last accessed 28 Nov 2025

[CR62] Elmets CA, Korman NJ, Prater EF et al (2021) Joint AAD–NPF Guidelines of care for the management and treatment of psoriasis with topical therapy and alternative medicine modalities for psoriasis severity measures. JAAD 84(2):432–470. 10.1016/j.jaad.2020.07.087 32738429

[CR63] European Medicines Agency (EMA) (2020) Guideline on the quality of water for pharmaceutical use. GHMP, CVMP, EMA. https://www.ema.europa.eu/en/documents/scientific-guideline/guideline-quality-water-pharmaceutical-use_en.pdf. Last updated 20 July 2020. Last accessed 24 Nov 2025

[CR64] European Monitoring Centre for Drugs and Drug Addiction (EMCDDA) (2015) Preventing fatal overdoses: a systematic review of the effectiveness of take-home naloxone. EMCDDA Papers, Publications Office of the European Union, Luxembourg. 10.2810/396726

[CR65] Esposito A, Vigone MC, Polizzi M, Wasniewska MG, Cassio A, Mussa A, Gastaldi R, Di Mase R, Vincenzi G, Pozzi C, Peroni E, Bravaccio C, Capalbo D, Bruzzese D, Salerno M (2022) Effect of initial levothyroxine dose on neurodevelopmental and growth outcomes in children with congenital hypothyroidism. Front Endocrinol (Lausanne) 13:923448. 10.3389/fendo.2022.923448 36133316 PMC9484273

[CR66] Faggiano A, Mazzilli R, Natalicchio A, Adinolfi V et al (2022) Corticosteroids in oncology: use, overuse, indications, contraindications. An Italian Association of Medical Oncology (AIOM)/ Italian Association of Medical Diabetologists (AMD)/ Italian Society of Endocrinology (SIE)/ Italian Society of Pharmacology (SIF) multidisciplinary consensus position paper. Crit Rev Oncol/Hematol 180:103826. 10.1016/j.critrevonc.2022.103826 36191821

[CR67] Fiorda Diaz J, Echeverria-Villalobos M, Esparza Gutierrez A, Dada O, Stoicea N, Ackermann W, Abdel-Rasoul M, Heard J, Uribe A, Bergese UAa (2022) Sugammadex versus neostigmine for neuromuscular blockade reversal in outpatient surgeries: a randomized controlled trial to evaluate efficacy and associated healthcare cost in an academic center. Front Med 9:1072711, 1072711. 10.3389/fmed.2022.1072711 PMC9772266 36569123

[CR68] Folk A, Cotoraci C, Balta C, Suciu M, Herman H, Boldura OM, Dinescu S, Paiusan L, Ardelean A, Hermenean A (2016) Evaluation of Hepatotoxicity with Treatment Doses of Flucytosine and Amphotericin B for Invasive Fungal Infections. Biomed Res Int. 10.1155/2016/5398730 26949702 PMC4754496

[CR69] Food and Drug Adminstration (FDA) (2014) Potassium chloride label. FDA. Reference ID: 3677449. https://www.accessdata.fda.gov/drugsatfda_docs/label/2014/206814lbl.pdf. Last updated Dec 2014. Last accessed 20 Nov 2025

[CR70] Food and Drug Adminstration (FDA) (2023) Expanded access to clofazimine. Center for Drug Evaluation and Research (CDER). https://www.fda.gov/about-fda/center-drug-evaluation-and-research-cder/expanded-access-clofazimine. Last updated 15 Nov 2023. Last accessed 28 Nov 2025

[CR71] Friedman B, Cronstein B (2019) Methotrexate mechanism in treatment of rheumatoid arthritis. Jt Bone Spine 86(3):301–307. 10.1016/j.jbspin.2018.07.004 PMC6360124 30081197

[CR72] Fuchs A, Bielicki J, Mathur S, Sharland M, Van Den Anker JN (2018) Reviewing the WHO guidelines for antibiotic use for sepsis in neonates and children. Paediatr Int Child Health 38(sup1):S3–S15. 10.1080/20469047.2017.1408738 29790842 PMC6176768

[CR73] Gamil Y, Hamed MG, Elsayed M, Essawy A, Medhat S, Zayed SO, Ismail RM (2024) The anti-fungal effect of miconazole and miconazole-loaded chitosan nanoparticles gels in diabetic patients with oral candidiasis-randomized control clinical trial and microbiological analysis. BMC Oral Health 24(1):196. 10.1186/s12903-024-03952-0 38321454 PMC10848391

[CR74] Garcia A, Aguiar C (2025) Finding the right dose of acetylsalicylic acid for secondary prevention of atherosclerotic cardiovascular disease. Rev Port Cardiol 44(7):423–426. 10.1016/j.repc.2025.06.002 40617624

[CR75] Gephart BD, Coulter DW, Solheim JC (2025) Effects of the alkylating agent cyclophosphamide in potentiating anti-tumor immunity. Int J Mol Sci 26(13):6440. 10.3390/ijms26136440 40650216 PMC12249703

[CR76] Gerhardsson L, Aasseth J (2016) Chapter 7 - Guidance for clinical treatment of metal poisonings—use and misuse of chelating agents. Chelation Therapy in the Treatment of Metal Intoxication, pp 313–341. 10.1016/B978-0-12-803072-1.00007-9

[CR77] Ghinea N (2024) The increasing costs of medicines and their implications for patients, physicians and the health system. Intern Med J 54(4):545–550. 10.1111/imj.16370 38572698

[CR78] Giolito A, Levy C, Varon E, Cohen R, Hanna S, Assad Z, Lenglart L, Bechet S, Bonacorsi S, Dubos F, Launay E, Pelleter M, Rybak A, Angoulvant F, Levy M, Ouldali N, French Paediatric Meningitis Network (2025) Adjunctive dexamethasone and 30-day all-cause death after hospital admission in paediatric pneumococcal meningitis: a propensity score analysis. Lancet Child Adolesc Health 9(4):255–261. 10.1016/S2352-4642(25)00029-X 40113367

[CR79] Gisondi P, Gracia-Cazaña T, Kurzen H, Galván J (2024) Calcipotriol/betamethasone dipropionate for the treatment of psoriasis: mechanism of action and evidence of efficacy and safety versus topical corticosteroids. J Clin Med 13(15):4484. 10.3390/jcm13154484 39124750 PMC11313259

[CR80] Global Initiative For Asthma (GINA) (2025) 2025 Global Strategy for Asthma Management and Prevention (2025 update). https://ginasthma.org/wp-content/uploads/2025/11/GINA-2025-Update-25_11_08-WMS.pdf. Last updated Nov 2025. Last accessed 7 Dec 2025

[CR81] Global Initiative for Chronic Obstructive Lung Disease (GOLD) (2025) Global stratety for the diagnosis, management, and prevention of chronic obstructive pulmonary disease (2025 report). GOLD, Inc. https://goldcopd.org/wp-content/uploads/2024/11/GOLD-2025-Report-v1.0-15Nov2024_WMV.pdf. Last updated 2025. Last accessed 7 Dec 2025

[CR82] Gloeck NR, Leong TD, Mthethwa M, Iwu-Jaja CJ, Katoto PD, Wiysonge CS, Kredo T (2025) Typhoid conjugate vaccines for preventing typhoid fever (enteric fever). Cochrane Database Syst Rev 5(5):CD015746. 10.1002/14651858.CD015746.pub2 40326553 PMC12053466

[CR83] GOV UK (2017) Miconazole (Daktarin): over-the-counter oral gel contraindicated in patients taking warfarin. Drug Safety Updated. https://www.gov.uk/drug-safety-update/miconazole-daktarin-over-the-counter-oral-gel-contraindicated-in-patients-taking-warfarin#:~:text=While%20further%20measures%20were%20being,the%20label%20(tube%20and%20carton. Last updated 26 Sept 2017. Last accessed 6 Dec 2025

[CR84] Government of Canada (2025a) List of drugs for an urgent public health need. Health Canada. https://www.canada.ca/en/health-canada/services/drugs-health-products/access-drugs-exceptional-circumstances/list-drugs-urgent-public-health-need.html. Last updated 04 July 2025. Last accessed 30 Nov 2025

[CR85] Government of Canada (2025b) Measles vaccines: Canadian Immunization Guide. https://www.canada.ca/en/public-health/services/publications/healthy-living/canadian-immunization-guide-part-4-active-vaccines/page-12-measles-vaccine.html. Last updated June 2025. Last accessed 6 Dec 2025

[CR86] Greenwood D, Irving WL (2012) 5 - Antimicrobial agents. Medical Microbiology (Eighteenth Edition). Churchill Livingstone 2012, pp 54–68. 10.1016/B978-0-7020-4089-4.00020-2

[CR87] Griffin MO, Fricovsky E, Ceballos G, Villarreal F (2010) Tetracyclines: a pleitropic family of compounds with promising therapeutic properties. Review of the literature. American journal of physiology. Cell physiology 299(3):C539–C548. 10.1152/ajpcell.00047.2010 20592239 PMC2944325

[CR88] Güler S, Könemann H, Wolfes J, Güner F, Ellermann C, Rath B, Frommeyer G, Lange PS, Köbe J, Reinke F, Eckardt L (2023) Lidocaine as an anti-arrhythmic drug: Are there any indications left? Clin Transl Sci 16(12):2429–2437. 10.1111/cts.13650 37781966 PMC10719458

[CR89] Guo Y, Yang J, Wang H, Sha W, Yu F (2025) Key resistance-associated mutations in multidrug-resistant tuberculosis: a genomic study from Shanghai, China, with a focus on aminoglycosides. BMC Microbiol 25(1):702. 10.1186/s12866-025-04446-x 41168684 PMC12574157

[CR90] Haigh KA, Twabi HH, Boloko L, Namale PE, Lutje V, Nevitt S et al (2024) Efficacy and safety of higher dose rifampicin in adults with presumed drug-susceptible tuberculosis: an updated systematic review and meta-analysis. eClinicalMedicine 77:102857. 10.1016/j.eclinm.2024.102857 PMC11474450 39416385

[CR91] Haney S, Hancox RJ (2005) Rapid onset of tolerance to beta-agonist bronchodilation. Respiratory Medicine, Volume 99, Issue 5, p566–571, May 2005. 10.1016/j.rmed.2004.10.014 15823453

[CR92] Hamed KM, Dighriri IM, Baomar AF, Alharthy BT, Alenazi FE, Alali GH, Alenazy RH, Alhumaidi NT, Alhulayfi DH, Alotaibi YB, Alhumaidan SS, Alhaddad ZA, Humadi AA, Alzahrani SA, Alobaid RH (2022) Overview of Methotrexate Toxicity: A Comprehensive Literature Review. Cureus 14(9):e29518. 10.7759/cureus.29518 36312688 PMC9595261

[CR93] Harsanyi A, Conta A, Pichon L, Rabion A, Grenier S, Sandford G (2017) One-step continuous flow synthesis of antifungal WHO essential medicine flucytosine using fluorine. OPR&D 21(2):273–276. 10.1021/acs.oprd.6b00420

[CR94] Hartmann RJ, Boyle J, Peterson K (2025) Activated charcoal in the poisoned patient: beyond the one-hour mark. Can J Emerg Med 27:322–323. 10.1007/s43678-025-00916-3 40354025

[CR95] Hasson F, Keeney S, McKenna H (2025) Revisiting the Delphi technique - research thinking and practice: a discussion paper. Int J Nurs Stud 168:105119. 10.1016/j.ijnurstu.2025.105119 40383005

[CR96] Havlickova B, Czaika VA, Friedrich M (2008) Epidemiological trends in skin mycoses worldwide. Mycoses 51(s4):2–15. 10.1111/j.1439-0507.2008.01606.x 18783559

[CR97] Heidenreich PA, Bozkurt B, Aguilar D et al (2022) 2022 AHA/ACC/HFSA guideline for the management of heart failure: a report of the American College of Cardiology/American Heart Association Joint Committee on Clinical Practice Guidelines. Circulation 145(18). 10.1161/CIR.0000000000001063 35363499

[CR98] Heinz E (2018) The return of Pfeiffer’s bacillus: rising incidence of ampicillin resistance in *Haemophilus influenzae*. Microbiol Genom 4(9):e000214. 10.1099/mgen.0.000214 PMC6202453 30207515

[CR99] Hennessy G, Brewster M, Mund P et al (2024) Naloxone Is the preferred medication for decreasing opioid overdose fatalities. Psychiatric News 59(11). 10.1176/appi.pn.2024.11.11.11

[CR100] Hernández Ceruelos A, Romero-Quezada LC, Ruvalcaba Ledezma JC, López Contreras L (2019) Therapeutic uses of metronidazole and its side effects: an update. Eur Rev Med Pharmacol Sci 23(1):397–401. 10.26355/eurrev_201901_16788 30657582

[CR101] Ho KM, Power BM (2010) Benefits and risks of furosemide in acute kidney injury. Anaesthesia 65(3):283–293. 10.1111/j.1365-2044.2009.06228.x 20085566

[CR102] Holmes NE, Charles PGP (2009) Safety and Efficacy Review of Doxycycline. Clinical Medicine Therapeutics 2009:1. 10.4137/CMT.S2035

[CR103] Hosoya Y, Yamamoto M, Hanada H, Yamamoto T et al (2025) Comparative efficacy of noradrenaline vs. other vasopressors on outcomes in patients with cardiogenic shock ― a systematic review and meta-analysis. Circ Rep. 10.1253/circrep.CR-25-0188 PMC12688426 41377633

[CR104] Hristovska AM, Duch P, Allingstrup M, Afshari A (2017) Efficacy and safety of sugammadex versus neostigmine in reversing neuromuscular blockade in adults. Cochrane Database Syst Rev 8:CD012763. 10.1002/14651858.CD012763 28806470 PMC6483345

[CR105] Hsueh PR (2005) Decreasing rates of resistance to penicillin, but not erythromycin, in *Streptococcus pneumoniae* after introduction of a policy to restrict antibiotic usage in Taiwan. Clin Microbiol Infect 11(11):925–927. 10.1111/j.1469-0691.2005.01245.x 16216110

[CR106] Ichai C, Orban JC, Fontaine E (2014) Sodium lactate for fluid resuscitation: the preferred solution for the coming decades?. Crit Care 18:163. 10.1186/cc13973 PMC4095570 25043707

[CR107] Infectious Diseases Society of America (2025) Guidance on the treatment of antimicrobial resistant gram-negative infections. https://www.guidelinecentral.com/guideline/308215/#section-3924335. Last updated 18 Oct 2025. Last accessed 25 Nov 2025

[CR108] International League Against Epilepsy (ILAE) (2020) Second-line therapy for status epilepticus: What’s the latest? Epigraph 22(2), Spring 2020. https://www.ilae.org/journals/epigraph/epigraph-vol-22-issue-2-spring-2020/second-line-therapy-for-status-epilepticus-what-s-the-latest#:~:text=Phenytoin%20is%20a%20second%2Dline%20medication%20used%20to,Rates%20of%20life%2Dthreatening%20hypotension%2C%20arrhythmia%2C%20or%20death. Last updated 2020. Last accessed 4 Dec 2025

[CR109] Isbister JP, Fisher M (1980) Adverse effects of plasma volume expanders. Anaesth Intensive Care 8(2):145–151. 10.1177/0310057X8000800208 6156607

[CR110] Jabri A, Alhuneafat L, Shahrori Z, Hamade H, Nasser F, Rayyan A, Mhanna M, Al Abdouh A, Haddadin F, Balakumaran K (2022) Impact of digoxin use on guideline-directed medical therapy in patients with heart failure with reduced ejection fraction. J Clin Med Res 14(8):315–320. 10.14740/jocmr4772 36128010 PMC9451555

[CR111] Jacob S, Farrugia ME, Hewamadduma C et al (2025) Association of British Neurologists (ABN) autoimmune myasthenia gravis management guidelines (2025 update). Pract Neurol 25:422–437. 10.1136/pn-2025-004655 40940171

[CR112] Kahaly GJ, Gottwald-Hostalek U (2022) Use of levothyroxine in the management of hypothyroidism: A historical perspective. Front Endocrinol 13:1054983. 10.3389/fendo.2022.1054983 PMC9666762 36407302

[CR113] Kashyap PV, Chabri S (2023) Steroids in headache: a comprehensive review of recent research. Ann Neurosci 30(4):256–261. 10.1177/09727531231173286 38020407 PMC10662276

[CR114] Kaushik D, Mohan M, Borade DM, Swami OC (2014) Ampicillin: rise fall and resurgence. J Clin Diagn Res 8(5):ME01-ME3. 10.7860/JCDR/2014/8777.4356 24995206 PMC4080027

[CR115] Kciuk M, Gielecińska A, Mujwar S, Kołat D, Kałuzińska-Kołat Ż, Celik I, Kontek R (2023) Doxorubicin-An Agent with Multiple Mechanisms of Anticancer Activity. Cells 12(4):659. 10.3390/cells12040659 36831326 PMC9954613

[CR116] Kehl S, Hösli I, Pecks U, Reif P, Schild RL, Schmidt M, Schmitz D, Schwarz C, Surbek D, Abou-Dakn M (2021) Induction of labour. Guideline of the DGGG, OEGGG and SGGG (S2k, AWMF registry no. 015-088, December 2020). Geburtshilfe Frauenheilkd 81(8):870–895. 10.1055/a-1519-7713 34393254 PMC8354342

[CR117] Khan MI, Franco-Paredes C, Sahastrabuddhe S, Ochiai RL, Mogasale V, Gessner BD (2017) Barriers to typhoid fever vaccine access in endemic countries. Res Rep Top Med, 8;37–44. 10.2147/RRTM.S97309 PMC6034652 30050343

[CR118] Kidney Disease Improving Global Outcomes CKD Work Group (KDIGO) (2024) KDIGO 2024 Clinical practice guideline for the evaluation and management of chronic kidney disease. Kidney Int 105(4)S117-S314. 10.1016/j.kint.2023.10.018 38490803

[CR119] Kim SJ, Toma HS (2011) Antimicrobial resistance and ophthalmic antibiotics: 1-year results of a longitudinal controlled study of patients undergoing intravitreal injections. Arch Ophthalmol 129(9):1180–1188. 10.1001/archophthalmol.2011.213 21911665

[CR120] Kim DH, Chung HJ, Bleys J, Ghohestani RF (2009) Is paromomycin an effective and safe treatment against cutaneous leishmaniasis? A meta-analysis of 14 randomized controlled trials. PLoS Negl Trop Dis 3(2):e381. 10.1371/journal.pntd.0000381 19221595 PMC2637543

[CR121] Kim HJ, Lee YJ, Song MJ et al (2023a) Real-world experience of adverse reactions-necessitated rifampicin-sparing treatment for drug-susceptible pulmonary tuberculosis. Sci Rep 13:11275. 10.1038/s41598-023-38394-1 37438379 PMC10338469

[CR122] Kim JH, Jeong H, Choo YH, Kim M, Ha EJ, Oh J, Shim Y, Kim SB, Jung HG, Park SH, Kim JO, Kim J, Kim HS, Lee S (2023b) Optimizing mannitol use in managing increased intracranial pressure: a comprehensive review of recent research and clinical experiences. Korean J Neurotrauma 19(2):162–176. 10.13004/kjnt.2023.19.e25 PMC10329884 37431377

[CR123] Kinsella SM, Carvalho B, Dyer RA et al (2017) International consensus statement on the management of hypotension with vasopressors during caesarean section under spinal anaesthesia. Anaesthesia 2018(73):3–6. 10.1111/anae.14080 29090733

[CR124] Kitai T, Kohsaka S, Kato T et al (2025) JCS/JHFS 2025 Guideline on diagnosis and treatment of heart failure. JCF 31(8):1164–1322. 10.1016/j.cardfail.2025.02.014 40155256

[CR125] Konstantinovic N, Guegan H, Stäjner T, Belaz S, Robert-Gangneux F (2019) Treatment of toxoplasmosis: Current options and future perspectives. Food and Waterborne Parasitology 15:e00036. 10.1016/j.fawpar.2019.e00036 32095610 PMC7033996

[CR126] Kosmas CE, Silverio D, Sourlas A, Montan PD, Guzman E (2018) Role of spironolactone in the treatment of heart failure with preserved ejection fraction. Ann Transl Med 6(23):461. 10.21037/atm.2018.11.16 PMC6312809 30603649

[CR127] Kosmas CE, Silverio D, Sourlas A, Montan PD, Guzman E (2018) Role of spironolactone in the treatment of heart failure with preserved ejection fraction. Annals of translational medicine, 6(23), 461. 10.21037/atm.2018.11.16 PMC6312809 30603649

[CR128] Koźmiński P, Halik PK, Chesori R, Gniazdowska E (2020) Overview of dual-acting drug methotrexate in different neurological diseases, autoimmune pathologies and cancers. Int J Mol Sci 21(10):3483. 10.3390/ijms21103483 32423175 PMC7279024

[CR129] Kumbhani D, Cibotti-Sun M, Moore M (2025) 2025 Acute coronary syndromes guideline-at-a-glance. JACC 85(22):2128–2134. 10.1016/j.jacc.2025.01.018 40013745

[CR130] La Torre F, Meliota G, Civino A, Campanozzi A, Cecinati V, Rosati E, Sacco E, Santoro N, Vairo U, Cardinale F (2025) Advancing multidisciplinary management of pediatric hyperinflammatory disorders. Front Pediatr 13:1553861. 10.3389/fped.2025.1553861 40370972 PMC12075326

[CR131] Laing R, Waning B, Gray A, Ford N, Hoen E (2003) 25 years of the WHO essential medicines lists: progress and challenges. Lancet 361(9370):1723–1729. 10.1016/S0140-6736(03)13375-2 12767751

[CR132] LaPlante KL, Dhand A, Wright K, Lauterio M (2022) Re-establishing the utility of tetracycline-class antibiotics for current challenges with antibiotic resistance. Ann Med 54(1):1686–1700. 10.1080/07853890.2022.2085881 35723082 PMC9225766

[CR133] Li X, Balas M, Mathew DJ (2025) A review of ocular and systemic side effects in glaucoma pharmacotherapy. J Clin Transl Ophthalmol 3(1):2. 10.3390/jcto3010002

[CR134] Li X, Li G, Yang J, Jin G, Shao Y, Li Y, Wei P, Zhang L (2022) Drug resistance (dapsone, rifampicin, ofloxacin) and resistance-related gene mutation features in leprosy patients: a systematic review and meta-analysis. Int J Mol Sci 23(20):12443. 10.3390/ijms232012443 36293307 PMC9604410

[CR135] Lindner AK, Lejon V, Barrett MP, Blumberg L, Bukachi SA et al (2025) New WHO guidelines for treating rhodesiense human African trypanosomiasis: expanded indications for fexinidazole and pentamidine. Lancet Infect Dis 25(2):e77-e85. 10.1016/S1473-3099(24)00581-4 39389073

[CR136] Liyanage CK, Galappatthy P, Seneviratne SL (2017) Corticosteroids in management of anaphylaxis; a systematic review of evidence. Eur Ann Allergy Clin Immunol 49(5):196–207. 10.23822/EurAnnACI.1764-1489.15 28884986

[CR137] Long N (2020) Dimercaprol. Life in the fastlane (LITFL). https://litfl.com/dimercaprol/. Last updated 03 Nov 2020. Last accessed 24 Nov 2025

[CR138] Longley D, Harkin D, Johnston PG (2003) 5-Fluorouracil: mechanisms of action and clinical strategies. Nat Rev Cancer 338:330–338. 10.1038/nrc1074 12724731

[CR139] Ludwig L, Seifert R (2025) The decline in the clinical relevance of pilocarpine and physostigmine monitored in pharmacology textbooks from 1878 to 2023: nine take-home messages for future (pharmacology) textbook authors. Naunyn Schmiedebergs Arch Pharmacol 5193:5171–5193. 10.1007/s00210-024-03558-x PMC11985697 39531042

[CR140] Lüsebrink E, Binzenhöfer L, Adamo M et al (2024) Cardiogenic shock. Lancet 404(10466):2006–2020. 10.1016/S0140-6736(24)01818-X 39550175

[CR141] Ma LH, Jia L, Bai L (2023) Safety outcomes of salbutamol: A systematic review and meta-analysis. The clinical respiratory journal, 17(12), 1254–1264. 10.1111/crj.13711 PMC10730473 37844914

[CR142] Ma Y, Cen W, Duan M et al (2025) Patients knowledge attitudes and practices regarding superficial fungal infections suggest public health and patient education are warranted. Sci Rep 15112:15112. 10.1038/s41598-025-98919-8 PMC12041239 40301445

[CR143] Magdy JS, McVeigh J, Indraratna P (2022) Diuretics in the management of chronic heart failure: when and how. Aust Prescr 45(6):200–204. 10.18773/austprescr.2022.069 36479331 PMC9722345

[CR144] Marques L, and Vale N (2022) Salbutamol in the management of asthma: A review. International journal of molecular sciences, 23(22), 14207. 10.3390/ijms232214207 PMC9696300 36430683

[CR145] Martin GS (2019) Optimal fluid management in sepsis. Qatar Med J 2019:40. 10.5339/qmj.2019.qccc.40

[CR146] Martinez P, Sabatier JM (2025) Rethinking corticosteroids use in oncology. Front Pharmacol 16:1551111. 10.3389/fphar.2025.1551111 40206059 PMC11979161

[CR147] Marum D, Manning L, Raby E (2021) Revisiting the inoculum effect for *Streptococcus pyogenes* with a hollow fibre infection model. Eur J Clin Microbiol Infect Dis 40(10):2137–2144. 10.1007/s10096-021-04262-x 33948751

[CR148] Matos APS, Viçosa AL, Ré MI, Ricci-Júnior E (2020) A review of current treatments strategies based on paromomycin for leishmaniasis. J Drug Deliv Sci Technol 57:101664. 10.1016/j.jddst.2020.101664

[CR149] Mayser P, Niehaus M (2023) Onychomycosis guideline update. EFSM 3:230001, 230001. 10.52778/efsm.23.0001

[CR150] Mech C, Seifert R (2025) The change in the pharmacological significance of dihydralazine (Germany) and hydralazine (USA). Naunyn Schmiedebergs Arch Pharmacol. 10.1007/s00210-025-04370-x 40632154 PMC12894129

[CR151] Medecins sans frontieres (MSF) (2025c) Amodiaquine = AQ oral. Essential drugs. MSF. https://medicalguidelines.msf.org/en/node/1022. Last updated 2025. Last accessed 25 Nov 2025

[CR152] Medecins sans frontieres (MSF) (2025e) Tetracycline, eye ointment. https://medicalguidelines.msf.org/en/node/1019. Last updated 2025. Last accessed 30 Nov 2025

[CR153] Medecins Sans Frontieres (MSF) (2025f) Dexamethasone oral. MSF medical guidelines. https://medicalguidelines.msf.org/en/node/1684. Last updated 2025. Last accessed 6 Dec 2025

[CR154] Medicines and Healthcare products Regulatory Agency (2023) Calcium chloride, calcium gluconate: potential risk of underdosing with calcium gluconate in severe hyperkalaemia. Drug Safety Update, Government UK. https://www.gov.uk/drug-safety-update/calcium-chloride-calcium-gluconate-potential-risk-of-underdosing-with-calcium-gluconate-in-severe-hyperkalaemia. Last updated 27 June 2023. Last accessed 7 Dec 2025

[CR155] Mehndiratta MM, Pandey S, Kuntzer T (2014) Acetylcholinesterase inhibitor treatment for myasthenia gravis. Cochrane Database Syst Rev (10):CD006986. 10.1002/14651858.CD006986.pub3 PMC7390275 25310725

[CR156] Melbourne Sexual Health Centre (MSHC) (2021) Syphilis treatment guidelines. MSHC, Canada. https://www.mshc.org.au/health-professionals/treatment-guidelines/syphilis-treatment-guidelines. Last updated 21 July 2021. Last updated 29 Nov 2025

[CR157] Menzies NA, Allwood BW, Dean AS, Dodd PJ, Houben RMGJ, James LP, Knight GM, Meghji J, Nguyen LN, Rachow A, Schumacher SG, Mirzayev F, Cohen T (2023) Global burden of disease due to rifampicin-resistant tuberculosis: a mathematical modeling analysis. Nat Commun 14(1):6182. 10.1038/s41467-023-41937-9 37794037 PMC10550952

[CR158] Miao KH, Guthmiller KB (2025) Dextran. Statpearls Publishing, Treasure Island (FL) 32491563

[CR159] Michalik M, Lorenc T, Marcinkowski K, Muras M, Mikszta N, Mikszta J, Kantor K, Marcinkowska J (2025) Advances and prospects for treatment strategies of drug-resistant tuberculosis: a review. GMS Hyg Infect Control 20:Doc33. 10.3205/dgkh000562 40657634 PMC12248249

[CR160] Mijaljica D, Spada F, Harrison IP (2022) Emerging Trends in the Use of Topical Antifungal-Corticosteroid Combinations. Journal of Fungi 8(8):812. 10.3390/jof8080812 36012800 PMC9409645

[CR161] Miller NE, Rushlow D, Stacey SK (2023) Diagnosis and management of sodium disorders: hyponatremia and hypernatremia. Am Fam Physician 108(5):476–486. https://www.aafp.org/pubs/afp/issues/2023/1100/sodium-disorders-hyponatremia-hypernatremia.pdf 37983699

[CR162] Ministry of Health and Family Welfare (2025) NAtionaL Guidelines for Management of Drug Resistant TB. National TB Elimination Programme. Government of India, Central TB Division. https://tbcindia.mohfw.gov.in/wp-content/uploads/2025/01/National-Guidelines-for-Management-of-DR-TB_Final.pdf#:~:text=First%2D%20line%20TB%20drugs%20used%20to%20treat,or%20there%20is%20confirmed%20resistance%20to%20it. Last updated 13 Jan 2025. Last accessed 30 Nov 2025

[CR163] Mo Y, Wang C, Yin D, Li F (2024) Efficacy of Dexamethasone in Reducing Pain and Inflammation and Accelerating Total Hip Arthroplasty Postoperative Recovery: A Randomized Controlled Trial. Journal of Perianesthesia Nursing : Official Journal of the American Society of PeriAnesthesia Nurses 39(4):589–595. 10.1016/j.jopan.2023.10.022 38219078

[CR164] Moffa M, Brook I (2015) Tetracyclines, glycylcyclines, and chloramphenicol. Mandell, Douglas, and Bennett's Principles and Practice of Infectious Diseases. 8th edition 1(chapter 26):322–338.e6. 10.1016/B978-1-4557-4801-3.00026-6

[CR165] Möhrenschlager M, Korting HC, Seidl HP, Ring J, Abeck D (2002) Tinea capitis. Therapeutic options in the post-griseofulvin era. Hautarzt 53(Issue 12):788–794. 10.1007/s00105-002-0365-5 12444518

[CR166] Montinari MR, Minelli S, De Caterina R (2019) The first 3500 years of aspirin history from its roots - a concise summary. Vasc Pharmacol 113:1–8. 10.1016/j.vph.2018.10.008 30391545

[CR167] Morgan SG, Bathula HS, Moon S (2020) Pricing of pharmaceuticals is becoming a major challenge for health systems. BMJ 368:l4627. 10.1136/bmj.l4627 31932289

[CR168] Mückter H, Lieb B, Reich F-X, Hunder G, Walther U, Fichtl B (1997) Are we ready to replace dimercaprol (BAL) as an arsenic antidote? Hum Exp Toxicol 16(8):460–465. 10.1177/096032719701600807 9292286

[CR169] Munos MK, Walker CL, Black RE (2010) The effect of oral rehydration solution and recommended home fluids on diarrhoea mortality. Int J Epidemiol 39(1):i75–i87. 10.1093/ije/dyq025 20348131 PMC2845864

[CR170] Münzel T, Daiber A, Mülsch A (2005) Explaining the phenomenon of nitrate tolerance. Circ Res. 10.1161/01.RES.0000184694.03262.6d 16195486

[CR171] National Health Service (NHSa) (2022) Side effects of erythromycin. NHS, UK. https://www.nhs.uk/medicines/erythromycin/side-effects-of-erythromycin/. Last updated 4 Feb 2022. Last accessed 29 Nov 2025

[CR172] National Health Service (NHS) (2025b) Dexamethasone. Medicines information.NHS Scotland. https://www.rightdecisions.scot.nhs.uk/scottish-palliative-care-guidelines/medicines-information/dexamethasone/. Last updated 2025. Last accessed 6 Dec 2025

[CR173] National Institute for Health and Care Excellence (NICE) (2016a) Stable angina: management. Clinical guideline. Reference number: CG126. https://www.nice.org.uk/guidance/cg126/chapter/Recommendations. Last updated 25 Aug 2016. Last accessed 1 Dec 2025

[CR174] National Institute for Health and Care Excellence (NICE) (2016b) Palliative care for adults: strong opioids for pain relief. Clinical guideline. Reference number: CG140. https://www.nice.org.uk/guidance/cg140/chapter/recommendations. Last updated 3 Aug 2016. Last accessed 3 Dec 2025

[CR175] National Institute for Health and Care Excellence (NICE) (2021) Clostridioides difficile infection: antimicrobial prescribing. NICE guideline, 2021. NG 199. https://www.nice.org.uk/guidance/ng199/chapter/Rationale-and-impact. Last updated 23 July 2021. Last accessed 29 Nov 2025 34464040

[CR176] National Institute for Health and Care Excellence (NICE). (2022a). Glaucoma: diagnosis and management. NICE guideline. Reference number: NG81. Last updated 26 Jan 2022. Last accessed 7 Dec 2025. https://www.nice.org.uk/guidance/ng81

[CR177] National Institute for Health and Care Excellence (NICE) (2022b) Gout: diagnosis and management. NICE guideline. Reference number: NG219. Last updated: June 9 2022. https://www.nice.org.uk/guidance/ng219/chapter/Recommendations. Accessed 3 Dec 2025

[CR178] National Institute for Health and Care Excellence (NICE) (2023) Hypertension in pregnancy: diagnosis and management. NICE guideline. Reference number: NG133. https://www.nice.org.uk/guidance/ng133/chapter/recommendations. Last updated 17 April 2023. Last accessed 1 Dec 2025

[CR179] National Institute for Health and Care Excellence (NICE) (2025b) Epilepsies in children, young people and adults. NICE guideline. Reference number: NG217. https://www.nice.org.uk/guidance/ng217. Last updated 30 Jan 2025. Last accessed 4 Dec 2025

[CR180] National Osteoporosis Guideline Group (NOGG) 2024 Clinical guideline for the prevention and treatment of osteoporosis. NOGG, UK. https://www.nogg.org.uk/sites/nogg/download/NOGG-Guideline-2024.pdf?v4. Last updated Dec 2024. Last accessed 7 Dec 2025

[CR181] Neely GA, Sabir S, Kohli A (2025) Neostigmine. StatPearls Publishing, Treasure Island (FL) 29261883

[CR182] Nevitt SJ, Marson AG, Tudur Smith C (2019) Carbamazepine versus phenytoin monotherapy for epilepsy: an individual participant data review. Cochrane Database Syst Rev 7:CD001911. 10.1002/14651858.CD001911.pub4 31318037 PMC6637502

[CR183] Newman-Lindsay S, Lakshminrusimha S, Sankaran D (2022) Diazoxide for neonatal hyperinsulinemic hypoglycemia and pulmonary hypertension. Children (Basel, Switzerland) 10(1):5. 10.3390/children10010005 36670556 PMC9856357

[CR184] Niemann S (2023) When (new) drugs don’t work: Mozambique faces alarming multidrug-resistant tuberculosis epidemic. Press Release. German Center for Infection Research (DZIF). https://www.dzif.de/en/when-new-drugs-dont-work-mozambique-faces-alarming-multidrug-resistant-tuberculosis-epidemic. Last updated 10 Nov 2023. Last accessed 30 Nov 2025

[CR185] Numa AH, Williams GD, Dakin CJ (2000) The effect of nebulized epinephrine on respiratory mechanics and gas exchange in bronchiolitis. AJRCCM 164(1). 10.1164/ajrccm.164.1.2008090 11435244

[CR186] Nwaru BI, Ekström M, Hasvold P (2020) Overuse of short-acting β2-agonists in asthma is associated with increased risk of exacerbation and mortality: a nationwide cohort study of the global SABINA programme. Eur Respir J 55(4):1901872, 1901872. 10.1183/13993003.01872-2019 31949111 PMC7160635

[CR187] Nyunt M, Plowe CV (2009) Malaria. Pharmacology and therapeutics, principles to practice. Chapter 82:1141–1170. 10.1016/B978-1-4160-3291-5.50086-X

[CR188] Obermann M, Holle D (2020) Prednisone in short-term prevention of episodic cluster headache (4645). Neurology 94(15_supplement):4645. 10.1212/WNL.94.15_supplement.4645

[CR189] Offor SJ, Iyanam, Akpanowo EM et al (2025) Balancing the therapeutic and adverse effects of oral prednisone/prednisolone: a systematic review. J Adv Med Pharm Sci. 10.9734/jamps/2025/v27i10822

[CR190] Ophthalmology Times (2009) Erythromycin shortage leads clinicians to try alternative therapies. https://www.ophthalmologytimes.com/view/erythromycin-shortage-leads-clinicians-try-alternative-therapies#:~:text=Increasing%20resistance,ointment%20form%2C%22%20he%20said. Last updated 15 Oct 2009. Last accessed 29 Nov 2025

[CR191] Ornetti P, Bouillet B, Denimal D (2025) New guidelines on glucocorticoid-induced adrenal insufficiency: the end of short synacthen test in rheumatology? RMD Open 2025(11):e005251. 10.1136/rmdopen-2024-005251 PMC11759876 39843354

[CR192] Ottaiano A, Santorsola M, Capuozzo M, Scala S (2025) Balancing immunotherapy and corticosteroids in cancer treatment: dilemma or paradox? Oncologist 30(3):oyaf045. 10.1093/oncolo/oyaf045 40163690 PMC11957262

[CR193] Padberg S (2015) 2.6- Anti-infective agents. Drugs during pregnancy and lactation. 3rd edn, pp 115–176. 10.1016/B978-0-12-408078-2.00007-X

[CR194] Paghdal KV, Schwartz RA (2009) Topical tar: back to the future. JAAD 61(2):294–302. 10.1016/j.jaad.2008.11.024 19185953

[CR195] Parry Smith WR, Papadopoulou A, Thomas E, Tobias A, Price MJ, Meher S, Alfirevic Z, Weeks AD, Hofmeyr GJ, Gülmezoglu AM, Widmer M, Oladapo OT, Vogel JP, Althabe F, Coomarasamy A, Gallos ID (2020) Uterotonic agents for first-line treatment of postpartum haemorrhage: a network meta-analysis. Cochrane Database Syst Rev 11(11):CD012754. 10.1002/14651858.CD012754.pub2 33232518 PMC8130992

[CR196] Pazderska A, Pearce SH (2017) Adrenal insufficiency - recognition and management. Clin Med (Lond) 17(3):258–262. 10.7861/clinmedicine.17-3-258 28572228 PMC6297573

[CR197] Peacocke EF, Myhre SL, Foss HS, Gopinathan U (2022) National adaptation and implementation of WHO Model List of Essential Medicines: a qualitative evidence synthesis. Plos Medicine. 10.1371/journal.pmed.1003944 PMC8956172 35275938

[CR198] Pearson R, and Butler A (2021) Glyceryl trinitrate: History, mystery, and alcohol intolerance. Molecules (Basel, Switzerland), 26(21), 6581. 10.3390/molecules26216581 PMC8587134 34770988

[CR199] Pearson JC, Gillett E, Gadri ND, Dionne B (2024) Tetracyclines, the old and the new: A narrative review. CMI Communications. Volume 2, Issue 1, March 2025, 105059. 10.1016/j.cmicom.2025.105059

[CR200] Perrotta FM, Ambrosino P, Lubrano E (2024) Long-term survival of methotrexate as first-line therapy in rheumatoid arthritis, psoriatic arthritis and undifferentiated arthritis. J Clin Med 13(24):7540. 10.3390/jcm13247540 39768461 PMC11727825

[CR201] Pfister H-W, Klein M et al (2023) Ambulant erworbene bakterielle Meningoenzephalitis im Erwachsenenalter, S2kLeitlinie, 2023. Deutsche Gesellschaft für Neurologie (Hrsg.), Leitlinien für Diagnostik und Therapie in der Neurologie. https://register.awmf.org/assets/guidelines/030-089l-S2k_Ambulant-erworbene-bakterielle-Meningoenzephalitis-im-Erwachsenenalter_2023-05.pdf. Last updated 28 April 2023. Last accessed 25 Nov 2025

[CR202] Pofe R, Caratti G, Ray DW, Tomlinson JW (2023) Treating the side effects of exogenous glucocorticoids; Can we separate the good from the bad?, Endocrine reviews, volume 44, issue 6, December 2023, Pages 975–1011. 10.1210/endrev/bnad016 PMC10638606 37253115

[CR203] Prashar J, Hazewinkel D, Gregson J et al (2025) Efficacy of spironolactone in reducing the risk of cardiovascular events and long-term mortality in those with resistant hypertension: findings from the Anglo-Scandinavian Cardiac Outcomes Trial. Eur Heart J 46(Supplement_1):ehaf784.3390. 10.1093/eurheartj/ehaf784.3390

[CR204] Pressler RM, Abend NS, Auvin S et al (2023) Treatment of seizures in the neonate: Guidelines and consensus-based recommendations—Special report from the ILAE Task Force on Neonatal Seizures. Epilepsia 64(10):2550–2570. 10.1111/epi.17745 37655702

[CR205] Public Health Agency of Canada (2024) Syphilis guide: treatment and follow-up. Sexually transmitted and blood-borne infections: Guides for health professionals. https://www.canada.ca/en/public-health/services/infectious-diseases/sexual-health-sexually-transmitted-infections/canadian-guidelines/syphilis/treatment-follow-up.html. Last updated 11 April 2024. Last accessed 29 Nov 2025

[CR206] Qaseem A, Cooney TG, Wilt TJ et al (2025) Prevention of episodic migraine headache using pharmacologic treatments in outpatient settings: a clinical guideline from the American College of Physicians. Ann Intern Med 178(3). 10.7326/ANNALS-24-01052 39899861

[CR207] Radiation Emergency Medical Management (REMM) (2025) Dimercaprol. U.S. Department of Health & Human Services. https://remm.hhs.gov/dimercaprol.htm. Last updated 13 Nov 2025. Last accessed 24 Nov 2025

[CR208] Rao SV, O'Donoghue ML, Ruel M et al (2025) 2025 ACC/AHA/ACEP/NAEMSP/SCAI guideline for the management of patients with acute coronary syndromes: a report of the American College of Cardiology/American Heart Association Joint Committee on Clinical Practice Guidelines. Circulation 151(13). 10.1161/CIR.0000000000001309 40014670

[CR209] Rasmussen AM, Jakobsen R, Strøm T, Carlsson M, Dahler-Eriksen B, Toft P (2015) More complications in patients with septic shock treated with dextran compared with crystalloids. Dan Med J 62(2):A5018 25634506

[CR210] Resuscitation Council UK (2021) Emergency treatment of anaphylaxis. Guidelines for healthcare providers. Working Group of Resuscitation Council UK. https://www.resus.org.uk/sites/default/files/2021-05/Emergency%20Treatment%20of%20Anaphylaxis%20May%202021_0.pdf. Last updated May 2021. Last accessed 7 Dec 2025

[CR211] Richardus JH, Tiwari A, Barth-Jaeggi T, Arif MA et al (2021) Leprosy post-exposure prophylaxis with single-dose rifampicin (LPEP): an international feasibility programme. Lancet Glob Health 9(1):e81–e90. 10.1016/S2214-109X(20)30396-X 33129378

[CR212] Robays J, Nyamowala G, Sese C, Betu Ku Mesu Kande V, Lutumba P, Van der Veken W, Boelaert M (2008) High failure rates of melarsoprol for sleeping sickness, Democratic Republic of Congo. Emerg Infect Dis 14(6):966–967. 10.3201/eid1406.071266 18507916 PMC2600279

[CR213] Robert Koch Institut (RKI) (2018) Toxoplasmose. RKI-Ratgeber. https://www.rki.de/DE/Aktuelles/Publikationen/RKI-Ratgeber/Ratgeber/Ratgeber_Toxoplasmose.html#:~:text=Am%20h%C3%A4ufigsten%20werden%20die%20folgenden,die%20pr%C3%A4%2D%20und%20postnatale%20Therapie. Last updated 18 Oct 2018. Last accessed 30 Nov 2025

[CR214] Rocha DMGC, Viveiros M, Saraiva M, Osório NS (2021) The neglected contribution of streptomycin to the tuberculosis drug resistance problem. Genes (Basel) 12(12):2003. 10.3390/genes12122003 34946952 PMC8701281

[CR215] Rote Liste (2025a) Penicillin G Infectopharm® 1/5/10 Mega. Fachinformation. Rote Liste Service GmbH. https://www.fachinfo.de/fi/pdf/021635/penicillin-g-infectopharm-r. Last updated March 2025. Last accessed 28 Nov 2025

[CR216] Rousan TA, Thadani U (2019) Stable angina medical therapy management guidelines: a critical review of guidelines from the European Society of Cardiology and National Institute for Health and Care Excellence. Eur Cardiol 14(1):18–22. 10.15420/ecr.2018.26.1 31131033 PMC6523058

[CR217] Rugel AR, Guzman MA, Taylor AB, Chevalier FD, Tarpley RS et al (2020) Why does oxamniquine kill *Schistosoma mansoni* and not *S. haematobium* and *S. japonicum*? Int J Parasitol Drugs Drug Resist 13:8–15. 10.1016/j.ijpddr.2020.04.001 32315953 PMC7167500

[CR218] Rzasa Lynn R, Galinkin JL (2018) Naloxone dosage for opioid reversal: current evidence and clinical implications. Therapeutic Advances in Drug Safety 9(1):63–88. 10.1177/2042098617744161 29318006 PMC5753997

[CR219] Salah AB, Messaoud NB, Guedri EG et al (2013) Topical Paromomycin with or without Gentamicin for Cutaneous Leishmaniasis. N Engl J Med 2013(368):524–532. 10.1056/NEJMoa1202657 23388004

[CR220] Savarese G, Becher PM, Lund LH, Seferovic P, Rosano GMC, Coats AJS (2023) Global burden of heart failure: a comprehensive and updated review of epidemiology. Cardiovasc Res 118(17):3272–3287. 10.1093/cvr/cvac013 35150240

[CR221] Schachtel BP, Voelker M, Sanner KM, Gagney D, Bey M, Schachtel EJ, Becka M (2010) Demonstration of the analgesic efficacy and dose-response of acetylsalicylic acid with pseudoephedrine. J Clin Pharmacol 50(12):1429–1437. 10.1177/0091270009360978 20350952

[CR222] Schlitzer M (2010) Lebensrettende Prophylaxe und Therapie. Malaria. Pharmazeutische Zeitung, 12/2010. https://www.pharmazeutische-zeitung.de/ausgabe-122010/lebensrettende-prophylaxe-und-therapie/. Last updated 22 March 2010. Last accessed 25 Nov 2025

[CR223] Seixas J, Atouguia J, Josenando T, Vatunga G, Miaka Mia Bilenge C, Lutumba P, Burri C (2020) Clinical study on the melarsoprol-related encephalopathic syndrome: risk factors and HLA association. Trop Med Infect Dis 5(1):5. 10.3390/tropicalmed5010005 31906333 PMC7157710

[CR224] Semenova Y, Makalkina L, Glushkova N, Gaipov A (2025) Tetracyclines in the modern era: global consumption, antimicrobial resistance, environmental occurrence, and degradation techniques. Antibiotics (Basel) 14(12):1183. 10.3390/antibiotics14121183 41463684 PMC12729376

[CR225] Seo MK, Yoon JS, So CH, Lee HS, Hwang JS (2017) Intellectual development in preschool children with early treated congenital hypothyroidism. Annals of Pediatric Endocrinology & Metabolism 22(2):102–107. 10.6065/apem.2017.22.2.102 28690988 PMC5495975

[CR226] Sethi S, Sethi A (2025) Adverse outcomes associated with unscrupulous ocular use of topical chloramphenicol: a case report and brief review of literature. TNOA J Ophthalmic Sci Res 63(3):264–267. 10.4103/tjosr.tjosr_31_25

[CR227] Shanks GD (2016) Historical Review: Problematic Malaria Prophylaxis with Quinine. Am J Trop Med Hyg 95(2):269–272. 10.4269/ajtmh.16-0138 27185766 PMC4973170

[CR228] Shea BJ, Adachi JD, Cranney A, Griffith L, Guyatt G, Hamel C, Ortiz Z, Peterson J, Robinson VA, Tugwell P, Wells G, Zytaruk N (2004) Calcium supplementation on bone loss in postmenopausal women. Cochrane database of systematic reviews 2004, Issue 1. Art. No.: CD004526. 10.1002/14651858.CD004526.pub2 14974070

[CR229] Sigera LSM, Denning DW (2023) Flucytosine and its clinical usage. Ther Adv Infect Dis 10:20499361231161387. 10.1177/20499361231161387 37051439 PMC10084540

[CR230] Singh P, Gupta S, Abidi A, Krishna A (2013) Comparative evaluation of topical calcipotriol versus coal tar and salicylic acid ointment in chronic plaque psoriasis. Journal of Drugs in Dermatology : JDD 12(8):868–873 23986159

[CR231] Sinha SS, Morrow DA, Kapur NK et al (2025) 2025 Concise clinical guidance: an ACC expert consensus statement on the evaluation and management of cardiogenic shock: a report of the American College of Cardiology Solution Set Oversight Committee. JACC 85(16). 10.1016/j.jacc.2025.02.018 40100174

[CR232] Sisay M, Hailu T, Damtie D et al (2025) Efficacy of single dose praziquantel against *Schistosoma mansoni* in East Africa: a systematic review and meta-analysis. Sci Rep 15:4642. 10.1038/s41598-024-84621-8 39920188 PMC11806046

[CR233] Sivakumaran P, Barros B, Antonio Dias VL, Lockwood DN, Walker SL (2024) A retrospective cohort study of monthly rifampicin, ofloxacin and minocycline in the management of leprosy at the Hospital for Tropical Diseases, London, United Kingdom. PLoS Negl Trop Dis 18(12):e0012699. 10.1371/journal.pntd.0012699 39652597 PMC11658689

[CR234] Slutsky JB, Clark RA, Remedios AA, Klein PA (2010) An evidence-based review of the efficacy of coal tar preparations in the treatment of psoriasis and atopic dermatitis. Journal of Drugs in Dermatology : JDD 9(10):1258–1264 20941951

[CR235] Smith-Norowitz TA, Ukaegbu C, Kohlhoff S, Hammerschlag MR (2021) Neonatal prophylaxis with antibiotic containing ointments does not reduce incidence of chlamydial conjunctivitis in newborns. BMC Infect Dis 21(1):270. 10.1186/s12879-021-05974-3 33731049 PMC7971948

[CR236] Song B, Liu L, Dong S, Wang S (2025) Drug-induced hypokalemia: an analytical study based on real-world drug monitoring data. Expert Opin Drug Saf 1–9. 10.1080/14740338.2025.2468861 39977281

[CR237] South East Asian Quinine Artesunate Malaria Trial group (SEAQUAMAT) (2005) Artesunate versus quinine for treatment of severe falciparum malaria: a randomised trial. The Lancet 366(9487):717–725. 10.1016/S0140-6736(05)67176-0 16125588

[CR238] Sprute R, Duda S, Liekweg A, Simon M, Cornely OA (2023) The silent flucytosine shortage in Europe – not a distant problem. Lancet Reg Health Eur 30:100658. 10.1016/j.lanepe.2023.100658 37293188 PMC10245318

[CR239] St-Onge M, Anseeuw K, Cantrell FL et al (2017) Experts consensus recommendations for the management of calcium channel blocker poisoning in adults. Crit Care Med 45(3):e306-e315. 10.1097/CCM.0000000000002087 PMC5312725 27749343

[CR240] Sundar S, Chakravarty J (2008) Paromomycin in the treatment of leishmaniasis. Expert Opin Investig Drugs 17(5):787–794. 10.1517/13543784.17.5.787 18447603

[CR241] Taudorf EH, Jemec GBE, Hay RJ, Saunte DML (2019) Cutaneous candidiasis - an evidence-based review of topical and systemic treatments to inform clinical practice. Journal of the European Academy of Dermatology and Venereology : JEADV 33(10):1863–1873. 10.1111/jdv.15782 31287594

[CR242] Taylor ZL, Vang J, Lopez-Lopez E, Oosterom N, Mikkelsen T, Ramsey LB (2021) Systematic review of pharmacogenetic factors that influence high-dose methotrexate pharmacokinetics in pediatric malignancies. Cancers 13(11):2837. 10.3390/cancers13112837 34200242 PMC8201112

[CR243] Thilen SR, Weigel WA, Todd MM (2023) 2023 American Society of Anesthesiologists practice guidelines for monitoring and antagonism of neuromuscular blockade: a report by the American Society of Anesthesiologists task force on neuromuscular blockade. Anesthesiology 138(1):13–41. 10.1097/ALN.0000000000004379 36520073

[CR244] Tinawi M (2020) Hypokalemia: a practical approach to diagnosis and treatment. Arch Clin Biomed Res 4:048–066. https://fortuneonline.org/articles/hypokalemia-a-practical-approach-to-diagnosis-and-treatment.html?url=hypokalemia-a-practical-approach-to-diagnosis-and-treatment

[CR245] Tran HA, Merriman E, Baker R, Curnow J, Young L, Tan CW, McRae S, Chunilal SD (2025) 2025 Guidelines for direct oral anticoagulants: a practical guidance on the prescription, laboratory testing, peri-operative and bleeding management. Intern Med J 55(7):1174–1183. 10.1111/imj.70103 40448969 PMC12240022

[CR246] U.S. Centers for Disease Control and Prevention (CDC) (2024b) Syphilis. Sexually transmitted infections treatment guidelines, 2021. https://www.cdc.gov/std/treatment-guidelines/syphilis.htm. Last updated 03 Oct 2024. Last accessed 29 Nov 2025

[CR247] UK Clinical Pharmacy Association (UKCPA) (2025) Calcium gluconate. Handbook of Perioperative Medicines. https://periop-handbook.ukclinicalpharmacy.org/drug/calcium-gluconate/. Last updated 2025. Last accessed 7 Dec 2025

[CR248] United States Drug Enforcement Adimistration (DEA) (2025) Save Lives with Naloxone. U.S. Department of Justice. https://www.dea.gov/onepill/save-lives. Last updated 2025. Last accessed 4 Dec 2025

[CR249] Urban PP, Jacobi C, Jander S (2018) Treatment standards and individualized therapy of myasthenia gravis. Neurol Int Open 2:E84–E92. 10.1055/s-0043-124983

[CR250] U.S. Centers for Disease Control and Prevention (CDC) (2024c) Clinical Care of Toxoplasmosis. Last updated: January 22 2024. https://www.cdc.gov/std/treatment-guidelines/syphilis.htm. Accessed 29 Nov 2025

[CR251] van Prehn J, Reigadas E, Vogelzang EH, Fitzpatrick F et al (2021) European Society of Clinical Microbiology and Infectious Diseases: 2021 update on the treatment guidance document for *Clostridioides difficile* infection in adults. Clin Microbiol Infect. 10.1016/j.cmi.2021.09.038 34678515

[CR252] Varriale P (1999) Role of dopamine in congestive heart failure: a contemporary appraisal. Congestive heart failure (Greenwich, Conn.) 5(3):120–124 12189316

[CR253] Verma A, Jaiswal S, Reid C, Borah P, Lal M, Gupta S, Khanna P (2024) Efficacy of 10%,25% and 50% dextrose in the treatment of hypoglycemia in the emergency department - A randomized controlled study. Am J Emerg Med 82:101–104. 10.1016/j.ajem.2024.05.029 38851077

[CR254] Versijpt J, Deligianni C, Hussain M, Amin F, Reuter U, Sanchez-Del-Rio M, Uluduz D, Boucherie D, Zeraatkar D, MaassenVanDenBrink A, Sacco S, Lampl C, Gil-Gouveia R (2024) European Headache Federation (EHF) critical re-appraisal and meta-analysis of oral drugs in migraine prevention - part 4: propranolol. J Headache Pain 25(1):119. 10.1186/s10194-024-01826-y 39044170 PMC11267726

[CR255] Virgadamo S, Charnigo R, Darrat Y, Morales G, Elayi CS (2015) Digoxin: A systematic review in atrial fibrillation, congestive heart failure and post myocardial infarction. World J Cardiol 7(11):808–816. 10.4330/wjc.v7.i11.808 26635929 PMC4660476

[CR256] Visser MT, Zonneveld R, Peto TJ, van Vugt M, Dondorp AM (2022) Are national treatment guidelines for falciparum malaria in line with WHO recommendations and is antimalarial resistance taken into consideration? – A review of guidelines in non-endemic countries. TMIH 27(2):129–136. 10.1111/tmi.13715 PMC9304135 34978744

[CR257] Wagner IV, Stewart MW, Dorairaj SK (2022) Updates on the diagnosis and management of glaucoma. Mayo Clin Proc 6(6):p618-635. 10.1016/j.mayocpiqo.2022.09.007 PMC9673042 36405987

[CR258] Walsh R, Doherty CP, Doran E (2025) The use of steroids in adult epilepsy: A systematic review. Epilepsia Open 10(2):398–410. 10.1002/epi4.13019 39936489 PMC12014922

[CR259] Wang Y, Lipner SR (2020) Retrospective analysis of adverse events with spironolactone in females reported to the United States Food and Drug Administration. Int J Womens Dermatol 6(4):272–276. 10.1016/j.ijwd.2020.05.002 PMC7522893 33015285

[CR260] Waters M, Tadi P (2023) Streptomycin. StatPearls, Treasure Island (FL). https://www.ncbi.nlm.nih.gov/books/NBK555886/. Last updated 4 July 2023. Last accessed 30 Nov 2025

[CR261] Watjer RM, Bonten TN, Sayed K et al (2024) How effective is topical miconazole or amorolfine for mild to moderately severe onychomycosis in primary care: the Onycho Trial – a randomised double-blind placebo-controlled trial. BMJ Open 14:e081914. 10.1136/bmjopen-2023-081914 PMC11086198 38702077

[CR262] Wee Y, Burns K, Bett N (2015) Medical management of chronic stable angina. Aust Prescr 38:131-36. 10.18773/austprescr.2015.042 PMC4653970 26648642

[CR263] Weinstein R, Naber CE, Brumme K (2024) Revisiting dexamethasone use in the pediatric emergency department. Curr Opin Pediatr 36(3):251-255. 10.1097/MOP.0000000000001351 38655807

[CR264] White NJ (2014) Malaria. Manson's tropical infectious diseases. Chapter 43, 23th edn, pp 532-600.e1. 10.1016/B978-0-7020-5101-2.00044-3

[CR265] Whyte AF, Soar J, Dodd A, Hughes A, Sargant N, Turner PJ (2022) Emergency treatment of anaphylaxis: concise clinical guidance. Clin Med (London, England) 22(4):332–339. 10.7861/clinmed.2022-0073 PMC9345203 35882481

[CR266] Whyte LE (2023) America has shortage of key lead-poisoning drug. The Wall Street Journal. https://www.wsj.com/health/lead-poisoning-drug-shortage-e485515b. Last updated 11 July 2023. Last accessed 24 Nov 2025

[CR267] Wijdicks EFM (2024) Corticosteroids in acute neurology and neurosurgery: promises, promises, promises. Neurocrit Care. 10.1007/s12028-024-02154-4 39532776

[CR268] Wollenberg A, Kinberger M, Arents B et al (2025) European Guideline (EuroGuiDerm) on atopic eczema: Living update. JEADV 39(9):1537-1566. https://www.guidelines.edf.one/guidelines/atopic-ezcema 10.1111/jdv.20639 PMC12376260 40317496

[CR269] World Health Organization (WHO) (1977) The selection of essential drugs. Report of a WHO Expert Committee. WHO Technical Report Series 615. WHO, Geneva, 1977. ISBN 92-4-120615-2

[CR270] World Health Organization (WHO) (2006b) Oral Rehydration Salts. Department of Child and Adolescent Health and Development (CAH), World Health Organization, Geneva. WHO Reference Number: WHO/FCH/CAH/06.1. https://iris.who.int/server/api/core/bitstreams/e52f1746-cf33-4a63-9f97-4c0bfaaeb230/content. Last updated 2006. Last accessed 6 Dec 2025

[CR271] World Health Organization (WHO) (2013) Guidelines for measles and rubella outbreak investigation and response in the WHO European Region. Regional office for Europe, World Health Organization. https://cdn.who.int/media/docs/librariesprovider2/euro-health-topics/vaccines-andimmunization/outbreakguidelines-updated.pdf?sfvrsn=6f0c7b0d_1&download=true. Last updated 2013. Last accessed 6 Dec 2025

[CR272] World Health Organization (2016) WHO Guidelines for the Treatment of Treponema pallidum (Syphilis). Geneva: World Health Organization. https://www.ncbi.nlm.nih.gov/books/NBK384905/. Last updated 2016. Last accessed 29 Nov 2025

[CR273] World Health Organization (WHO) (2018a) WHO guidelines for the pharmacological and radiotherapeutic management of cancer pain in adults and adolescents. WHO, Geneva, 2018. ISBN 978-92-4-155039-0 30776210

[CR274] World Health Organization (WHO) (2018c) Typhoid vaccines: WHO position paper. Wkly Epidemiol Rec 93:13. https://iris.who.int/server/api/core/bitstreams/78472e52-a4f4-483f-90da-15d5c1110dfc/content. Last updated Mar 2018. Last accessed 6 Dec 2025

[CR275] World Health Organization (WHO) (2022) Updated WHO recommendations for malaria chemoprevention among children and pregnant women. Last updated: June 3 2022. https://www.who.int/news/item/03-06-2022-Updated-WHO-recommendations-for-malaria-chemoprevention-among-children-and-pregnant-women. Accessed 29 Nov 2025

[CR276] World Health Organization (WHO) (2025a) The selection and use of essential medicines. Report of the 25th WHO Expert Committee on Selection and Use of Essential Medicines, executive summary. WHO, Geneva. 10.2471/B09544

[CR277] World Health Organization (WHO) (2025b) The selection and use of essential medicines, 2025. WHO Model List of Essential Medicines, 24th list. WHO, Geneva. 10.2471/B09474

[CR278] World Health Organization (WHO) (2025c) Ensuring demand and supply of benzathine penicillin to treat syphilis. Global sexually transmitted infections programme. Last updated: 2025. https://www.who.int/teams/global-hiv-hepatitis-and-stis-programmes/stis/treatment/shortages-of-penicillin. Accessed 27 Nov 2025

[CR279] World Health Organization (WHO) (2026) Expert commitee on selection and use of essential medicines. WHO. https://www.who.int/groups/expertcommittee-on-selection-and-use-of-essentialmedicines#:~:text=audit%20and%20investigations-,Expert%20Committee%20on%20Selection%20and%20Use%20of%20Essential%20Medicines,Medicines%20for%20Children%20(EMLc). Last accessed 29 Apr 2026

[CR280] Wu L, Rodriguez M, El Hachem K, Krittanawong C (2024) Diuretic treatment in heart failure: a practical guide for clinicians. J Clin Med 13(15):4470. 10.3390/jcm13154470 PMC11313642 39124738

[CR281] Xiong CY, Yang YM, Zhou Y, He TS, Luo XW, Wang J, Mao CX (2024) Systematic analysis of the adverse effects of commonly used clinical tetracycline drugs based on the FAERS database. Expert Opin Drug Saf 24(8):949–957. 10.1080/14740338.2024.2392865 39140181

[CR282] Yang C, Chen C, Deng S, Deng ST et al (2015) Allopurinol use and risk of fatal hypersensitivity reactions: a nationwide population-based study in Taiwan. JAMA Intern Med 175(9):1550–1557. 10.1001/jamainternmed.2015.3536 26193384

[CR283] Yasir M, Goyal A, Sonthalia S (2023) Corticosteroid adverse effects. StatPearls Publishing, Treasure Island (FL). https://www.ncbi.nlm.nih.gov/books/NBK531462. Last updated 3 July 2023. Last accessed 6 Dec 2025 30285357

[CR284] Yeka A, Achan J, D'Alessandro U, Talisuna AO (2009) Quinine monotherapy for treating uncomplicated malaria in the era of artemisinin-based combination therapy: an appropriate public health policy?. Lancet Infect Dis 9(7):448-452. 10.1016/S1473-3099(09)70109-4 19555904

[CR285] Yilmaz M, Gürses D, Tükenmez G (2022) The effectiveness and safety of ibuprofen and acetylsalicylic acid in acute rheumatic fever. Pediatr Int 64(1):e15133. 10.1111/ped.15133 35704468

[CR286] Zellner T, Prasa D, Färber E, Hoffmann-Walbeck P, Genser D, Eyer F (2019) The use of activated charcoal to treat intoxications. Deutsches Arzteblatt Int 116(18):311–317. 10.3238/arztebl.2019.0311 PMC6620762 31219028

[CR287] Zhang H, Yan R, Liu Y, Yu M, He Z, Xiao J, Li K, Liu G, Ning Q, Li Y (2025) Progress in antileishmanial drugs: mechanisms, challenges, and prospects. PLoS Neglected Trop Dis 19(1):e0012735. 10.1371/journal.pntd.0012735 PMC11698350 39752369

[CR288] Zhou Y, Yang H, Zhang N, Chen M, Jiang Y, Rui Q (2017) Dopamine versus norepinephrine in the treatment of cardiogenic shock: A PRISMA-compliant meta-analysis. Medicine 96(43):e8402. 10.1097/MD.0000000000008402 PMC5671870 29069037

[CR289] Ziff OJ, Kotecha D (2016) Digoxin: The good and the bad. Trend Cardiovasc Med 26(7):585–595. 10.1016/j.tcm.2016.03.011 27156593

