# Supplementary material for: Pharmacological assessment of drugs retained in the 1st and 24th WHO Essential Medicines List (1977–2025)
Source: Naunyn Schmiedebergs Arch Pharmacol. 2026 Jun 17;399(12):19047–75. doi: 10.1007/s00210-026-05555-8 (PMC13593693; doi:10.1007/s00210-026-05555-8)
Supplement: Supplementary file 1 [file 210_2026_5555_MOESM1_ESM.docx]

**Pharmacological assessment of drugs retained in the 1st and 24th WHO Essential Medicines List (1977–2025)**

**Lilly Josephine Bindel and Roland Seifert**

**Institute of Pharmacology**
**Hannover Medical School**
**D-30625 Hannover, Germany**
**Corresponding author: Roland Seifert**
**seifert.roland@mh-hannover.de**

***Table S1:*** *Assessment of retained drugs, representing medicines listed in both the 1st and 24th editions of the EML. A comprehensive overview of all listed drugs, considered changes, and assessments is given. The assessment of listed indications uses colour coding: light green for no change or specification, dark green for the addition of indications, blue for a change of indication, and yellow for a narrowed indication. Additionally, guideline recommendations, efficacy and safety, scope of clinical usage, and public health relevance were evaluated to determine each drug’s importance for its listed indications and to provide a recommendation on whether the listing is justified or warrants discussion for potential deletion in the next EML edition. A brief explanation accompanies each assessment. Drugs considered for deletion are highlighted in red, and drugs not recommended for specific listed indications are highlighted in yellow.*

| **Retained drugs** | **Indication** | | | **Evidence of efficacy and safety for listed indications** | | | **Assessment** | | |
| --- | --- | --- | --- | --- | --- | --- | --- | --- | --- |
|  | **Indication EML 1977 (WHO 1977)** | **Indication EML 2025 (WHO 2025b)** | **Change** | **Recommendation in guidelines** | **Effectiveness and safety** | **Public health need** | **Suggestion** | **Development of clinical use** | **Explanation** |
| dimercaprol | intoxication (antidote) | intoxication (antidote) | no | heavy metal intoxiation (lead, arsenic, mercury) (Dawn and Whited 2023; Braitberg 2019) | effective but significant toxicity (Long 2020; Dawn and Whited 2023; REMM 2025) | declining usage because availability of more favourable alternatives like DMPS, problems with availability (Gerhardsson and Aaseth 2016; Whyte 2023) | deletion | declining | supply shortages, high toxicity, availability of less toxic alternatives (Long 2020; Mückter et al. 1997) |
| amodiaquine | malaria | malaria (curative, chemoprevention) in FDC | narrowed and specified indication, use only in FDC | curative treatment of malaria, but not recommended for chemoprevention (MSF 2025c; Padberg 2015) | effective, but toxicity and fatal adverse events are an issue, increasing resistance (Schlitzer 2010; AlKadi 2007; White 2014; Nyunt and Plowe 2009) | not anymore recommended for long-term use, reserve malaria agent (Schlitzer 2010; Padberg 2015) | deletion | declining | toxicity issues, no longer used in Europe, USA and WHO control programms, availabilty of safer alternatives (Schlitzer 2010; Aronson 2016; Nyunt and Plowe 2009) |
| ampicillin | bacterial infections (not specified) | second choice for acute bacterial meningitis | narrowed and specified indication | only as an alternative for meningitis (Infectious Diseases Society of America 2025; Pfister et al. 2023) | decreasing efficacy due to high bacterial resistance rates, acceptable safety (Kaushik et al. 2014; Heinz 2018; FDA 2023) | not anymore recommended as first-choice, decreasing use (Infectious Diseases Society of America 2025; Pfister et al. 2023) | deletion | declining | high bacterial resistance rates, available alternatives which are used more frequently, limited indication and no recommendation as first-line choice/ only as an alternative (Infectious Diseases Society of America 2025; Kaushik et al. 2015) |
| chloramphenicol | bacterial infections (not specified) | second choice for acute bacterial meningitis | narrowed and specified indication | only as reserve, not first choice for meningitis (EMC 2022; Moffa and Brook 2015; Sethi and Sethi 2025) | effective, but toxicity issues (e.g. aplastic anaemia) (Moffa and Brook 2015; Sethi and Sethi 2025; BfR 2014) | limited use (EMC 2022; Moffa and Brook 2015; Sethi and Sethi 2025) | deletion | declining | toxicity issues, favouring of safer alternatives (BfR 2014; Deutsches Ärzteblatt 2017) |
| erythromycin | bacterial infections (not specified) | ophthalmologic infections (*C. trachomatis, N. gonorrhoea*) | narrowed and specified indication | yes, but not effective in chlamydial infections, availability of alternatives (AAO 2025; Smith-Norowith et al. 2021; Duff 2015) | yes in susceptible pathogens in ocular use but increasing bacterial resistance, acceptable safety, availability of alternatives (AAO 2025; NHS 2022a; Kim an Toma 2011; Cabrera-Aguas et al. 2024; Hsueh 2005) | rarely used: already replaced in systemic therapy, availability of alternatives in ophthalmology (Hsueh 2005; Amsden 1996; Ophthalmology Times 2009; Bremond-Gignac et al. 2011) | deletion | declining | obsolete (decreasing use, high resistance rates, supply shortages), established use of more favourable alternatives (Amsden 1996; Duff 2015; Ophthalmology Times 2009) |
| griseofulvin | fungal infections | fungal infections | no | only in dermatophytes, replaced by other drugs in other indications (Aris et al. 2022; Bonifaz et al. 2024) | effective but comparable (dermatophytes) and inferior (other fungal infections) to newer drugs (Bar et al. 2019; Möhrenschlager et al. 2002) | declining use, not marketed anymore in many countries (DocCheck 2025; Bonifaz et al. 2024) | deletion | declining | obsolete, not available, comparable/ better alternatives (terbinafine, azoles) (Bonfaz et al. 2024) |
| melarsoprol | trypanosomial infection | trypanosomial infection (2nd stage African) | specification | reserve in trypanosomiasis (Lindner et al. 2025) | decreasing effectiveness, high toxicity (Robays et al. 2008; Seixas et al. 2020) | declining, replacement by alternatives (Lindner et al. 2025) | deletion | declining | declining use due to availability of safer alternatives, toxicity issues (Lindner et al. 2025; Seixas et al. 2020) |
| oxamniquine | schistosomial infections | schistosomial infections | no | reserve, mostly replaced by praziquantel (Danso-Appiah et al. 2022) | effective in some settings, less broad spectrum than alternatives, increasing resistance (Alwan et al. 2023; Rugel et al. 2020; Chevalier et al. 2019) | rarely used, praziquantel preferred (Sisay et al. 2025; Danso-Appiah et al. 2022) | deletion | declining | obsolete, preferation of praziquantel, reserve (Danso-Appiah et al. 2022) |
| procaine benzylpenicillin | bacterial infections (not specified) | Access group: second choice syphilis | narrowed and specified indication | alternative but not first choice in syphilis (WHO 2016; CDC 2024b; MSHC 2021) | increasing bacterial resistance, inferior to other second choice alternatives and need of frequent injections (CDC 2024b; Public Health Agency of Canada 2024; Greenwood and Irving 2012) | rarely used, only if no other treatment is available (MSHC 2021) | deletion | declining | not recommended in use, inferior to other reserve drugs, not generally used (Public Health Agency of Canada; MSHC 2021) |
| quinine | malaria | malaria (curative) | no | not recommended in WHO guidelines for malaria, listed as alternative in some national guidelines (Visser et al. 2022) | substantial disadvantages in comparison to alternatives like artesunate (high toxicity, narrower therapeutic index, resistance issues and decreased efficacy, dosing) (Shanks 2016; Yeka et al. 2009; Cramer eta l. 2011 | use not recommended, inferior to alternatives (Yeka et al. 2009; SEAQUAMAT 2005) | deletion | declining | ineffective, availability of superior alternatives, not recommended in public health, usage needs to be discouraged (Yeka et al. 2009; Visser et al. 2022) |
| streptomycin | tuberculosis | tuberculosis | no | second-line for tuberculosis, but preference of other reserve drugs (Ministry of Health and Family Welfare 2025; Cohen et al. 2020; Michalik et al. 2025) | high resistance rates, toxicity issues (ototoxicity, nephrotoxicity), preference of newer alternatives as first line and amikacin as second line (Cohen et al. 2020; Rocha et al. 2021; Guo et al. 2025; Niemann 2023; Waters and Tadi 2023) | reserve, declining use and withdrawal from guidelines (Cohen et al. 2020; Ministry of Health and Family Welfare 2025) | deletion | declining | not recommended as first-choice, preference of other reserve options, high resistance, toxicity issues (Rocha et al. 2021; Guo et al. 2025 |
| tetracycline | bacterial infections (not specified) | opthalmologic infections (bacterial blepharitis, conjunctivitis, keratitis, trachoma) | narrowed and specified indication | only recommened as use in eye drops in ophthalmology but not recommended in systemic therapy (MSF 2025e) | effective in ophthalmologic conditions but availability of newer and favoured drugs, high bacterial resistance rates, unfavourable adverse effects in comparison with alternatives (Pearson et al. 2025; Xiong et al. 2024; Griffin et al. 2010; Bremond-Gignac et al. 2011) | niche, declining use and importance, replaced by more favourable antibacterial drugs (Semenova et al. 2025; LaPlante et al. 2022) | deletion | declining | obsolete, high bacterial resistance rates, narrow indications, serious adverse events, availability of more effective, safe and appropriate alternatives (Bremond-Gignac et al. 2011; Semenova et al. 2025) |
| ***Table S1 (continued)*** | | | | | | | | | |
| ***Retained drugs*** | ***Indication EML 1977*** | ***Indication EML 2025*** | ***Change*** | ***Recommendation in guidelines*** | ***Effectiveness and safety*** | ***Public health need*** | ***Suggestion*** | ***Clinical use*** | ***Explanation*** |
| dextran | plasma substitute | plasma substitute | no | alternatives often preferred depending on indication (crystalloids, albumin) (Miao and Guthmiller 2025) | effective volume expander, but comparably risk of adverse events (anaphylaxis and coagulopathy), other crystalloids favoured for many indications (Isbister and Fisher 1980; Martin 2019; Rasmussen et al. 2015) | declining, historical use, now rarely used and crystalloids are first-line (Martin 2019; Baglin 2012) | deletion | declining | obsolete, availability of safer and cheaper crystalloids in most indications (Martin 2019; Miao and Guthmiller 2025) |
| digoxin | cardiac glycoside (not specified) | arrhythmia, heart failure | specification | not first-line, partly used as an adjunctive therapy for atrial fibrillation and symptomatic heart failure, availability of superior alternatives (Crane et al. 2024; Chen et al. 2015) | evidence for symptomatic improvement but limited mortaily benefit, narrow therapeutic index and arrhythmia risk require monitoring (Virgadamo et al. 2015; Jabri et al. 2022) | marginal importance because availability of superior alternatives (Ziff and Kotecha 2016; Crane et al. 2024; Chen et al. 2015) | deletion | declining | obsolete because availability of better alternatives and issues with narrow therapeutic index |
| dopamine | cardiovascular shock | heart failure | change of indication | not first-line in heart failure /acute treatment (Becerra et al. 2025; Bloom et al. 2023) | moderately effective but preference of norepinephrine and doputamine, use is associated with higher mortality (Becerra et al. 2025; Varriale 1999; Hosoya et al. 2025; Zhou et al. 2017) | declining use, not recommended (Becerra et al. 2025) | deletion | declining | favouring of alternatives like norepinephrine and dobutamine in acute situations, no role in long-term treatment |
| glyceryl trinitrate | angina | angina | no | short-acting anti-anginal for acute chest parin, but preference of other alternatives (Arden 2011; Rousan and Thadani 2019; NICE 2016a) | symptomatic improvement, but contraindicated for long-term use (tolerance development), risks of severe adverse events (Pearson and Butler 2021; Arden 2011; Wee et al. 2015; Münzel et al. 2005) | declining use, alternatives like nitrogylcerin more favourable (NICE 2016a; Alyavi and Uzokov 2017) | deletion | declining | obsolete because availability of safer and more appropriate alternatives, no proven benefit in outcomes, no role in long term treatment (Arden 2011) |
| miconazole | fungal infections, topical | fungal infections, topical | no | alternative for topical treatment of superficial fungal infections but not first-line (Mayser and Niehaus 2023; Bortolussi and Martin 2024) | good efficacy but higher rate of adverse effects and drug interactions than available and favoured alternatives, available as OTC (GOV UK 2017; Gamil et al. 2024; Mijalijica et al. 2022; Watjer et al. 2023; Taudorf et al. 2019; Li 2023) | high global prevalence of superficial mycoses (Ma et al. 2025; Havlickova et al. 2008) | deletion | declining | effective but safety concerns, not recommended in guidelines, availability of comparably efficious but safer alternatives |
| pilocarpine | miotic, topical | miotic/glaucoma | no | historic alternative for acute angle-closure and chronic glaucoma, not recommended as first choice (Wagner et al. 2022) | effective but high number of adverse effects, availability of more favourable alternatives, not recommended as first choice (NICE 2022a; Li et al. 2025; Ludwig and Seifert 2025) | declining, replacement by newer drugs (Ludwig and Seifert 2025) | deletion | declining | obsolete, not recommended as first choice, availability of safer and more effective drugs (e.g. carboanhydrase inhbitors) (Wagner et al. 2022; Ludwig and Seifert 2025) |
| neostigmine | peripheral muscle relaxant (not specified) | myastenia gravis, muscle relaxant | specification | alternative for reversal of neuromuscular blockade and myasthenia gravis, not first choice (Neely et al. 2025; Thilen et al. 2023) | inferior to suxamethonium in anesthesia and pyridostigmine in mystenia gravis, less effective and more adverse effects (Urban e tal. 2018; Fiorda Diaz et al. 2022; Mehndiratta et al. 2014; Neely et al. 2025) | declining, but still used in some parts of the world (Urban et al. 2018) | deletion | declining | inferior to more effective and safe alternatives (e.g. pyridostigmine, summagadex), not recommended as first choice in guidelines (Hristovska et al. 2017; Jacob et al. 2025) |
| atropine | intoxication (antidote), antispasmodic (gastrointestinal) | antidote, preoperative medication for short-term procedures, mydriatic (ophthalmology) | addition for operative procedures and opthalmology, deletion as an antispasmodic | yes (Patel et al. 2025; Plaud et al. 2020; Gorman et al. 2003) | effective and acceptable safety (Plaud et al. 2020; Chen et al. 2024; Wigemstam et al. 2022) | yes (Gorman et al. 2003; Plaud et al. 2020; Kaiti et al. 2022) | retained | constant | essential in emergency care and anaesthesia, widespread use |
| charcoal, activated | intoxication (antidote) | intoxication (antidote) | no | yes (Zellner et al. 2019) | safe, but efficacy variable across toxins (Hartman et al. 2025; Jrayed et al. 2024) | standard drug for intoxication (Hartmann et al. 2025; Zellner et al. 2019) | retained | constant | need in emergency care, established use in acute poisoning |
| benzathine benzylpenicillin | bacterial infections (not specified) | Access group: syphilis | narrowed and specified indication | first choice in syphilis (CDC 2021; Janier et al. 2021) | effective and safe (Jaiswal et al. 2025; CDC 2021; Janier et al. 2021) | widely used, but issues of supply shortages (WHO 2025c; Seghers et al. 2025) | retained | constant | widespread use and effective for syphilis, restrictions due to global supply shortages (WHO 2025c) |
| chloroquine | malaria | malaria (curative, chemoprevention), anti-rheumatic (DMARDs) | addition of immunomodulatory conditions | yes, in malaria only when sensitivity confirmed (Frosch et al. 2014; FDA 2020) | widespread resistance in malaria , effective for rheumatology (Price et al. 2014; Torres et al. 2013; Ates et al. 2025) | used in treatment of malaria (Ates et al. 2025; Nirk et al. 2020) | retained | constant | established use in malaria, increasing importance for inflammatory conditions |
| clofazimine | leprosy | Reserve group: leprosy, tuberculosis | addition of tuberculosis | yes (FDA 2023; Stadler et al. 2023) | effective and acceptable safety (Stadler et al. 2023) | established use and need (Cholo et al. 2012; van Ingen 2024) | retained | constant | important in treatment of leprosy and tuberculosis |
| cloxacillin (penicillinase-resistant) | bacterial infections (not specified) | Access group: first-choice bone/joint infection, skin/soft tissue infections, second choice sepsis in neonates and children | specification | yes, a first choice in MSSA (Kaasch et al. 2015; Burdet et al. 2025) | effective in MSSA, acceptable safety (Kaasch et al. 2015; Bai et al. 2025) | public health need and regularly used in susceptible infections (Bru and Garraffo 2012) | retained | constant | advantage of efficacy and narrow spectrum, availability of suitable alternatives (e.g. cefazolin) (Bru and Garraffo 2012; Bai et al. 2015) |
| dapsone | leprosy | leprosy | no | yes (Lovell et al. 2024; Dev et al. 2025) | effective, but adverse effects require monitoring (Hilder and Lockwood 2020; Dev et al. 2025) | use in control programs (Krismawati et al. 2025; Dev et al. 2025) | retained | constant | effective and established use (Lovell et al. 2024; Dev et al. 2025) |
| diethylcarbamazine | filarial infections | filarial infections | no | yes (CDC 2024a; Ramharter et al. 2025 | effective, but caution of loa loa co-endemic because severe reactions (Adinarayanan et al. 2007; Stanley 2010; Herrick et al. 2017) | used in national programs (Honorato et al. 2016; Ramharter et al. 2025 | retained | constant | effective but safety concerns in loa loa areas (Herrick et al. 2017; |
| diloxanide | amoebic infections | amoebic and giardia infections | addition of giardiasis | yes (Inshutiyimana et al. 2024) | effective and good safety (Thomas and Garg 2007) | essential in niche (Inshutiyimana et al. 2024) | retained | constant | effective, complements systemic antibacterial drugs (Legese et al. 2021) |
| doxycycline | bacterial infections (not specified) | Access group: first-choice cholera and STI due to C. trachomatis, second-choice community-aquired pneumonia and exacerbations of COPD; malaria (chemoprophylaxis) | specification | yes (Pearson et al. 2025) | effective, acceptable adverse effects, bacterial resistance rates acceptable (Chan et al. 2023; de Macedo et al. 2023; Roster and Grad 2024; Holmes et al. 2009) | widely used in bacterial infections, public health need (Pearson et al. 2025) | retained | constant | covers broad range of infections (Holmes et al. 2009) |
| ethambutol | tuberculosis | tuberculosis | no | yes (Drew and Sterling 2025; van der Walt et al. 2020) | effective but possibility of serious adverse effects (e.g. optic neuropathy) (Prasad et al. 2021; Perumal et al. 2020; Sudhakar et al. 2025) | widely used and part of treatment regimens (Drew and Stelring 2025; van der Walt et al 2020) | retained | constant | important part of therapy in FDC (Drew and Sterling 2025) |
| ***Table S1 (continued)*** | | | | | | | | | |
| ***Retained drugs*** | ***Indication EML 1977*** | ***Indication EML 2025*** | ***Change*** | ***Recommendation in guidelines*** | ***Effectiveness and safety*** | ***Public health need*** | ***Suggestion*** | ***Clinical use*** | ***Explanation*** |
| gentamicin | bacterial infections (not specified) | Access group: acute bacterial meningitis in neonates and severe CAP and complicated intraabdominal infections and complicated severe acute malnutrition and sepsis in neonates and children, second choice gonorrhoea and surigcal prophylaxis, ophthalmological infections (bacterial conjunctivitis and keratitis) | specification | yes (La Vecchia et al. 2025; McGlona and Cranswick 2008) | yet effective but toxicity issues (e.g. oto- and nephrotoxicity) and increasing bacterial resistance (Fenner 2019; Modi et al. 1998; Balakumar et al. 2010; Best et al. 2011; Sparo et al. 2018) | established use in severe infections (La Vecchia et al. 2025; McGlone and Cranswick 2008) | retained | constant | important for spec. severe infections (particularly in children) (McGlone and Cranswick 2008; de Vries Schultink et al. 2021) |
| isoniazid | tuberculosis | tuberculosis, HIV-related opportunistic infections in FDC | addition of HIV-related infections | yes (Gegio et al. 2017; Zhou et al. 2024) | effective but risk of adverse effects (e.g. hepatotoxicity, peripheral neuropathy) (Sankar et al. 2024; Zhou et al. 2024; Sodré-Alves et al. 2024 | use in treatment regimes, public health importance (Zhou et al. 2024; Sodré-Alves et al. 2024) | retained | constant | public health need, efficacy, part of treatment regimens (Sodré-Alves et al. 2024; Zhou et al. 2024) |
| mebendazole | helminthic infections | helminthic infections (intestinal, cysticidal) | specification | yes (Silber et al. 2017; Levecke et al. 2014) | effective and acceptable safety (Silber et al. 2017; Levecke et al. 2014; Bekele et al. 2024) | need in endemic areas and use national public health programs (Minsalud 2022) | retained | constant | cost-effective and public health need (Mindsalud 2022; Bekele et al. 2024) |
| metronidazole | amoebic infections | Access group: first choice C. difficile infection, complicated intraabdominal infections, necrotizing fasciitis, surgical prophylaxis, trichomoniasis | addition of several bacterial infections and specification | partly, because not anymore recommended for C. difficile (NICE 2021; van Prehn et al. 2021) | acceptable safety but increasing baacterial resistance with depleted efficacy (Giles and Roberts 2022; Galante et al. 2025) | widely used depending on indication (Hernández Ceruelos et al. 2019) | retained | constant | broad usage but no recommendation for C. difficile anymore (van Prehn et al. 2021; Hernández Ceruelos et al. 2019) |
| niclosamide | helminthic infections | helminthic infections (intestinal) | specification | yes (Biersack 2024; Pan American Health Organization 2021) | effective and acceptable safety (Haby et al. 2020 | need in endemic areas and in national public health programs (Chen et al. 2017; Haby et al. 2020; Pan American Health Organization 2021) | retained | constant | effective and public health need |
| nifurtimox | trypanosomial infection | trypanosomial infection (2nd stage African) | specification | yes (Kuemmerle et al. 2021; Hidalgo et al. 2021) | effective and acceptable safety (Hidalgo et al. 2021; Falk et al. 2022; Crespillo-Andújar et al. 2018) | important in programs and endemic areas (Kuemmerle et al. 2021; Lejon et al. 2025) | retained | constant | key component in treatment regimes and public health programs (Hall et al. 2011; Falk et al. 2022; Lejon et al. 2025) |
| nystatin | fungal infections | fungal infections | no | effective in topical fungal infections but not in systemic use (Sousa et al. 2023; Pappas et al. 2015) | effective and safe in local infections (Rai et al. 2022; Lyu et al. 2016) | commonly used in primary health care (Chu et al. 2008; Rai et al. 2022) | retained | constant | safe and effective in superficial fungal infections, important for basic care (Chu et al. 2008; Rai et al. 2022) |
| pentamidine | trypanosomal and leishmaniac infections | malaria, pneumocystosis, trypanosomiasis (1st stage African) | specification | yes (Lindner et al. 2025; Rastegar-Pouyani et al. 2025) | effective, but toxicity and severe adverse effects require caution and limit use (Hatzl et al. 2025; O'Brien et al. 1997; Bean and Wareham 2017) | important treatment, public health need (Bean and Wareham 2017; Rastegar-Pouyani et al. 2025) | retained | constant | efficacy, severeness of conditions justifies use despite adverse effects, public health need (Hatzl et al. 2025; Lindner et al. 2025) |
| phenoxymethylpenicillin | bacterial infections | Access group: first-choice CAP, pharyngitis, progressive apical dental abscess | specification | yes (NHS 2022a; NICE 2018) | effective and acceptable safety (Skarpeid and Hoye 2018; Cronberg et al. 2025) | widely used in primary care (Skarpeid and Hoye 2018) | retained | constant | narrow-spectrum first-line for specified indications (Skarpeid and Hoye 2018) |
| primaquine | malaria | malaria (curative) | no | yes (Ashley et al. 2014; Yilma et al. 2025 | effective but adverse effects (e.g. haemolysis in G6PD deficiency) requires caution (Ashley et al. 2014; Yilma et al. 2025 | essential in control programs (Kaewkungal et al. 2024; Yilma et al. 2025) | retained | constant | effective and used in treatment regimens (Kaewkungal et al. 2024; Yilma et al. 2025) |
| pyrimethamine | malaria | malaria (curative, chemoprevention in FDC), pneumocystosis, antitoxoplasmosis | specification | yes (WHO 2022; CDC 2024c; HIVinfo NIH 2025; Masserey et al. 2025) | effective, manageable adverse events (haematologic toxicity) with folic acid and monitoring, increasing bacterial resistance requires use of FDCs (Bell et al. 2008; Mombo-Ngoma et al. 2025; von Eijk et al. 2025) | essential in toxoplasmosis and malaria FDC (Mombo-Ngoma et al. 2025; WHO 2022; Audibert et al. 2025; Masserey et al. 2025) | retained | constant | effective and widely used, part of treatment regimens in FDC (Mombo-Ngoma et al. 2025; WHO 2022; Audibert et al. 2025) |
| cyclophosphamide | cancer (not specified) | cytotoxic (ALL, anaplastic large cell lymphoma, Burkitt lymphoma, CLL, diffuse large B-cell lymphoma, early stage breast cancer, Ewing sarcoma, follicular lymphoma, Hodgkin lymphoma, low-grade glioma, metastatic breast cancer, multiple myeloma, nephroblastoma, rhabdomyosarcoma) | specification | yes (IWMF 2021; Sammaritano et al. 2025) | effective but caution about adverse effects (e.g. myelosuppression, haermorrhagic cystitis), positive benefit-risk-ratio (Brummaier et al. 2013; Kedia and Malaviya 2025; Alrefaei et al. 2022; Gephart et al. 2025) | important part of several treatment regimens globally (Kedia and Malaviya 2025; Gephart et al. 2025) | retained | constant | broad-spectrum use in cancer and rheumatologic conditions |
| doxorubicin | cancer (not specified) | cytotoxic (ALL, anaplastic large cell lymphoma, Burkitt lymphoma, diffuse large B-cell lymphome, early stage breast cancer, Ewing sarcoma, follicular lymphoma, Hodgkin lymphoma, metastatic breast cancer, multiple myeloma, nephroblastoma, osteosarcoma) | specification | yes (ONS 2025) | effective but dose-dependent cardiotoxicity limits use (ONS 2025; Rawat et al. 2021 | widely used in cancer treatment (Kciuk et al. 2023; Sritharan and Sivalingam 2021) | retained | constant | major antrhacycline used across oncology regimens (Kciuk et al. 2023) |
| fluorouracil | cancer (not specified) | cytotoxic (early stage breast/colon/rectal cancer, metastatic colorectal cancer, nasopharyngeal cancer, kaposi sarcoma as pegylated liposomal) | specification | yes (BC Cancer 2023; Ghafouri-Fard etal. 2021) | effective but severe adverse effects possible (e.g. cardiotoxicity, ocular toxicity), positive benefit-risk ratio (BC Cancer 2023; Ghafouri-Fard et al. 2021; Negarandeh et al. 2020; Park et al. 2025) | widely used in oncology (Longley et al. 2003; BC Cancer 2023) | retained | constant | important drug for colorectal and other cancers (Longley et al. 2003) |
| ***Table S1 (continued)*** | | | | | | | | | |
| ***Retained drugs*** | ***Indication EML 1977*** | ***Indication EML 2025*** | ***Change*** | ***Recommendation in guidelines*** | ***Effectiveness and safety*** | ***Public health need*** | ***Suggestion*** | ***Clnical use*** | ***Explanation*** |
| vincristine | cancer (not specified) | cytotoxic (ALL, acute promyelocytic leukaemia, Burkitt lymphoma, diffuse large B-cell lymphoma, Ewing sarcoma, follicular lymphoma, Hodgkin lymphoma, Kaposi sarcoma, Langerhans cell histiocytosis, low-grade glioma, nephroblastoma, retinoblastoma, rhabdomyosarcoma) | specification | yes (Dhyani eta l. 2022) | beneficial outcomes through treatment, but neurotoxicity and myelosuppression limits dosage (Barnett et al. 2022; Škubník et al. 2021; Garg et al. 2025) | widely used, particularly in paediatric oncology (Barnett et al. 2022; Garg et al. 2025) | retained | constant | important part of standard regimens in many cancer treatments (Tsakatikas et al. 2021; Škubník et al. 2021) |
| ferrous salt | anaemia | anaemia | no | iron deficiency anaemia (NHS 2022b) | effective but common slight adverse effects (GI disturbance) (Canadian Agency for Drugs and Technologies in Health 2016; Tolkien et al. 2015; Kalman et al. 2025) | global need and common condition (Santiago 2012; Global Burden of Disease Collaborative Network 2023) | retained | constant | basic care, universal need |
| folic acid | anaemia | anaemia | no | megaloblastic anaemia due to folate deficiency (Baddam et al. 2025) | effective and safe (Gomes et al. 2025; Palika et al. 2022 | global need, widely used as nutritional supplement (Baddam et al. 2025) | retained | constant | important in ananemia treatment, also used in early pregnancy for prevention of neural tube defects (Rockenbach et al. 2025; |
| hydrochlorothiazide | hypertension, diuretic | hypertension, heart failure, prevention of atheroslerotic CD, diuretic | addition of cardiovascular-related indications | first-line in hypertension, benefit in heart failure and ASCVD (Jones et al. 2025) | effective, acceptable safety but considerable adverse effects, beneficial outcomes but not recommended as initial hypertension treatment (Rosendorff et al. 2011; Morales-Olivas 2024; Reinhart et al. 2023; Natov and Schwartz 2025) | widely used (Natov and Schwartz 2025) | retained | constant | broad and common usage in cardiovascular conditions, evidence of efficacy but discussion about first-line treatments |
| isosorbide dinitrate | hypertension | angina | change of indication | relief of acute anginal symptoms (Rao et al. 2025) | effective but issue of tolerance development, only short-term treatment (Rao et al. 2025; Soman and Vijayaraghavan 2017) | widely used in angina, importance in treatment of heart failure (Cole et al. 2011; Soman and Vijayaraghavan 2017) | retained | constant | appropriate, effective and rapid treatment in acute angina |
| lidocaine | arrhythmia, local anaesthetic | arrhythmia, local anaesthetic | no | treatment of ventricular arrhythmia and local anaesthetic (Kido 2025; Güler et al. 2023) | effective and acceptable safety but systemic toxicity at high doses (Güler et al. 2023; Bahar and Yoon 2021; Schwenk et al. 2025) | widely used in emergency and procedural settings (Güler et al. 2023; Bahar and Yoon 2021) | retained | constant | dual role in emergency sitations and basic anaesthesia |
| methyldopa | hypertension | hypertension | no | first-line in pregnancy, second-line in other hypertension treatments (De Backer et al. 2025) | beneficial, effective and safe in hypertension during pregnancy (Hoeltzenbein et al. 2017; Easterling et al. 2019; Mah et al. 2022) | public health need and common use in pregnancy (Easterling et al. 2019) | retained | constant | important for management of hypertension in pregnancy |
| phytomenadione | anticoagulant antagonist | anticoagulant antagonist | no | antidote for vitamin-K-dependent anticoagulants (Bekka and Liakoni 2024) | effective and safe, live-saving in bleeding events or overdosing of anticoagulants (Schulman and Furie 2015; Shields et al. 2001) | universal need and utility (Bekka and Liakoni 2024) | retained | constant | need in emergency and basic care |
| propanolol | hypertension | migraine prophylaxis | change of indication and usage field | prophylaxis of migraine, not for hypertension (Qaseem et al. 2025; Versijpt et al. 2024; De Caterina and Leone 2010) | effective, well-known safety profile (bradycardia, bronchospasm caution) (Versijpt et al. 2024; Sprenger et al. 2018; Linde and Rossnagel 2017) | most commonly used drug in migraine prophylaxis (Linde and Rossnagel 2017) | retained | constant | effective and widely used |
| protamine sulfate | anticoagulant antagonist | anticoagulant antagonist | no | standard antidot for heparin (Rao et al. 2025) | effective in heparin neutralization, rare anaphylaxis risk, overall favourable when indicated (Silva et al. 2026; Kara et al. 2025; Oda et al. 2025) | essential in emergency and interventional settings when heparin reversal is required (Kara et al. 2025) | retained | constant | indispensible for rapid heparin anticoagulation reversal |
| bupivacaine | local anaesthesia | local anaesthesia | no | yes (Rote Liste 2022) | effective and acceptable safety, cardiotoxicity risk at high intravascular doses (Fettiplace et al. 2025; Velioglu et al. 2020; Ryu et al. 2007) | essential in anaesthesia practices globally (Paganelli and Popescu 2015; Velioglu et al. 2020) | retained | constant | long-acting local anaesthetic of high clinical utility, use with safety precautions |
| diazepam | psychotherapeutic drugs, seizures | anxiety disorders, seizures | specification | status epilepticus, second choice/short-term treatment of anxiety disorders(Fagan and Baldwin 2023; Kienitz et al. 2022) | effective in acute seizure control and anxiolysis, dependency risk limit chronic use (Fagan and Baldwin 2023; Manno 2011; Kienitz et al. 2022; Divljakovic et al. 2012) | high in emergency and mental health settings, important for seizure emergencies (Bindel and Seifert 2025d; Manno 2011; Kienitz et al. 2022) | retained | constant | key emergency benzodiazepine for acute indications, limited chronic usage |
| ethosuximide | seizures | seizures | no | first-line initial treatment childhood absence epilepsy (Brigo et al. 2021 | evidence for efficacy in absence epilepsy, relatively favourable tolerability (Brigo et al. 2021; Glauser et al. 2010) | essential in pediatric neurology | retained | constant | specific utility for absence seizures |
| haloperidol | psychotherapeutic drugs | psychotic disorders | specification | psychotic disorders and acute severe agitation/delirium (Markota et al. 2024; Currier and Trenton 2002; Jiang and Gunther 2025) | effective antipsychotic for acute psychosis and agitation (particularly "positive symptoms"), extrapyramidal adverse effects require monitoring, availability of treatment alternatives (Dold et al. 2015; Sykes et al. 2017; Markota et al. 2024; Currier and Trenton 2002; Jiang and Gunther 2025) | widely used in many settings, including emergency psychiatry and general wards for delirium/agitation (Dold et al. 2015) | retained | constant | effective and inexpensive, monitoring for EPS |
| ibuprofen | pain, anti-inflammatory | pain, anti-inflammatory, acute migraine attack | addition of indication for acute migraine attack | yes (Amaechi et al. 2021; Rabbie et al. 2013;Mayans and Walling 2018; de'Angelis et al. 2025) | effective, favourable risk-benefit in acute settings, adverse effects at high doses and long-term use need consideration (Rabbie et al. 2013; Ainyanbhor et al. 2025; Jyothirmai et al. 2022) | high global usage for analgesia, commonly available OTC (Ainyanbhor et al. 2025; de'Angelis et al. 2025) | retained | constant | versatile use with established efficacy for short-term treatment |
| lithium carbonate | psychotherapeutic drugs | bipolar disorders | specification | gold-standard mood stabilizer for bipolar disorder, prevention of relapse and suicide risk reduction (NICE 2025a; Bindel and Seifert 2025a) | effective, major benefit in relapse prevention, narrow therapeutic index, organ monitoring need (Airainer and Seifert 2024; Post and Rybakowski 2024; Boswell et al. 2019; Bindel and Seifert 2025b) | essential in management of bipolar disorder, although widely underused (Post and Rybakowski 2024; Bindel and Seifert 2025b) | retained | constant | unique efficacy in bipolar disorder and suicide prevention, monitoring necessary (Mouawad 2022) |
| morphine | pain | pain | no | first-line opioid for moderate-severe pain and palliative management (WHO 2018a; NICE 2016b) | efficacy, favourable treatment despite adverse effects (Paul et al. 2024; Flemming 2010; Maier et al. 2002) | essential analgesic in hospitals and palliative care, most commonly used for severe pain (WHO 2025c) | retained | constant | cornerstone opioid analgesic, part of the WHO Analgesic Ladder (WHO 2018a) |
| nitrous oxide | anaesthesia | anaesthesia | no | adjunct general anaesthetic, sedative in interventional procedures (Rollins et al. 2025; AAPD 2023) | effective mild analgesic and sedative, safety considerations such as hypoxia risk, environmental concerns, need of infrastructure for safe delivery (Khinda et al. 2023; Rollins et al. 2025; Zaloum et al. 2025) | widely used in procedural sedation (Khinda et al. 2023) | retained | constant | useful adjunct with rapid onset and recovery, growing concern about use as "party drug" (De Morais et al. 2024; Zaloum et al. 2025) |
| ***Table S1 (continued)*** | | | | | | | | | |
| ***Retained drugs*** | ***Indication EML 1977*** | ***Indication EML 2025*** | ***Change*** | ***Recommendation in guidelines*** | ***Effectiveness and safety*** | ***Public health need*** | ***Suggestion*** | ***Clinical use*** | ***Explanation*** |
| paracetamol | pain | pain, acute migraine attack | addition of indication for acute migraine attack | first-line analgesic in mild-moderate pain (NSW Government 2024; Freo et al. 2021) | effective in mild pain and in multimodal analgesia, effective and favourable adverse effects but hepatotoxicity risk in overdose (Freo et al. 2021; Derry and Moore 2013; Freo 2022; Rotundo and Pyrsopoulos 2020) | widely used, high utility worldwide (Ayoub 2021) | retained | constant | well-establlished and favourable analegesic in primary care and in multimodal concepts (e.g. WHO Analgesic Ladder) (Alchin et al. 2022; Freo 2022) |
| tetracaine | local anaesthesia | local anaesthesia | no | local anaesthesia (topical/ocular/local) (Green et al. 2024; Stavleu et al. 2025) | effective topical/local anesthetic for ophthalmic and superficial procedures, safe but adverse effects in long term use, other alternatives like EMLa may be more suitable in some settings (Weiser et al. 2024; Alster 2013; Shipman et al. 2021; Kim et al. 2012; Stavleu et al. 2025; Giordano et al. 2015) | important in ophthalmology and minor procedures (Kumar et al. 2015) | retained | constant | basic care and minor interventions |
| barium sulfate | radiocontrast media | radiocontrast media | no | inert radiopaque contrast for GI imaging (ACR 2025) | effective and low rate of adverse effects, contraindicated if risk of aspiration/perforation (Kang et al. 2023; Najjar 2024; Zaccarini e tal. 2022) | essential for diagnostic radiology in many settings (Najjar 2024) | retained | constant | standard GI radiographic contrast |
| oral rehydration salts | diarrhoea, oral rehydration | diarrhoea, oral rehydration | no | acute diarrhoea, prevention of dehydration (WHO 2006b; Government of Canada 2024a) | effective and safe, mortality reduction in severe diarrhoea (Munos et al. 2010; Mahfuz et al. 2024) | widely used in global child-health programs and disaster response (UNICEF 2025) | retained | constant | life-saving basic intervention, universal need |
| fludrocortisone | adrenal hormone substitution | adrenal hormone substitution | no | mineralocorticoid deficiency (Bornstein et al. 2016) | effective, monitoring of dosing and adverse effects required (Bornstein et al. 2016; Løvås and Husebye 2008) | niche, essential for addisons disease (Bornstein et al. 2016) | retained | constant | important hormonal replacement |
| potassium iodide | thyroid hormone antagonist | thyroid hormone antagonist, fungal infections | addition of indication for antiinfective treatment | pre-operative in hyperthyreoidism (Graves' disease), nuclear emergencies, cutaneus sporotrichosis (Sharma et al. 2022; da Silva Lopes et al. 2024; Kahaly et al. 2018) | efficacy for thyroid blocking, iodine intolerance/allergy and thyroid dysfunction risks require caution, low evidence for efficacy in fungal infections (Xue et al. 2022; Xue and Li 2009; Randle et al. 2018; Spallek et al. 2012; Fujikawa and Okamura 2024) | nuclear emergency preparedness, importance in thyreoidectomie for Graves' disease (Randle et al. 2018; da Silva Lopes et al. 2024) | retained | constant | essential in thyroid blockade |
| propylthiouracil | thyroid hormone antagonist | thyroid hormone antagonist | no | hyperthyroidism particularly in first trimester of pregnancy (or contraindication methimazole) (Kahaly et al. 2018) | effective but rare severe adverse effects (hepatotoxicity) (Miao et al. 2022; He et al. 2024) | alternative to methimazole and important option in pregnancy and contraindications (Kahaly et al. 2018; Wiessel 2010) | retained | constant | alternative for methimazole and first-line in pregnancy |
| anti-D immunoglobulin | immunization (not specified) | immunization (not specified) | no | prevention of hemolytic disease of the fetus, treatment of immune thrombocytopenia (Qureshi et al. 2014; Neunert et al. 2019) | evidence for efficacy and prevention of alloimmunization, mild adverse effects possible (Xi et al. 2020; Guo et al. 2018) | essential in perinatal programs and used in haematogy | retained | constant | lifesaving and safe preventative role in maternal–fetal medicine |
| BCG vaccine | vaccination | vaccination | no | prevention of severe paediatric tuberculosis (CDC 2025a) | protection against severe childhood tuberculosis but considered not effective in adults, generally mild adverse effects in neonates except in severe immunodeficiency (Okuno et al. 2022; Khandelia et al. 2023; Starshinova et al. 2024; Martinez et al. 2024) | essential vaccine in high-burden settings, programmatic importance in neonatal immunization schedules (DZK 2025; Khandelia et al. 2023; Starshinova et al. 2024) | retained | constant | routine neonatal vaccine in regions with high TB burden |
| diphtheria antitoxin | immunization (not specified) | immunization (not specified) | no | standard antitoxin therapy for diphtheria (UK Health Security Agency 2025) | benefit in toxin neutralization, usually mild adverse effects but risk of hypersensitivity, no preventive use (UK Health Security Agency 2025; Eisenberg et al. 2021; Truelove 2019) | important in treatment of confirmed or suspected diphtheria infection (UK Health Security Agency 2025) | retained | constant | persistent global health threat (Pérez and Brisse 2025) |
| rabies vaccine | vaccination | vaccination | no | post-exposure prophylaxis and pre-exposure in high-risk groups (WHO 2018b; CDC 2025b) | effective in both prophylaxis and post-exposure treatment, safe, well tolerated, life-saving (Fotedar and Ravish 2025; Ahmad et al. 2022; CDC 2025b) | important in endemic regions in untreated fatal infection (Ahmad et al. 2022; WHO 2018b) | retained | constant | effective and safe prophylaxis/ treatment for otherwise fatal disease |
| tetanus vaccine | vaccination | vaccination | no | routine infant, maternal and wound prophylaxis vaccination (WHO 2019b) | effective and safe, strong mortality-reduction (Pollard and Bijker 2020; Lago-Deibe et al. 2022) | essential vaccination (Slifka et al. 2025; CDC 2025c; WHO 2019b) | retained | constant | universal need in public-health immunization (CDC 2025c) |
| ethinylestradiol + norethisterone | contraception | contraception | no | oral contraception (FRSH 2019) | effective in prevention of pregnancy, adverse effects well known (e.g. VTE) (FDA 2017; Archer et al. 2013) | major role in reproductive health (FRSH 2019) | retained | constant | basic care, effective and widely used contraception |
| oxytocin | oxytocics | oxytocics | no | labour induction and first choice in portpartum haemorrhage (Papageorghiou et al. 2025; Kehl et al. 2021) | effective and safe (mild adverse effects), only indicated in advanced labour (Kehl et al. 2021; Parry Smith et al. 2020; Van Netten et al. 2025) | widely used and needed globally, leading cause for maternal mortaility (Jones et al. 2023) | retained | constant | gold standard uterotonic, more effective and less adverse effects than alternatives (Parry Smith et al. 2020; Van Netten et al. 2025) |
| intraperitoneal dialysis solution | chronic kidney disease | chronic kidney disease | no | peritoneal dialysis in chronic kidney failure (KDIGO 2024) | effective, comparably safe, convenient and improved life quality for patients but switch to hemodialysis in the disease course often necessary (Jung et al. 2019; Sharma et al. 2024; Reimann and Tomlins 2023; Chaudhary 2011) | essential in the beginning of dialysis-requiring chronic kidney disease, or when haemodialysis access limited (KDIGO 2024) | retained | constant | important therapy option in many low-resource settings, improved life quality and convenience for patients in comparison to hemodialysis |
| ascorbic acid | nutritional supplement | nutritional supplement | no | vitamin C deficiency and prevention of scurvy in malnutrition (Berger et al. 2024) | effective in correction of deficiency, acceptabe safety, no proven effect in unspecific usage (Golder et al. 2025; Lykkesfeldt 2020; Gandhi et al. 2023; Decke and Seifert 2025) | relevant in malnutrition and chronic diseases (Golder et al. 2025; Trangkanont et al. 2025) | retained | constant | effective and important in deficiency |
| ergocalciferol | nutritional supplement | nutritional supplement | no | vitamin D deficiency and prevention of osteoporosis (NOGG 2024; NHS 2025c) | effective in defificiency correction and benefit in osteoporosis, low rate of adverse effects but risk of toxicity in overdose (Giustina et al. 2024; NHS 2025c; Demay et al. 2024; Prince et al. 2008; Pickering et al. 2025) | relevant in deficiency/malnutrition and osteoporosis treatment | retained | constant | safe and effective in compensation of deficiency and prevention of osteoporosis-related complications (Prince et al. 2008; NHS 2025c) |
| iodine | nutritional supplement | antiseptic, nutritional supplement | addition as an antiseptic | iodine deficiency, alternative topical antisepsic (Babalska et al. 2021; Berger et al. 2024) | effective as antiseptic and in deficiency correction, improval of thyroid dysfunctions and cognitive development of iodine-deficient children, mild adverse effects but risk of iodine excess, availability of more favourable antiseptics (Zimmermann et al. 2006; Conner-Kerr 2014; Babalska et al. 2021; Sohn et al. 2024; Gordon et al. 2009) | broad in low-resource surgical and obstretic settings and compensation of deficiency (Conner-Kerr 2014; Berger et al. 2024) | retained | constant | dual role, cost-effective, easy administration, effective and safe |
| ***Table S1 (continued)*** | | | | | | | | | |
| ***Retained drugs*** | ***Indication EML 1977*** | ***Indication EML 2025*** | ***Change*** | ***Recommendation in guidelines*** | ***Effectiveness and safety*** | ***Public health need*** | ***Suggestion*** | ***Clinical use*** | ***Explanation*** |
| pyridoxine | nutritional supplement | nutritional supplement | no | vitamin B6 deficiency, isoniazid prophylaxis (WHO 2024b; Berger et al. 2024) | effective but toxicity in excessive dosage (e.g. sensory neuropathy, ataxia) (Sousou et al. 2023; Berger et al. 2024; WHO 2024b) | widely used globally, prevention of complications in cancer treatment, high prevalence of deficiency in alcohol use disorder (Sousou et al. 2023; Berger et al. 2023) | retained | constant | established role but risk of adverse effects in overdosing |
| retinol | nutritional supplement | nutritional supplement | no | vitamin A deficiency (Wirth et al. 2017; Berger et al. 2024) | effective and strong mortality reduction in deficient populations, safe but adverse effects of overdosing (Alouache 2025; Sattar et al. 2012; Watson et al. 2018; Boniphace et al. 2007) | widely used in malnutrition programs for children (Wirth et al. 2017; Bruins and Kraemer 2013) | retained | constant | essential in malnutrition programs, deficiency with adverse outcomes (Alouache 2025) |
| acetazolamide | opthalmology (systemic, not specified) | glaucoma | specification | acute intraocular pressure reduction (Chak et al. 2015) | effective, rapid onset, acceptable safety but rare serious adverse effects (e.g. anaphylaxis, agranulocytosis, aplastic anaemia), no long-term use (Chak et al. 2015; Gulati and Aref 2021; Lee et al. 2025b) | ermergency opthalmology | retained | constant | important in emergency situations, favouarlbe benefit-risk ratio, acute treatment but no long-term use (Gulati and Aref 2021) |
| pyridostigmine | peripheral muscle relaxant (not specified) | myastenia gravis, muscle relaxant | specification | first-line long-term myasthenia gravis therapy (Jacob et al. 2025) | effective, symptom contral and use in long-term therapy (Remijn-Nelissen et al. 2022; Urban et al. 2018; Maggi and Mantegazza 2011) | widely used and first-line for mystenia gravis, high burden of disease (Remijn-Nelissen et al. 2022; Lehnerer et al. 2021) | retained | constant | important role and effective in therapy of myastenia gravis |
| ephedrine | asthma | local anaesthetic | change of indication | use in anaesthesia for hypotension (Kinsella et al. 2017) | effective vasopressor in acute settings with acceptable safety, no longer used for asthma because of adverse effects and availability of more suitable alternatives (Park et al. 2020; Kinsella et al. 2017) | important in surgical interventions (Lonjaret et al. 2014) | retained | constant | shift in usage, asthma indication obsolete, anaesthesia use dominates |
| glucose | solutions correcting water, electrolyte, and acid-base disturbances | solutions correcting water, electrolyte, and acid-base disturbances | no | hypoglycaemia, fluid replacement (NHS 2025a, 2024a) | effective and safe (Verma et al. 2024; Liu et al. 2022) | need in emergency care (JBDS-IP 2023) | retained | constant | important and widely used in basic and emergency care |
| potassium chloride | solutions correcting water, electrolyte, and acid-base disturbances | solutions correcting water, electrolyte, and acid-base disturbances | no | hypokalemia (FDA 2014) | effective but need of monitoring during administration (Hirman and Chong 2024; Tinawi 2020; MSF 2025b; FDA 2022a) | yes (Kardalas et al. 2018; Tinawi 2020) | retained | constant | need in emergency care, effective and indispensable although high-risk medication (severe adverse events because of dosing errors) (FDA 2022a; Kothari et al. 2012) |
| sodium chloride | solutions correcting water, electrolyte, and acid-base disturbances | solutions correcting water, electrolyte, and acid-base disturbances | no | hypernatraemia, fluid replacement (Miller et al. 2023; RCH 2020) | effective and safe (Shamim et al. 2015, RCH 2020) | impotant iv crystalloid, need in emergency care (Miller et al. 2023) | retained | constant | important component in emergency care in treatment of dehydration and shock |
| sodium lactate | solutions correcting water, electrolyte, and acid-base disturbances | solutions correcting water, electrolyte, and acid-base disturbances | no | fluid replacement, metabolic acidosis (NHS Scotland 2020) | effective and safe (Ichai et al. 2014; Honore et al. 2019; Omar Ibrahim et al. 2025) | important in emergency and critical care (Eli et al. 2024) | retained | constant | broad compatibility in resuscitation, used in protocols |
| water | solutions correcting water, electrolyte, and acid-base disturbances | solutions correcting water, electrolyte, and acid-base disturbances | no | rehydration, drug solvent (MSF 2025a; EMA 2020) | effective for hydrophilic substances (Sigma-Aldrich 2025) | essential for hydration and dilution (Agostoni et al. 2011; Popkin et al. 2011) | retained | constant | universal need across all clinical settings, foundation of rehydration therapies |
| amikacin | bacterial infections (not specified) | Access group: first-choice high-risk febrile neutropenia, severe pyeloneprhritis/prostatitis, second choice sepsis in children; tuberculosis | specification | yes (UKU 2023; NHS 2023) | TDM necessary, moderate efficacy because of increasing bacterial resistance (Luo et al. 2023; UKU 2023; NHS 2023) | still important but availability of alternatives (aminoglykosides) (NHS 2023; Ramirez and Tolmasky 2017; Kim et al. 2017) | retained | declining | need for several options in the treatment of severe Gram-negative infections and tuberculosis |
| amphotericin B | fungal infections | fungal infections, leishmaniasis | addition of leishmaniasis | yes (Cavassin et al. 2021; Rudolph 2024; Kumari et al. 2022) | effcient and low resistance rates, but high rate of adverse events and need of monitoring during administration (Cavassin et al. 2021; Rudolph 2024) | important, ongoing development of alternatives (Cavassin et al. 2021; Akinosoglou et al. 2024) | retained | declining | widely used and effective but caution about adverse effects (Cavassin et al. 2021; Maertens et al. 2022) |
| sodium stibogluconate | leishmaniasis | leishmaniasis | no | yes, but alternatives sometimes preferred (Murray et al. 2012; Silva et al. 2021) | effective in combination therapy but toxicity and regions-specific resistance issues (van Henten et al. 2023; Scholar 2007; Musa et al. 2023; Silva et al. 2021) | widely used in some regions, recommended in guidelines (Musa et al. 2023; Silva et al. 2021) | retained | declining | toxicity issues and region-specific resistance, comparable alternatives available (Cesur et al. 2002; Solomon et al. 2024; Murray et al. 2012) |
| sulfadiazine | bacterial infections (not specified) | malaria (antipneumocystosis, antitoxoplasmosis) | narrowed and specified indication | only recommended for toxoplasmosis (RKI 2018; Dunay et al. 2018; CDC 2024c) | effective in combination therapy because reports of resistant pathogens, acceptable safety, availability of more favourable alternatives (Konstantinovic et al. 2019; Dunay et al. 2018; CDC 2024c; Konstantinovic et al. 2019) | niche use, part of recommended regime for toxoplasmosis but not in malaria, public health importance (Castano et al. 2022; Konstantinovic et al. 2019) | retained | declining | first-line treatment of toxoplasmosis in combination therapy (RKI 2018; Dunay et al. 2018) |
| sulfamethoxazole + trimethoprim | bacterial infections (not specified) | Access group: first choice lower UTIs, second choice acute invasive bacterial diarrhoea/dysentery; prevention of HIV-related opportunistic infections; malaria (antipneumocystosis and antitoxoplasmosis) | specification | yes (EAU 2025; MSF 2025d; Rote Liste 2025b) | effective in most primary-care settings but increasing bacterial resistance, acceptable safety, discussion about benefit in prevention of HIV in children (Assefa and Girmay 2024; Rote Liste 2025; Daniels et al. 2022; Bwakura-Dangarembizi et al. 2014; WHO 2025d; Alsaad et al. 2013) | used in a variety of infections (EAU 2025; MSF 2025d; Rote Liste 2025b) | retained | declining | multi-purpose in specified indications, effective, but problematic bacterial resistance rates and no uncertain evidence in benefit of HIV prevention (Rezaei-Tavirani et al. 2018; Assefa and Girmay 2024; WHO 2025d; Bindel and Seifert 2025c) |
| diazoxide | hypertension | hypoglycaemia | change of indication and usage field | first line for hypoglycaemia in congenital hyperinsulinism and insulinoma context, no use for hypertension (Newman-Lindsay et al. 2022; Chen et al. 2021) | effective but adverse effects (hirsutism, fluid retention, pulmonary hypertension) limits use (Newman-Lindsay et al. 2022; Laing et al. 2024) | niche use in endocrine care (Pasquini et al. 2025) | retained | declining | change of indication, no more used in hypertension treatment, only recommended drug for neonatal hypoglycemia (Chen et al. 2021) |
| hydralazine | hypertension | hypertension | no | use in pregnancy and in combination therapy for treatment-refractory hypertension (NICE 2023; Mech and Seifert 2025) | effective, but adverse effects (reflex tachycardia, lupus-like reaction, lower birthweight) limit use (Kandler et al. 2022; Mech and Seifert 2025; Antza et al. 2020) | declining use, importance in the treatment of hypertension in pregnancy (Countouris et al. 2025; Mech and Seifert 2025) | retained | declining | decreasing importance and indications, but still commonly used and recommended in pregnancy (Mech and Seifert 2025) |
| warfarin | anticoagulant | anticoagulant | no | first-line for atrial fibrillation with mechanical heart valves or rheumatic mitral stenosis (Chin and Doogue 2025) | effective and favourable risk-benefit in special patient populations, narrow therapeutic index and interactions require monitoring (INR), DOACs favoured in atrial fibrillation (Chin and Doogue 2025; Stuart et al. 2009; Dawwas et al. 2025) | declining because preference of DOAC, still widely used globally where DOACs unavailable or for specific indications (Tran et al. 2025; Lee et al. 2025a) | retained | declining | declining use and replacements by DOACs, important for special populations |
| ***Table S1 (continued)*** | | | | | | | | | |
| ***Retained drugs*** | ***Indication EML 1977*** | ***Indication EML 2025*** | ***Change*** | ***Recommendation in guidelines*** | ***Effectiveness and safety*** | ***Public health need*** | ***Suggestion*** | ***Clinical use*** | ***Explanation*** |
| amitriptyline | psychotherapeutic drugs | depressive disorders | specification | second-choice for depressive disorder (NSMRIs), SSRIs considered first-choice (Vos et al. 2021; NICE 2022c) | effective but other drug classes like SSRIs have a more favourable safety profile (Guaiana et al. 2007; Vos et al. 2021; Kovich et al. 2023; Kendrick et al. 2019) | frequently used (often off-label), alternative in therapy-refractory depressive disorders (Thour and Marwaha 2023) | retained | declining | declining use in depressive disorder but emerging importance in off-label use, important as alternative in depression treatment (Thour and Marwaha 2023; Vos et al. 2021) |
| carbamazepine | seizures | seizures | no | focal seizures (Nevitt et al. 2018) | effective but less well tolerated than newer antiseizure drugs, risk of rash/Stevens-Johnson and hematologic effects in rare cases (Bindel and Seifert 2025d; Nevitt et al. 2018; Montazerlotfelahi et al. 2024; Khoo et al. 2018; Hou et al. 2022) | important antiepileptic worldwide, especially where newer drugs unavailable (Bindel and Seifert 2025d; Beydoun et al. 2020) | retained | declining | well-established role and efficacy in specific seizure types |
| phenobarbital | seizures | seizures | no | status epilepticus and seizures (except absence) (Pressler et al. 2023; NICE 2025b) | effective, sedative effect, but discussion about cognitive/ developmental concerns with long-term use in children (Ul Haq et al. 2025; Trinka 2023; Wang et al. 2006; Brodie and Kwan 2012) | particularly used in LMICs because of cost-effectiveness (Brodie and Kwan 2012; Bankole et al. 2024) | retained | declining | effective, broad utility and availability, but higher rate of adverse effects than alternative treatments (Zhang et al. 2011) |
| phenytoin | seizures | seizures | no | second-line treatment for seizure control and status epilepticus (Nevitt et al. 2019; DeMott et al. 2020; ILAE 2020) | efficacy in seizure control, but narrow therapeutic index, common adverse effects (gingival hyperplasia, ataxia, hypersensitivity) and interactions (Nevitt et al. 2019; Gallop 2010; Watson 2014; Patocka et al. 2020; ILAE 2020) | decreasing use but widely used in LMICs (Bankole et al. 2024) | retained | declining | essential antiepileptic with broad use fields, alternative treatment option, widely available and cost-effective in low-resource settings |
| coal tar | psoriasis, topical | reduction of skin proliferation | no | adjunctive treatment for plaque psoriasis (DDG 2025; Elmets et al. 2021) | evidence for benefit in plaque psoriasis, safe but low convenience and patient acceptability limit use, other effective topical treatments available (e.g. calcipotriol) (Chan 2025; Slutsky 2010; Paghdal et al. 2009; Elmets et al. 2021; Singh et al. 2013) | adjunct therapy in dermatology,first-line treatment in many countries because of low cost and availability (Paghdal and Schwartz 2009) | retained | declining | low-cost and effective option despite cosmetic/acceptability limits and availability of alternatives, importance in low-resource settings |
| tuberculin, purified protein derivative (PPD) | diagnostic agents (not specified) | not specified | no | alternative tuberculosis screening to IGRA (Pinheiro et al. 2025; Sosa et al. 2019) | validated diagnostic tool though sensitivity/specificity lower than IGRA, inexpensive and accessible (Tobaiqi et al. 2025; Goletti et al. 2022) | common use in screening for tuberculosis in low-resource settings (Goletti et al. 2022) | retained | declining | simple, loct-cost TB screening tool, use in programs where IGRA not avaialbe |
| senna | laxative (gastrointestinal system) | laxative | no | stimulant laxative for short-term constipation, not recommended for long term use (Corsetti et al. 2024; Portalatin and Winstead 2012) | efficacy for constipation, acceptable safety but risk of drug interaction, long-term use not recommended because of tolerability development, availability of alternatives (Whorwell et al. 2024; MHRA 2020) | commonly used OTC (Rao and Brenner 2021) | retained | declining | inexpensive, effective short-term laxative but no long-term use, availability of alternatives (Corsetti et al. 2024) |
| ergometrine | oxytocics | oxytocics | no | second-choice for postpartum haemorrhage (Papageorghiou et al. 2025) | effective but risk of severe adverse effects, oxytocin preferred (Duvekot et al. 2025; Liabsuetrakul et al. 2018; Dind et al. 2023; Spencer and Lowe 2019) | decreasing use, potential advantage of combination with superior efficacy of combination therapy, leading cause for maternal mortality (Jones et al. 2023) | retained | declining | decreasing use but option for postpartum haemorrhage management and discussion about additive effect in combination with oxytocin (Gallos et al. 2025) |
| mannitol | diuretics | diuretics | no | reduction of intracranial pressure (ACS 2024) | effective osmotic agent but limited diuretic effect, adverse effects possible (e.g. acute kidney injury), alternatives available (hypertonic saline) (Kim et al. 2023b; Shawkat et al. 2012) | important and widely used in acute and trauma settings (Wang et al. 2015) | retained | declining | important in emergency settings |
| suxamethonium | peripheral muscle relaxant (not specified) | muscle relaxant | no | first-line for rapid sequence induction (Ahmad et al. 2025) | effective, rapid onset, reliable paralysis, adverse effects acceptable but require caution (e.g. pulmonary complications, hyperkalemia), alternative is rocuronium (less effective but safer and antidote available) (Ahmad et al. 2025; Gupta and Mishra 2021; Hovgaard and Juhl-Olsen 2021) | essential in airway management in anaesthesia | retained | declining | need in emergency treatment, unique in speed of onset but increasingly replaced by alternatives like rocuronium and sugammadex (safer but less rapid onset) (Fawcett 2019) |
| codeine | antitussive, diarrhoea | analgesics (not specified) | no | symptomatic and short-term relief of cough in adults, not recommended for children (DGP 2025; BfArM 2015) | effective in symptomatic relief but risk of adverse effects (respiratory depression) particularly in variable metabolism, contraindicated for children and in long-term use, analgesic effect not proven (Therapeutic Goods Administration 2016; Shaheed et al. 2016; Ćelić et al. 2020; Medicines and Healthcare products Regulatory Agency 2014; Tay and Roberts 2018) | declining in many regions, widely used for cough suppression, concerns about misuse (Shaheed et al. 2016; Tay and Roberts 2018; Van Hout and Norman 2016) | retained | declining | effective in cough suppression but safety concerns, inconclusive evidence about analgesic efficacy (Ćelić et al. 2020; Medicines and Healthcare products Regulatory Agency 2014) |
| benzylpenicillin | bacterial infections (not specified) | first-choice severe community-acquired pneumonia, complicated severe acute malnutrition, sepsis in neonates and children, syphilis | specification | first line for susceptible streptococcal infections and neonatal sepsis (Fuchs et al. 2018; Marum et al. 2021; Bennett et al. 2012) | efficacy and good safety, narrow-spectrum (Rote Liste 2025a; Marum et al. 2021) | high need and widely used (Bennett et al. 2012; Bos et al. 2017) | retained | increasing | advantage of efficacy and narrow spectrum |
| flucytosine | fungal infections | fungal infections | no | yes (Sigera and Denning 2023; Harsanyi et al. 2017) | effective but toxicity requires monitoring (Folk et al. 2016) | importance in severe fungal infections (Harsanyi et al. 2017; Sprute et al. 2023) | retained | increasing | synergistic benefit with amphotericin, increasing importance in HIV treatment, proven efficacy but problems with availability (Sprute et al. 2023; Harsanyi et al. 2017; Molloy et al. 2018) |
| paromomycin | amoebic infections | leishmaniac infections | change of indication | yes (Zhang et al. 2025) | effective and acceptable safety (Kim et al. 2009; Salah et al. 2013; Matos et al. 2020 | increasing importance in endemic areas (Zhang et al. 2025; Sundar and Chakravarty 2008) | retained | increasing | expanded role in leishmanisasis, useful where antimonials or amphotericin limited (Kim et al. 2009; Zhang et al. 2025 |
| rifampicin | leprosy | leprosy, tuberculosis | addition of tuberculosis | yes (Sivakumaran et al. 2024; Haigh et al. 2024) | effective and acceptable safety, use in combination therapies because of bacterial resistance risk (Sivakumaran et al. 2024; Richardus e t al. 2021; Li et al. 2022; Kim et al. 2023a; Menzies et al. 2023) | widely used globally (Haigh et al. 2024; Government of Canada 2025) | retained | increasing | widely used, public health need, effective (Haigh et al. 2024; Government of Canada 2025) |
| ***Table S1 (continued)*** | | | | | | | | | |
| ***Retained drugs*** | ***Indication EML 1977*** | ***Indication EML 2025*** | ***Change*** | ***Recommendation in guidelines*** | ***Effectiveness and safety*** | ***Public health need*** | ***Suggestion*** | ***Clinical use*** | ***Explanation*** |
| methotrexate | cancer (not specified) | cytotoxic (ALL, acute promyelocytic leukaemia, anaplastic large cell lymphoma, Burkitt lymphoma, early stage breast cancer, gestational trophoblastic neoplasia, Langerhans cell histiocytosis, osteosarcoma), dermatology (not specified), anti-rheumatic (DMARDs), juvenile joint diseases | addition for rheumatic disorders, specification | yes (Perrotta et al. 2024; Koźmiński et al. 2020) | efficacy in cancer and rheumatology, but adverse effects and toxicity require monitoring (Perrotta et al. 2024; Koźmiński et al. 2020; Taylor et al. 2021; Hamed et al. 2022) | widely used (Koźmiński et al. 2020; Friedman and Cronstein 2020) | retained | increasing | important role in cancer and rheumatic disorders |
| acetylsalicylic acid | pain, anti-inflammatory | pain, anti-inflammatory, acute migraine attack, thrombocyte aggregation inhibitor, prevention of atherosclerotic CD, juvenile joint | addition for migraine, cardiovascular system and joint | yes, first-choice for prevention of CV and thromboembolic complications, moderate pain and rheumatic disorders (Rao et al. 2025; Kumbhani et al. 2025; Ciurtin et al. 2025; Amaechi et al. 2021) | strong evidence for analgesia and secondary prevention of CV, long-term adverse effects require risk–benefit assessment for primary prevention (Garcia and Aguiar 2025; Ciurtin et al. 2025; La Torre et al. 2025; Schachtel et al. 2010; Yilmaz et al. 2022) | widely used for analgesia and CV prevention (Montinari et al. 2019; Garcia and Aguiar 2025) | retained | increasing | broader listing reflects expanded preventive uses and pediatric rheumatologic indications, long-term use associated with adverse effects (Yilmaz et al. 2022; Alves-Cabratosa et al. 2025) |
| allopurinol | pain, anti-inflammatory | tumour lysis syndrome, gout | addition for cancer treatment, specification | yes (NICE 2022b; Chan et al. 2025) | evidence for gout prophylaxis and preventing hyperuricaemia in tumour lysis syndrome contexts, acceptable safety, hypersensitivity syndrome rare but serious (Cortes et al. 2010; Yang et al. 2015; | widely used in gout and importance in oncology (Yang et al. 2015) | retained | increasing | established urate-lowering agent with added importance in prevention of complications in cancer treatment |
| naloxone | narcotic antagonist | antidote | no | antidote for MOR agonist overdose/ induced respiratory depression (WHO 2025c) | evidence for life-saving reversal of opioid overdose, rapid onset but short half-life, minimal adverse effects relative to benefit (Hennessy et al. 2024; EMCDDA 2015; Rzasa Lynn and Galinkin 2018) | important in emergency care and public health/ opioid use disorder (DEA 2025; Hennessy et al. 2024; EMCDDA 2015) | retained | increasing | life-saving antidote |
| betamethasone | antiinflammatory, topical | antiinflammatory, antipruritic, topical | specification | topical GCR agonist for inflammatory dermatoses (Wollenberg et al. 2025) | strong efficacy for inflammatory skin diseases, local atrophy and systemic absorption risks with prolonged use, steroid-sparing strategies considered for long-term use (Wollenberg et al. 2025; Gisondi et al. 2024; Pofi et al. 2023) | widely used in dermatology and rheumatology, importance in many short-term inflammatory conditions (Benzon et al. 2025) | retained | increasing | important GCR agonist for dermatological conditions, emerging importance in rheumatology |
| dexamethasone | adrenal hormone substitution | allergic reaction, hormone replacement, cancer treatment (ALL, anaplastic large cell lymphoma, Burkitt lymphoma, multiple myeloma), emesis | addition and change of indications | widely recommended for multiple indications: as part of chemotherapy regimens, for severe allergic reactions, cerebral edema, and in specified severe respiratory inflammatory conditions (MSF 2025f; NHS 2025b) | robust evidence across many indications including oncology adjuncts and life-saving in pediatric emergency, adverse effects significant but acceptable in indicated regimens (Giolito et al. 2025; Ottaiano et al. 2025; Mo et al. 2024; An et al. 2024; Weinstein et al. 2024) | high need in oncology and emergency medicine (Ottaiano et al. 2025; Weinstein et al. 2024) | retained | increasing | versatile GCR agonist with broad evidence-based use, expanding indications |
| hydrocortisone | adrenal hormone substitution | adrenal hormone substitution, anti-inflammatory (gastrointestinal, dermatology/topical), cancer treatment (ALL, Burkitt lymphoma), allergic reaction | addition of indication for cancer treatment, allergic reactions and inflammatory conditions | first-line replacement for adrenal insufficiency, cancer treatment and inflammatory conditions, but not recommended as standard in allergic shock (Pazderska and Pearce 2017; Whyte et al. 2022; Das and Panda 2017; Ornetti et al. 2025) | lifesaving adrenal replacement and effective in short-term anti-inflammatory effects, strong probability of chronic systemic adverse effects (Martinez and Sabatier 2025; Aldea et al. 2020; Das and Panda 2017; Ornetti et al. 2025; Yasir et al. 2023) | essential in inflammatory conditions, endocrine disorders and part of cancer treatment regimens (Yasir et al. 2023) | retained | increasing | important hormonal replacement with wide emergency and long-term treatment indications, but withdrawal as first line for anaphylaxis (White et al. 2022; Martinez and Sabatier 2025) |
| levothyroxine | thyroid hormone substitution | thyroid hormone substitution | no | hypothyreoidism (Centanni et al. 2025) | effective and safe when dosed and monitored appropriately, essential for brain development in children, need of individual dosage adjustment and control of TSH values (Centanni et al. 2025; Colucci et al. 2013; Esposito et al. 2022) | essential in maintenance treatment of hypothyreoidism, prevention of mental retardation in congenital hypothyroidism, high and expanding prevalence (Kahaly and Gottwald-Hostalek 2022; Seo et al. 2017) | retained | increasing | effective hypothyroidism therapy, globally increasing prevalence but common overuse (Bindel and Seifert 2025e; Calissendorff 2023) |
| prednisolone | adrenal hormone substitution | adrenal hormone substitution, anti-inflammatory (gastrointestinal, ophthalmology/topical), cancer treatment (ALL, anaplastic large cell lymphoma, Burkitt lymphoma, CLL, diffuse large B-cell lymphoma, follicular lymphoma, Hodkin lymphoma, Langerhans cell histiocytosis, metastatic castration-resistant prostate cancer, multiple myelome), allergic reaction, seizues, cluster headache | addition of cancer treatment, anti-inflammatory conditions, symptomatic CNS, allergic reactions | systemic GCR agonist for many inflammatory, oncologic supportive and neurologic symptomatic uses, but not initial therapy in anaphylactic shock (Faggiano et al. 2022; Kashyap and Chabri 2023; Walsh et al. 2025) | robust across many acute and chronic inflammatory and oncologic indications, in chronic use adverse effects expectable (Faggiano et al. 2022; Wijdicks 2024; Obermann and Holle 2020; Kashyap and Chabri 2023; Becker and Kaindl 2023; Offor et al. 2025) | widely used across many medical fields | retained | increasing | wide evidence-based indications; expanded listing reflects clinical practice breadth |
| measles vaccine | vaccination | vaccination | no | routine childhood immunization (WHO 2013) | effective and safe, prevents high morbidity/mortality, mild and transient adverse effects (e.g. fever) possible (Sattani 2025; Benn and Aaby 2023; Dattani and Spooner 2025; Government of Canada 2025b) | indispensable for global measles elimination goals (WHO 2013) | retained | increasing | vaccine with major public-health impact, resurgence of measles (Parums 2024; Dattani and Spooner 2025) |
| typhoid vaccine | vaccination | vaccination | no | prevention of typhus in endemic settings and high-risk travel (Brendan et al. 2015; WHO 2018c) | safe and effective, reduction of morbidity and mortality (Gloeck et al. 2025; Batool et al. 2024) | important in typhoid-endemic countries (Haposan et al. 2025) | retained | increasing | rising bacterial resistance reinforces need for prevention (Dyson et al. 2019) |
| furosemide | diuretics | diuretics, heart failure | addition of cardiovascular conditions | first-line NKCC inhibitor in heart failure, symptomatic treatment in chronic kidney disease (KDIGO 2024; Kitai et al. 2025) | effective in diuresis and reduction of fluid volume, symptom relief, mortality/morbidity benefit in heart failure, caution about adverse effects (e.g. hypovolemia, hypokalemia) and drug interactions (Song et al. 2025; Ho and Power 2010; Widiarti et al. 2024; Flyte et al. 2022) | widely used, globally high disease prevalence and need (Wu et al. 2024; Magdy et al. 2022) | retained | increasing | effective treatment with symptomatic relief and reduced mortality in heart failure, essential part of the treatment strategy, high and increasing disease burden due to aging societies (Wu et al. 2024; Magdy et al. 2022) |
| ***Table S1 (continued)*** | | | | | | | | | |
| ***Retained drugs*** | ***Indication EML 1977*** | ***Indication EML 2025*** | ***Change*** | ***Recommendation in guidelines*** | ***Effectiveness and safety*** | ***Public health need*** | ***Suggestion*** | ***Clinical use*** | ***Explanation*** |
| spironolactone | diuretics | diuretics, heart failure | addition of cardiovascular conditions | first-line MRA for heart failure with reduced ejection fraction, treatment-refractory hypertension (Heidenreich et al. 2022; Carey et al. 2018) | universal recommendation in heart failure, mortality reduction, but adverse effects (e.g. hypercalemia) require caution (Heidenreich et al. 2022; Chen et al. 2022; Prashar et al. 2025; Kosmas et al. 2018; Wang and Lipner 2020) | widely used, high prevalence and burden of disease (Savarese et al. 2023; Kosmas et al. 2018) | retained | increasing | expanded therapeutic role in heart failure and resistant hypertension |
| calcium gluconate | nutritional supplement | antidote, nutritional supplement | addition of indication in intoxication | treatment of hyperkalaemia and Ca-channel blocker toxicity, correction of hypocalcemia, osteoporosis treatment (NOGG 2024; Chakraborty et al. 2024; Medicines and Healthcare products Regulatory Agency 2023; St-Onge et al. 2017) | benefit in emergencies, effective in deficiencies, part of prevention regimes for osteoporosis in risk groups, mild adverse effects but risk of drug interactions (Shea et al. 2004; UKCPA 2025; Chakraborty et al. 2024; St-Onge et al. 2017) | relevance in emergency and osteoporosis treatment | retained | increasing | broad utility, safe and effective in acute settings and prevention of complications |
| epinephrine | asthma | asthma, COPD, local anaesthetic, allergic reactions, arrhythmias, mydriatic | addition for anaesthesia, allergy, cardiovascular conditions and opthalmology | first-line vasopressor in cardiogenic shock and anaphylaxis (Sinha et al. 2025; Resuscitation Council UK 2021; GINA 2025) | effective in cardiogenic shock and symptom relief in acute respiratory obstruction, rapid onset, adverse effects acceptable (Bernstein et al. 2025; Lüsebrink et al. 2024; Numa et al. 2000) | life-saving, widely used in emergency settings | retained | increasing | broad use in critial-care and emergency |
| salbutamol | asthma | asthma, COPD | addition of COPD | rescue bronchodilator for asthma and COPD (GINA 2025; GOLD 2025) | short-acting beta-agonist for acute symptom relief, effective in short-term treatment but possible cardiovascular adverse effects and tolerance development in chronic use (GOLD 2025; GINA 2025; Ma et al. 2023; Haney and Hancox 2005; Marques and Vale 2022) | high prevalence, widespread use and important treatment in acute situations, risk of overuse and wosening outcomes (Nwaru 2020) | retained | increasing | broad relevance in common chronic airway diseases |

Supplemental References

Adinarayanan, S., Critchley, J., Das, P. K., & Gelband, H. (2007). Diethylcarbamazine (DEC)-medicated salt for community-based control of lymphatic filariasis. The Cochrane database of systematic reviews, 2007(1), CD003758. https://doi.org/10.1002/14651858.CD003758.pub2

Agostoni, C., Bresson, J.L., Fairweather-Tait, S., Flynn, A., Golly, I., et al. (2011). Scientific Opinion on the substantiation of a health claim related to water and reduced risk of development of dehydration and of concomitant decrease of performance pursuant to Article 14 of Regulation (EC) No 1924/2006. EFSA, 16. February 2011. eISSN 1831-4732. https://doi.org/10.2903/j.efsa.2011.1982

Ahmad, N., Nawi, A. M., Jamhari, M. N., Nurumal, S. R., Mansor, J., Zamzuri, M. I. A., Yin, T. L., & Hassan, M. R. (2022). Post-Exposure Prophylactic Vaccination against Rabies: A Systematic Review. Iranian journal of public health, 51(5), 967–977. https://doi.org/10.18502/ijph.v51i5.9412

Ainyanbhor, I.E., Edo, G.I., Ali, A.B., et al. (2025). Ibuprofen: A review on its synthesis, mechanism of action, pharmacological properties, and environmental impact. Pharmacological Research - Reports, Volume 4, December 2025, 100066. https://doi.org/10.1016/j.prerep.2025.100066

Airainer, M., Seifert, R. (2024). Lithium, the gold standard drug for bipolar disorder: analysis of current clinical studies. Naunyn-Schmiedeberg's Arch Pharmacol 397, 9723–9743 (2024). https://doi.org/10.1007/s00210-024-03210-8

Akinosoglou K, Rigopoulos EA, Papageorgiou D, Schinas G, Polyzou E, Dimopoulou E, Gogos C, Dimopoulos G. Amphotericin B in the Era of New Antifungals: Where Will It Stand? J Fungi (Basel). 2024 Apr 10;10(4):278. doi: 10.3390/jof10040278. PMID: 38667949; PMCID: PMC11051097.

Alchin, J., Dhar, A., Siddiqui, K., & Christo, P. J. (2022). Why paracetamol (acetaminophen) is a suitable first choice for treating mild to moderate acute pain in adults with liver, kidney or cardiovascular disease, gastrointestinal disorders, asthma, or who are older. Current medical research and opinion, 38(5), 811–825. https://doi.org/10.1080/03007995.2022.2049551

Alouache, A. (2025). Vitamin A: Benefits and Consequences of Its Deficiency on Health. Vitamins and Health, May 7 2025. DOI: 10.5772/intechopen.1010232

Alrefaei, A.E., Alzahrani. M.A., Alsuhaim, S.A. (2022). Cyclophosphamide related toxicity; a systematic review. International Journal of Medicine in Developing Countries, 2022;6(5):740–747. https://doi.org/10.24911/IJMDC.51-1641243189

Alsaad, N., van Altena, R., Pranger, A.D. (2013). Evaluation of co-trimoxazole in the treatment of multidrug-resistant tuberculosis. uropean Respiratory Journal 2013 42(2): 504-512; DOI: https://doi.org/10.1183/09031936.00114812

Alster, T. (2013). Review of Lidocaine/Tetracaine Cream as a Topical Anesthetic for Dermatologic Laser Procedures. Pain and therapy, Volume 1, 2013. https://www.springermedizin.de/review-of-lidocaine-tetracaine-cream-as-a-topical-anesthetic-for/9339818

Alves-Cabratosa, L., López, C., Garcia-Gil, M., Tornabell-Noguera, È., Comas-Cufí, M., Blanch, J., Martí-Lluch, R., Ponjoan, A., Domínguez-Armengol, G., Zacarías-Pons, L., Ribas-Aulinas, F., Balló, E., & Ramos, R. (2025). Long-Term Effectiveness of Acetylsalicylic Acid in Primary Prevention of Cardiovascular Diseases and Mortality in Patients at High Risk, a Retrospective Cohort Study-The JOANA Study. Journal of clinical medicine, 14(16), 5710. https://doi.org/10.3390/jcm14165710

American Academy of Pediatric Dentistry (AAPD). (2023). Use of nitrous oxide for pediatric dental patients. The Reference Manual of Pediatric Dentistry. Chicago, IL: American Academy of Pediatric Dentistry; 2025:415-22. https://www.aapd.org/media/Policies_Guidelines/BP_UseofNitrous.pdf

American College of Radiology (ACR). ACR Manual on Contrast Media, 2025. ACR Committee on Drugs and Contrast Media. Last updated: 2025. Last accessed: December 6 2025. https://edge.sitecorecloud.io/americancoldf5f-acrorgf92a-productioncb02-3650/media/ACR/Files/Clinical/Contrast-Manual/ACR-Manual-on-Contrast-Media.pdf

American College of Surgeons (ACS). (2024). The Management of Traumatic Brain Injury. Best Practices Guidelines. ACS, Chicago. Last updated: 2024. Last accessed: December 7 2025. https://www.facs.org/media/vgfgjpfk/best-practices-guidelines-traumatic-brain-injury.pdf

Anand, U., Dey, A., Singh Chandel, A.K., Sanyal, R., et al. (2023). Cancer chemotherapy and beyond: Current status, drug candidates, associated risks and progress in targeted therapeutics. Genes & Diseases. Volume 10, Issue 4, July 2023, Pages 1367-1401. https://doi.org/10.1016/j.gendis.2022.02.007

Antza, C., Dimou, C., Doundoulakis I., et al. (2020). The flipside of hydralazine in pregnancy: A systematic review and meta-analysis. Pregnancy Hypertension, Volume 19, January 2020, Pages 177-186. https://doi.org/10.1016/j.preghy.2020.01.011

Archer, D. F., Nakajima, S. T., Sawyer, A. T., Wentworth, J., Trupin, S., Koltun, W. D., Gilbert, R. D., & Ellman, H. (2013). Norethindrone acetate 1.0 milligram and ethinyl estradiol 10 micrograms as an ultra low-dose oral contraceptive. Obstetrics and gynecology, 122(3), 601–607. https://doi.org/10.1097/AOG.0b013e3182a1741c

Aronson, J.K. (2016). Amodiaquine. Meyler's Side Effects of Drugs. The International Encyclopedia of Adverse Drug Reactions and Interactions. 16th edition, 2016, pages 302-304. https://doi.org/10.1016/B978-0-444-53717-1.00282-1

Ashley, E.A., Recht, J. & White, N.J. (2014). Primaquine: the risks and the benefits. Malar J 13, 418 (2014). https://doi.org/10.1186/1475-2875-13-418

Assefa, M., Girmay, G. (2024). Prevalence of co-trimoxazole resistance among HIV-infected individuals in Ethiopia: a systematic review and meta-analysis. Front. Med., 11 July 2024. Sec. Infectious Diseases: Pathogenesis and Therapy. Volume 11 - 2024. https://doi.org/10.3389/fmed.2024.1418954

Ates, I., Sahin, H., Irham, L. M., Yilmaz, S., & Suzen, S. (2025). The Role of Hydroxychloroquine in the Management of Rheumatic Disorders: A Comprehensive Review. Basic & clinical pharmacology & toxicology, 137(2), e70082. https://doi.org/10.1111/bcpt.70082

Audibert, C., Hugo, P., Gosling, R., Eddis, C., Center, M., & Tchouatieu, A. M. (2025). Projected uptake of sulfadoxine-pyrimethamine for perennial malaria chemoprevention in children under 2 years of age in nine sub-Saharan African countries: an epidemiologically-based 5-year forecast analysis. Malaria journal, 24(1), 124. https://doi.org/10.1186/s12936-025-05355-0

Ayoub S. S. (2021). Paracetamol (acetaminophen): A familiar drug with an unexplained mechanism of action. Temperature (Austin, Tex.), 8(4), 351–371. https://doi.org/10.1080/23328940.2021.1886392

Babalska, Z. Ł., Korbecka-Paczkowska, M., & Karpiński, T. M. (2021). Wound Antiseptics and European Guidelines for Antiseptic Application in Wound Treatment. Pharmaceuticals, 14(12), 1253. https://doi.org/10.3390/ph14121253

Baddam, S., Khan, K.N. Jialal, I. (2025). Folic Acid Deficiency. StatPearls, Treasure Island (FL). Last updated: June 25 2025. Last accessed: November 30 2025. https://www.ncbi.nlm.nih.gov/books/NBK535377/

Bai, A.D., Showler, A.S., Burry, L., Steinberg, M., Ricciuto, D.R., Fernandes, T., Chiu, A., Raybardhan, S. et al. (2015). Comparative effectiveness of cefazolin versus cloxacillin as definitive antibiotic therapy for MSSA bacteraemia: results from a large multicentre cohort study, Journal of Antimicrobial Chemotherapy, Volume 70, Issue 5, May 2015, Pages 1539–1546, https://doi.org/10.1093/jac/dku560.

Bakel, L. A., Hamid, J., Ewusie, J., Liu, K., Mussa, J., Straus, S., Parkin, P., & Cohen, E. (2017). International Variation in Asthma and Bronchiolitis Guidelines. Pediatrics, 140(5), e20170092. https://doi.org/10.1542/peds.2017-0092

Baker, E., Roberts, A.P., Wilde, K., et al. (2010). Development of a core drug list towards improving prescribing education and reducing errors in the UK. BJCP, Volume 71, Issue 2, February 2011, Pages 190-198. https://doi.org/10.1111/j.1365-2125.2010.03823.x

Balakumar, P., Rohilla, A., Thangathiruapthi, A. (2010). Gentamicin-induced nephrotoxicity: Do we have a promising therapeutic approach to blunt it? Pharmacological Research, Volume 62, Issue 3, September 2010, Pages 179-186. https://doi.org/10.1016/j.phrs.2010.04.004

Bansal, D., & Purohit, V. K. (2013). Accessibility and use of essential medicines in health care: Current progress and challenges in India. Journal of pharmacology & pharmacotherapeutics, 4(1), 13–18. https://doi.org/10.4103/0976-500X.107642

Barnett, S., Hellmann, F., Parke, E., Makin, G., Tweddle, D. A., Osborne, C., Hempel, G., & Veal, G. J. (2022). Vincristine dosing, drug exposure and therapeutic drug monitoring in neonate and infant cancer patients. European journal of cancer (Oxford, England : 1990), 164, 127–136. https://doi.org/10.1016/j.ejca.2021.09.014

BC Cancer. (2023). Fluorouracil. BC Cancer Drug Manual©. BC Cancer Provincial Pharmacy, Provincial Health Services Authority. Last updated: June 1 2023. Last accessed: November 30 2025. https://www.bccancer.bc.ca/drug-database-site/Drug%20Index/Fluorouracil_monograph.pdf

Bean, D.C., Wareham, D.W. (2017). Pentamidine: a drug to consider re-purposing in the targeted treatment of multi-drug resistant bacterial infections? JLPM, Vol 2, July 2017. doi: 10.21037/jlpm.2017.06.18

Bekele, T., Lachisa, L., Tsegaye, A. et al. (2024). Efficacy of Albendazole and Mebendazole Against Soil Transmitted Infections among Pre-School and School Age Children: A Systematic Review and Meta-Analysis. J Epidemiol Glob Health 14, 884–904. https://doi.org/10.1007/s44197-024-00231-7

Bekka, E., Liakoni, E. (2024). Anticoagulation reversal (vitamin K, prothrombin complex concentrates, idarucizumab, andexanet-α, protamine). BJCP, Volume 91, Issue 3, Themed Issue: Update on Antidotes and Other Treatments in Managing Acutely Poisoned Patients, March 2025, Pages 604-614. https://doi.org/10.1111/bcp.16142

Bell DJ, Nyirongo SK, Mukaka M, Zijlstra EE, Plowe CV, et al. (2008) Sulfadoxine-Pyrimethamine–Based Combinations for Malaria: A Randomised Blinded Trial to Compare Efficacy, Safety and Selection of Resistance in Malawi. PLOS ONE 3(2): e1578. https://doi.org/10.1371/journal.pone.0001578

Berger, M.M., Shenkin, A., Dizdar, O.S., et al. (2024). ESPEN practical short micronutrient guideline. Clnical Nutrition 43, 2024, p825-857. https://doi.org/10.1016/j.clnu.2024.01.030

Best, E. J., Gazarian, M., Cohn, R., Wilkinson, M., & Palasanthiran, P. (2011). Once-daily gentamicin in infants and children: a prospective cohort study evaluating safety and the role of therapeutic drug monitoring in minimizing toxicity. The Pediatric infectious disease journal, 30(10), 827–832. https://doi.org/10.1097/INF.0b013e31821e405d

Beydoun, A., DuPont, S., Zhou, D., et al. (2020). Current role of carbamazepine and oxcarbazepine in the management of epilepsy. Seizure, Volume 83, December 2020, Pages 251-263. https://doi.org/10.1016/j.seizure.2020.10.018

Biersack, B. (2024). The Antifungal Potential of Niclosamide and Structurally Related Salicylanilides. International Journal of Molecular Sciences, 25(11), 5977. https://doi.org/10.3390/ijms25115977

Bindel, L. J., & Seifert, R. (2025e). Long-term forecasting and evaluation of medicine consumption for the ATC class H with a focus on thyroid hormones in OECD countries using ARIMA models. Naunyn-Schmiedeberg's archives of pharmacology, 398(8), 10691–10717. https://doi.org/10.1007/s00210-025-03930-5

Bindel, L.J., Seifert, R. (2025a). Determinants of prescribing behaviour of antibacterial drugs in Europe and use of appropriate nomenclature in the literature. Naunyn-Schmiedeberg's Arch Pharmacol. https://doi.org/10.1007/s00210-025-04511-2

Bindel, L.J., Seifert, R. (2025b). Evidence of lithium underuse in bipolar disorder: analysis of lithium and antipsychotic consumption, prediction of future trends, regional disparities and indicators of rational and inappropriate use in Europe. Naunyn-Schmiedeberg's Arch Pharmacol. https://doi.org/10.1007/s00210-025-04389-0

Bindel, L.J., Seifert, R. (2025c). Development of bacterial resistance in Germany from 2008 to 2022 — major culprit pathogens, antibacterial drugs, and prescribing practices. Naunyn-Schmiedeberg's Arch Pharmacol 398, 4219–4236. https://doi.org/10.1007/s00210-024-03533-6

Bindel, L.J., Seifert, R. (2025d). Trends, prescribing patterns and projections of antiseizure drug use in Europe. Epilepsy Research, Volume 218, December 2025, 107689. https://doi.org/10.1016/j.eplepsyres.2025.107689

Bindel, L.J., Seifert, R. (2025f) AWaRe classification analysis for European countries with ARIMA forecasts to assess prescribing patterns and ‘One Health’ targets. Naunyn-Schmiedeberg's Arch Pharmacol 398, 13707–13729 (2025). https://doi.org/10.1007/s00210-025-04121-y

"Boniphace, I., Honarati, M., Wilbert, B., et al. (2007). Randomized controlled safety and efficacy trial of 2 vitamin A supplementation schedules in Tanzanian infants.

The American Journal of Clinical Nutrition, Volume 85, Issue 5, May 2007, Pages 1312-1319. https://doi.org/10.1093/ajcn/85.5.1312"

Bornstein, S.R., Allolio, B., Arlst, W., et al. (2016). Diagnosis and Treatment of Primary Adrenal Insufficiency: An Endocrine Society Clinical Practice Guideline, The Journal of Clinical Endocrinology & Metabolism, Volume 101, Issue 2, 1 February 2016, Pages 364–389, https://doi.org/10.1210/jc.2015-1710

Boswell, J., Lowe, H., Adorati, K., et al. (2019). Chapter 3 - Lithium. Side Effects of Drugs Annual, Volume 41, 2019, Pages 27-31. https://doi.org/10.1016/bs.seda.2019.07.011

Brigo, F., Igwe, S. C., & Lattanzi, S. (2021). Ethosuximide, sodium valproate or lamotrigine for absence seizures in children and adolescents. The Cochrane database of systematic reviews, 1(1), CD003032. https://doi.org/10.1002/14651858.CD003032.pub5

Bru, J.P., Garraffo, R. (2012). Role of intravenous cloxacillin for inpatient infections. Médecine et Maladies Infectieuses. Volume 42, Issue 6, June 2012, Pages 241-246. https://doi.org/10.1016/j.medmal.2011.10.015

Bruins, M., & Kraemer, K. (2013). Public health programmes for vitamin A deficiency control. Community eye health, 26(84), 69–70.

Brummaier, T., Pohanka, E., Studnicka-Benkee, A., et al. (2013). Using cyclophosphamide in inflammatory rheumatic diseases. European Journal of Internal Medicine, Volume 24, Issue 7, October 2013, Pages 590-596. https://doi.org/10.1016/j.ejim.2013.02.008

Burdet, C., Saidani, N., Dupieux, C., Lemaignen, A., et al. (2025). Cloxacillin versus cefazolin for meticillin-susceptible Staphylococcus aureus bacteraemia (CloCeBa): a prospective, open-label, multicentre, non-inferiority, randomised clinical trial. The Lancet, Volume 406, Issue 10517, 2349 - 2359. https://doi.org/10.1016/S0140-6736(25)01624-1

Bwakura-Dangarembizi, M., Kendall, L., Bakeera-Kitaka, S., et al. (2014). A Randomized Trial of Prolonged Co-trimoxazole in HIV-Infected Children in Africa. N Engl J Med 2014;370:41-53. DOI: 10.1056/NEJMoa1214901

Calissendorff, J. (2023). The dangerous trend of levothyroxine medicalization. Trends in Endocrinology & Metabolism, Volume 34, Issue 12, December 2023, Pages 783-785. https://doi.org/10.1016/j.tem.2023.08.017

Canadian Agency for Drugs and Technologies in Health. (2016). Oral Iron for Anemia: A Review of the Clinical Effectiveness, Cost-effectiveness and Guidelines. Canadian Agency for Drugs and Technologies in Health, Ottawa (ON). Last updated: January 6 2016. Last accessed: NOvember 30 2025. https://www.ncbi.nlm.nih.gov/books/NBK343970/

Cavassin FB, Baú-Carneiro JL, Vilas-Boas RR, Queiroz-Telles F. Sixty years of Amphotericin B: An Overview of the Main Antifungal Agent Used to Treat Invasive Fungal Infections. Infect Dis Ther. 2021 Mar;10(1):115-147. doi: 10.1007/s40121-020-00382-7. Epub 2021 Feb 1. PMID: 33523419; PMCID: PMC7954977.

Ćelić, I., Bach-Rojecky, L., Soldo, A., et al. (2020). Resolving Issues About Efficacy and Safety of LowDose Codeine in Combination Analgesic Drugs: A Systematic Review. Pain Ther 9:171–194. https://doi.org/10.1007/s40122-020-00162-8

Cesur, S., Bahar, K., Erekul, S. (2002). Death from cumulative sodium stibogluconate toxicity on Kala-Azar. CMI, Volume 8, Issue 9, p606, September 2002. https://doi.org/10.1046/j.1469-0691.2002.00456.x

Chak, G., Patel, R., Allingham, R., etal. (2015). Acetazolamide: Considerations for Systemic Administration. Ophthalmic Pearls, MAR 01, 2015. Last accessed: December 7 2025. https://www.aao.org/Assets/480e1f43-6366-4f12-8e13-b5fc36d95e08/635601432556000000/march-2015-ophthalmic-pearls-pdf

Chan, P. A., Le Brazidec, D. L., Becasen, J. S., Martin, H., Kapadia, J., Reno, H., Bachmann, L., & Barbee, L. A. (2023). Safety of Longer-Term Doxycycline Use: A Systematic Review and Meta-Analysis With Implications for Bacterial Sexually Transmitted Infection Chemoprophylaxis. Sexually transmitted diseases, 50(11), 701–712. https://doi.org/10.1097/OLQ.0000000000001865

Chan, Y.L., Sharkawi, D.E., Chanouzas, D., O'Connor, D., et al. (2025). British Society for Haematology updated guidelines for the diagnosis and management of tumour lysis syndrome in adults and children with haematological malignancies: A focus on patient safety. BJHaem, Volume 207, Issue 4, October 2025, Pages 1248-1258. https://doi.org/10.1111/bjh.70092

Chaudhary K. (2011). Peritoneal Dialysis Drop-out: Causes and Prevention Strategies. International journal of nephrology, 2011, 434608. https://doi.org/10.4061/2011/434608

Chen, B., Ni, Y., Chen, J., Xing, S., Zang, Z. (2024). Comparison of different concentrations atropine in controlling children and adolescent myopia: an umbrella review of systematic reviews and meta-analyses. Front. Ophthalmol., Sec. Pediatric Ophthalmology and Strabismus. Volume 4, 23 October 2024. https://doi.org/10.3389/fopht.2024.1447558

Chen, S., Khusial, T., Patel, D., Sing, S., Yakubova, T., Wang, A., Nguyen, T. (2015). Digoxin Use in Modern Medicine. US Pharm. 2015;40(2):44-48. https://www.uspharmacist.com/article/digoxin-use-in-modern-medicine#:~:text=3-,Conclusion,rather%20than%20first%2Dline%20therapy.

Chen, W., Mook, R. A., Jr, Premont, R. T., & Wang, J. (2018). Niclosamide: Beyond an antihelminthic drug. Cellular signalling, 41, 89–96. https://doi.org/10.1016/j.cellsig.2017.04.001

Chia, Y.C., Turana, Y., Sukonthasarn, A., et al. (2021). Comparison of guidelines for the management of hypertension: Similarities and differences between international and Asian countries; perspectives from HOPE-Asia Network. JCH, Colume 23, Issue 3, Pages 422-434. https://doi.org/10.1111/jch.14226

Cholo, M. C., Steel, H. C., Fourie, P. B., Germishuizen, W. A., & Anderson, R. (2012). Clofazimine: current status and future prospects. The Journal of antimicrobial chemotherapy, 67(2), 290–298. https://doi.org/10.1093/jac/dkr444

Chu, J.H., Antachopoulos, C., Walsh, T.J. (2008). CHAPTER 5 - Antifungal Agents. Pediatric Infectious Diseases. The Requisites in Pediatrics, 2008, Pages 37-47. https://doi.org/10.1016/B978-0-323-02041-1.50011-0

Cole, R.T., Kalogeropoulos, A.P., Gheorghiade, M., Quyyumi, A., et al. (2011). Hydralazine and Isosorbide Dinitrate in Heart Failure: Historical Perspective, Mechanisms, and Future Directions. Circulation, Vol. 123, Number 21. https://doi.org/10.1161/CIRCULATIONAHA.110.012781

Conner-Kerr T. (2014). The Topical Evolution: Free Ions, Orthomolecular Agents, Phytochemicals, and Insect-Produced Substances. Advances in wound care, 3(8), 530–536. https://doi.org/10.1089/wound.2014.0563

Connoly, S.J., Ezekowitz, M.D., Yusuf, S., et al. (2009). Dabigatran versus Warfarin in Patients with Atrial Fibrillation. NEJM 2009;361:1139-1151. DOI: 10.1056/NEJMoa0905561

Corsetti, M., Lacerda, A., Patel, K., Aran, A. (2024). Are all stimulant laxatives the same? A comparative perspective on bisacodyl, sodium picosulfate (SPS) and senna. EFSM 2024;4:240040. DOI: 10.52778/efsm.24.0040

Cortes, J., Aluh, D., Fronteira, I., Gil, D., Aguiar, P. (2025). Sixth wave of global public health progress. One Health. Volume 20, June 2025, 101015. https://doi.org/10.1016/j.onehlt.2025.101015

Countouris, M., Mahmoud, Z., Cohen, J.B., et al. (2025). Hypertension in Pregnancy and Postpartum: Current Standards and Opportunities to Improve Care. Circulation, Volume 151, Number 7. https://doi.org/10.1161/CIRCULATIONAHA.124.073302

Crespillo-Andújar, C., Chamorro-Tojeiro, S., Norman, F., Monge-Maillo, B., López-Vélez, R., & Pérez-Molina, J. A. (2018). Toxicity of nifurtimox as second-line treatment after benznidazole intolerance in patients with chronic Chagas disease: when available options fail. Clinical microbiology and infection : the official publication of the European Society of Clinical Microbiology and Infectious Diseases, 24(12), 1344.e1–1344.e4. https://doi.org/10.1016/j.cmi.2018.06.006

Cronberg, O., Tyrstrup, M., Beckman, A., Carlsson, et al. (2025). Penicillin V as first-line treatment of pneumonia in primary care: a registry-based study. CMI, 23 August 2025. https://doi.org/10.1016/j.cmi.2025.08.016

Currier, G. W., & Trenton, A. (2002). Pharmacological treatment of psychotic agitation. CNS drugs, 16(4), 219–228. https://doi.org/10.2165/00023210-200216040-00002

"da Silva Lopes, J.P., Barbosa, J., Dinis-Oliveira, R.J. (2024). Clinical and forensic aspects of potassium iodide: Suddenly in high demand across Europe due to fears of radiation poisoning from a nuclear attack in Ukraine. BCPT, Volume 135, Issue 3, September 2024,

Pages 250-270. https://doi.org/10.1111/bcpt.14052"

Daniels, B., Kuhn, L., Spooner, E., Mulol, H., Goga, A., Feucht, U., Essack, S. Y., & Coutsoudis, A. (2022). Cotrimoxazole guidelines for infants who are HIV-exposed but uninfected: a call for a public health and ethics approach to the evidence. The Lancet. Global health, 10(8), e1198–e1203. https://doi.org/10.1016/S2214-109X(22)00120-6

Dawwas, G. K., Lewis, J. D., & Cuker, A. (2025). Direct Oral Anticoagulants Compared With Warfarin in Patients With Atrial Fibrillation and Valvular Heart Disease Without Mechanical Valves. Journal of the American Heart Association, 14(4), e035478. https://doi.org/10.1161/JAHA.124.035478

De Backer, J., Haugaa, K.H., Hasselberg, N.E., de Hosson, M., et al. (2025). 2025 ESC Guidelines for the management of cardiovascular disease and pregnancy: Developed by the task force on the management of cardiovascular disease and pregnancy of the European Society of Cardiology (ESC), Endorsed by the European Society of Gynecology (ESG). European Heart Journal, Volume 46, Issue 43, 14 November 2025, Pages 4462–4568, https://doi.org/10.1093/eurheartj/ehaf193

De Caterina, A. R., & Leone, A. M. (2010). Why beta-blockers should not be used as first choice in uncomplicated hypertension. The American journal of cardiology, 105(10), 1433–1438. https://doi.org/10.1016/j.amjcard.2009.12.068

de Macedo, V., Meneghete, B. P., Koaski, J. C., Albuquerque, A. S., & Fachi, M. M. (2023). Doxycycline for Multidrug-Resistant Gram-Negative Bacterial Infection Treatment: A Scoping Review. Journal of global infectious diseases, 15(3), 95–100. https://doi.org/10.4103/jgid.jgid_34_23

De Morais, J., Van Aerts, L., Evans-Brown, M., et al. (2024). Recreational Use of Nitrous Oxide – a Growing Concern in Europe. Emerging Trends in Drugs, Addictions, and Health, Volume 4, December 2024, 100085. https://doi.org/10.1016/j.etdah.2023.100085

de Vries Schultink, A.H., Sallevelt, BT., Meinders, A.J., van de Garde, E.M., Roescher, N. (2021). The need for gentamicin adjunctive to cefuroxime as empirical sepsis therapy: a local protocol evaluation. CMI, Volume 27, Issue 9, p1354-1355, September 2021. https://doi.org/10.1016/j.cmi.2021.03.032

de'Angelis, G. L., Vincenzi, F., Fornaroli, F., Buonvicino, D., & Chiarugi, A. (2025). New perspectives for optimizing fever and pain management in pediatrics: evidence supporting therapeutic awareness in clinical practice. Italian journal of pediatrics, 51(1), 255. https://doi.org/10.1186/s13052-025-02107-3

Decke, J., & Seifert, R. (2025). Market analysis of vitamin C-containing dietary supplements in Germany and the USA: Consumer information and risks and benefits. Naunyn-Schmiedeberg's archives of pharmacology, 398(11), 15807–15823. https://doi.org/10.1007/s00210-025-04248-y

Demay, M. B., Pittas, A. G., Bikle, D. D., Diab, D. L., Kiely, M. E., Lazaretti-Castro, M., Lips, P., Mitchell, D. M., Murad, M. H., Powers, S., Rao, S. D., Scragg, R., Tayek, J. A., Valent, A. M., Walsh, J. M. E., & McCartney, C. R. (2024). Vitamin D for the Prevention of Disease: An Endocrine Society Clinical Practice Guideline. The Journal of clinical endocrinology and metabolism, 109(8), 1907–1947. https://doi.org/10.1210/clinem/dgae290

DeMott, J.M., Slocum, G.W., Gottlieb, M., Peksa, G.D. (2020). Levetiracetam vs. phenytoin as 2nd-line treatment for status epilepticus: A systematic review and meta-analysis. Epilepsy & Behavior, Volume 111, October 2020, 107286. https://doi.org/10.1016/j.yebeh.2020.107286

Derry, S., & Moore, R. A. (2013). Paracetamol (acetaminophen) with or without an antiemetic for acute migraine headaches in adults. The Cochrane database of systematic reviews, 2013(4), CD008040. https://doi.org/10.1002/14651858.CD008040.pub3

Deutsche Dermatologische Gesellschaft (DDG). (2025). S3-Leitlinie „Therapie der Psoriasis vulgaris“ (AWMF-Registernummer 013 - 001). Version 8.0. Last updated: July 2025. Last accessed: December 6 2025. https://register.awmf.org/de/leitlinien/detail/013-001

Deutsche Gesellschaft für Innere Medizin (DGIM). (2025). Liste unverzichtbarer Wirkstoffe in der Inneren Medizin. DGIM-Wirkstoffliste. Last updated: 2025. Last accessed: December 8 2025. https://www.dgim.de/publikationen/dgim-wirkstoffliste

Deutsche Gesellschaft für Pneumologie und Beatmungsmedizin e.V. (DGP). (2025). S2k-Leitlinie. Fachärztliche Diagnostik und Therapie von erwachsenen Patienten mit Husten. AWMF-Registernummer: 020-003, Langversion 4.1. Last updated: January 2025. Last accessed: December 7 2025. https://register.awmf.org/assets/guidelines/020-003l_S2k_Fachaerztliche-Diagnostik-Therapie-erwachsene-Patienten-Husten__2025-02_1.pdf

Deutsches Ärzteblatt. (2017). Erratum. In the clinical practice guideline entitled “Red Eye: A Guide for Non-specialists“ published in issue 17/2017 of Deutsches Ärzteblatt International. Dtsch Arztebl Int 2017; 114: 418. https://di.aerzteblatt.de/pdf.asp?id=192288

Deutsches Ärzteblatt. (2024). Nationale COVID-19-Leitlinien unterscheiden sich von WHO-Empfehlungen. Deutsches Ärzteblatt, Berlin. Last updated: April 24 2024. Last accessed: December 14 2025. https://www.aerzteblatt.de/news/nationale-covid-19-leitlinien-unterscheiden-sich-von-who-empfehlungen-b633cf0e-ff0a-4b9e-83b5-6754b6435733

Deuvekot, J.J., Ayres-de-Campos, D., Wendel, S.B., et al. (2025). European guidelines on management of arrested or protracted labor in nulliparous women. European Journal of Obstetrics & Gynecology and Reproductive Biology, Volume 311, July 2025, 114064. https://doi.org/10.1016/j.ejogrb.2025.114064

Dev, A., Narang, T., Thakur, V., Dogra, S. (2025). The Dapsone Conundrum: Understanding Implications of Adverse Reactions to Dapsone Including Dapsone Hypersensitivity Syndrome in Leprosy Patients Treated With Multidrug Therapy. Inernational Journal of Dermatology, Vlume 64, Issue 10, October 2025, Pages 1862-1869. https://doi.org/10.1111/ijd.17906

Dind, A., Vickers, D., Lowe, S. (2023). Anterior STEMI Following Ergometrine Administration for Post-Partum Haemorrhage. HLC, Volume 32, Supplement 3, S267-S268, July 2023. https://doi.org/10.1016/j.hlc.2023.06.340

Divljakovic, J., Milic, M., Timic, T., Savic, M.M. (2012). Tolerance liability of diazepam is dependent on the dose used for protracted treatment. Pharmacological Reports, Volume 64, Issue 5, September–October 2012, Pages 1116-1125. https://doi.org/10.1016/S1734-1140(12)70908-8

Dold M, Samara MT, Li C, Tardy M, Leucht S. (2015). Haloperidol versus first-generation antipsychotics for the treatment of schizophrenia and other psychotic disorders. Cochrane Database of Systematic Reviews 2015, Issue 1. Art. No.: CD009831. DOI: 10.1002/14651858.CD009831.pub2.

Donker, E.M., Timpone, P.S., Brinkman, D.J., et al. (2023). The European List of Key Medicines for Medical Education: A Modified Delphi Study. Clinical Pharmacology & Therapeutics, Volume 115, Issue 3, March 2024, Pages 515-524. https://doi.org/10.1002/cpt.3132

Drew, R.H., Sterling, T.R. (2025). Antituberculous drugs: An overview. UpToDate Inc, Wolters Kluwer, 2025. Last updated: July 01 2025. Last accessed: November 29 2025. https://www.uptodate.com/contents/antituberculous-drugs-an-overview#:~:text=First%2Dline%20agents%20for%20treatment,%2C%20and%20pyrazinamide%20%5B7%5D.

Dyson, Z. A., Klemm, E. J., Palmer, S., & Dougan, G. (2019). Antibiotic Resistance and Typhoid. Clinical infectious diseases : an official publication of the Infectious Diseases Society of America, 68(Suppl 2), S165–S170. https://doi.org/10.1093/cid/ciy1111

Easterling, T., Mundle, S., Bracken, H., et al. (2019). Oral antihypertensive regimens (nifedipine retard, labetalol, and methyldopa) for management of severe hypertension in pregnancy: an open-label, randomised controlled trial. The Lancet, Volume 394, Issue 10203, p1011-1021, September 21, 2019. https://doi.org/10.1016/S0140-6736(19)31282-6

Eisenberg, N., Panunzi, I., Wolz, A., Burzio, C., Cilliers, A., Islam, M. A., Noor, W. M., Jalon, O., Jannat-Khah, D., & Gil Cuesta, J. (2021). Diphtheria Antitoxin Administration, Outcomes, and Safety: Response to a Diphtheria Outbreak in Cox's Bazar, Bangladesh. Clinical infectious diseases : an official publication of the Infectious Diseases Society of America, 73(7), e1713–e1718. https://doi.org/10.1093/cid/ciaa1718

Electronic medicines compendium (EMC). (2023). Benzylpenicillin benzathine 2.4 Million I.U. powder for suspension for injection. EMC, UK. Last updated: July 02 2023. Last accessed: November 27 2025. https://www.medicines.org.uk/emc/product/11044/smpc#gref

Eli, C., Aiden, G., Alex, C., Victor, M. (2024). Ringer's Lactate Solution: A Comprehensive Review of Its Clinical Applications and Benefits. Toron Journal Club, April 2024. DOI:10.13140/RG.2.2.16059.02085. https://www.researchgate.net/publication/382864636_Ringer's_Lactate_Solution_A_Comprehensive_Review_of_Its_Clinical_Applications_and_Benefits

European Association of Urology (EAU). (2025). Urological Infections. Guideline. Last updated: March 2025. Last accessed: November 30 2025. https://uroweb.org/guidelines/urological-infections/chapter/the-guideline

Faculty of Sexual and Reproductive Healthcare (FSRH). FRSH Guideline. Combined Hormonal Contraception. Faculty of Sexual & Reproductive Healthcare, UK. BMJ Sexual & Reproductive Health 2019; 45:Suppl 1. http://dx.doi.org/10.1136/bmjsrh-2018-CHC

Fagan, H. A., & Baldwin, D. S. (2023). Pharmacological Treatment of Generalised Anxiety Disorder: Current Practice and Future Directions. Expert Review of Neurotherapeutics, 23(6), 535–548. https://doi.org/10.1080/14737175.2023.2211767

Falk, N., Berenstein, A.J., Moscatelli, G., et al. (2022). Effectiveness of Nifurtimox in the Treatment of Chagas Disease: a Long-Term Retrospective Cohort Study in Children and Adults. Antimicrobial Agents and Chemotherapy, Colume 66, Number 5. https://doi.org/10.1128/aac.02021-21

Fawcett W. J. (2019). Suxamethonium or rocuronium for rapid sequence induction of anaesthesia?. BJA education, 19(12), 380–382. https://doi.org/10.1016/j.bjae.2019.09.001

Federal Institute for Drugs and Medical Devices (BfArM). (2015). Codeine: Review of the risk-benefit ratio of codeine-containing drugs for the treatment of cough in children and adolescents. Pharmacovigilance - Risk information. Last updated: October 16 2015. Last accessed: December 7 2025. https://www.bfarm.de/SharedDocs/Risikoinformationen/Pharmakovigilanz/EN/RV_STP/a-f/codein.html

Federal Institute for Drugs and Medical Devices (BfArM). (2055). Liste der versorgungsrelevanten Wirkstoffe nach § 52b Abs. 3c AMG. Last updated: May 28 2025. Last accessed: December 8 2025. https://www.bfarm.de/SharedDocs/Downloads/DE/Arzneimittel/Zulassung/amInformationen/Lieferengpaesse/ListeVersorgungsrelevanteWirkstoffe.pdf?__blob=publicationFile

Fenner, A. (2019). No gentamicin for first-line gonorrhoea therapy. Nat Rev Urol 16, 448. https://doi.org/10.1038/s41585-019-0203-5

Fettiplace M, Weinberg G, Nixon H, et al. (2025). The impact of local anesthetic systemic toxicity advisories on reporting to the National Poison Data System (NPDS). Regional Anesthesia & Pain Medicine Published Online First: 21 July 2025. doi: 10.1136/rapm-2025-106464

Flemming, K., (2010). The Use of Morphine to Treat Cancer-Related Pain: A Synthesis of Quantitative and Qualitative Research. Journal of Pain and Symptom Management, Volume 39, Issue 1, January 2010, Pages 139-154. https://doi.org/10.1016/j.jpainsymman.2009.05.014

Flythe, J.E., Assimon, M.M., Tugman, M.J., et al. (2022). Efficacy, Safety, and Tolerability of Oral Furosemide Among Patients Receiving Hemodialysis: A Pilot Study. KI Reports, Volume 7, Issue 10, p2186-2195, October 2022. https://doi.org/10.1016/j.ekir.2022.07.003

Food and Drug Administration (FDA). (2017). ESTROSTEP Fe (Norethindrone Acetate and Ethinyl Estradiol Tablets, USP and Ferrous Fumarate Tablets). FDA, United States. Reference ID: 4136749. Last updated: August 2017. Last accessed: December 6 2025. https://www.accessdata.fda.gov/drugsatfda_docs/label/2017/020130s017lbl.pdf

Food and Drug Adminstration (FDA). (2020). FDA cautions against use of hydroxychloroquine or chloroquine for COVID-19 outside of the hospital setting or a clinical trial due to risk of heart rhythm problems. Drug Safety and Availability. Last updated: July 1 2020. Last accessed: November 28 2025. https://www.fda.gov/drugs/drug-safety-and-availability/fda-cautions-against-use-hydroxychloroquine-or-chloroquine-covid-19-outside-hospital-setting-or

Food and Drug Adminstration (FDA). (2022b).Executive Order 13944 List of Essential Medicines, Medical Countermeasures, and Critical Inputs. Last updated: May 23 2022. Last accessed: December 8 2025.https://www.fda.gov/media/143406/download?attachment

Food and Drug Adminstration (FDAa). (2022). Potassium chloride injection. FDA. Reference ID: 4998231. Last updated: 2022. Last accessed: November 28 2025.https://www.accessdata.fda.gov/drugsatfda_docs/label/2022/019904s021lbl.pdf

Fotedar, N., Ravish, H.S. (2025). A prospective study on safety and clinical efficacy of rabies biologicals in paediatric patients with category III animal exposure. Clin Exp Vaccine Res. 2025 Jan;14(1):59-66. https://doi.org/10.7774/cevr.2025.14.e1

Freo, U. (2022). Paracetamol for Multimodal Analgesia. Pain Management, 12(6), 737–750. https://doi.org/10.2217/pmt-2021-0116

Freo, U., Ruocco, C., Valerio, A., Scagnol, I., & Nisoli, E. (2021). Paracetamol: A Review of Guideline Recommendations. Journal of clinical medicine, 10(15), 3420. https://doi.org/10.3390/jcm10153420

Fujikawa, M., & Okamura, K. (2024). Graves’ hyperthyroidism treated with potassium iodide: early response and after 2 years of follow-up. European Thyroid Journal, 13(6), e240085. https://doi.org/10.1530/ETJ-24-0085

Galante, S., Castillo, R., Price, T., Tran, M., & Stramel-Stafford, S. (2025). Assessing Clinical Outcomes of Metronidazole for Intra-Abdominal Infections When Dosed Every 12 h Versus Every 8 h in a Multi-Center Health System. Antibiotics (Basel, Switzerland), 14(7), 688. https://doi.org/10.3390/antibiotics14070688

Gallop K. (2010). Review article: phenytoin use and efficacy in the ED. Emergency medicine Australasia : EMA, 22(2), 108–118. https://doi.org/10.1111/j.1742-6723.2010.01269.x

Gallos ID, Yunas I, Devall AJ, Podesek M, Tobias A, Price MJ, Oladapo OT, Coomarasamy A. (2025). Uterotonic agents for preventing postpartum haemorrhage: a network meta‐analysis. Cochrane Database of Systematic Reviews 2025, Issue 4. Art. No.: CD011689. DOI: 10.1002/14651858.CD011689.pub4.

Gandhi, M., Elfeky, O., Ertugrul, H., Chela, H. K., & Daglilar, E. (2023). Scurvy: Rediscovering a Forgotten Disease. Diseases, 11(2), 78. https://doi.org/10.3390/diseases11020078

Garg, N., Farhat, J., Dhankhar, S., Chauhan, S., Bala, R., Madaan, R., Sharma, H., Saini, M., Singh, T. G., Aldahish, A. A., Almarhoon, Z. M., Al-Omari, B., & Sharifi-Rad, J. (2025). Vincristine in Cancer Therapy: Mechanisms, Efficacy, and Future Perspectives. Current medicinal chemistry, 32(32), 6941–6959. https://doi.org/10.2174/0109298673319496240911060138

Gegia, M., Winters, N., Benedetti, A., von Soolingen, D., Menzies, D. (2017). Treatment of isoniazid-resistant tuberculosis with first-line drugs: a systematic review and meta-analysis. The Lancet Infectious Diseases, Volume 17, Issue 2, p223-234, February 2017. https://doi.org/10.1016/S1473-3099(16)30407-8

German Central Committee against Tuberculosis (DZK). (2025). Tuberculosis vaccination. Last updated: 2025. Last accessed: December 6 2025. https://www.dzk-tuberkulose.de/en/impfung

Ghafouri-Fard S, Abak A, Tondro Anamag F, Shoorei H, Fattahi F, Javadinia SA, Basiri A and Taheri M (2021) 5-Fluorouracil: A Narrative Review on the Role of Regulatory Mechanisms in Driving Resistance to This Chemotherapeutic Agent. Front. Oncol. 11:658636. doi: 10.3389/fonc.2021.658636

Giles, J., Roberts, A. (2022). Chapter Seven - Clostridioides difficile: Current overview and future perspectives. Advances in Protein Chemistry and Structural Biology. Volume 129, 2022, Pages 215-245. https://doi.org/10.1016/bs.apcsb.2021.11.003

Giordano, D., Raso, M. G., Pernice, C., Agnoletti, V., & Barbieri, V. (2015). Topical local anesthesia: focus on lidocaine–tetracaine combination. Local and Regional Anesthesia, 8, 95–100. https://doi.org/10.2147/LRA.S41836

Girard, T.D., Exline, M.C., Carson, S.S., et al. (2018). Haloperidol and Ziprasidone for Treatment of Delirium in Critical Illness. N Engl J Med 2018;379:2506-2516. DOI: 10.1056/NEJMoa1808217

Giustina, A., Bilezikian, J.P., Adler, R.A., et al. (2024). Consensus Statement on Vitamin D Status Assessment and Supplementation: Whys, Whens, and Hows. Endocrine Reviews, Volume 45, Issue 5, October 2024, Pages 625–654. https://doi.org/10.1210/endrev/bnae009

"Glauser, T.A., Cnaan, A., Shinnar, S., Hirtz, D.G., Dlugos, D., Masur, D., Clark, P.O., et al. (2010). Ethosuximide, Valproic Acid, and Lamotrigine in Childhood Absence Epilepsy. N Engl J Med 2010;362:790-799. DOI: 10.1056/NEJMoa0902014

"

Global Burden of Disease Collaborative Network. (2023). Global Burden of Disease Study 2021 (GBD 2021) Anemia Prevalence and Years Lived with Disability by Cause 1990-2021. Seattle, United States of America: Institute for Health Metrics and Evaluation (IHME), 2023. https://doi.org/10.6069/0xbc-s191

Golder, J. E., Bauer, J. D., Barker, L. A., Lemoh, C. N., Gibson, S. J., & Davidson, Z. E. (2025). Exploring the relationship between vitamin C deficiency and protein-energy malnutrition in adult hospitalised patients: A cross-sectional study. Nutrition & dietetics : the journal of the Dietitians Association of Australia, 82(2), 152–162. https://doi.org/10.1111/1747-0080.12918

Goletti, D., Delogu, G., Matteelli, A., Migliori, G.B. (2022). The role of IGRA in the diagnosis of tuberculosis infection, differentiating from active tuberculosis, and decision making for initiating treatment or preventive therapy of tuberculosis infection. International Journal of Infectious Diseases, Volume 124, Supplement 1, November 2022, Pages S12-S19. https://doi.org/10.1016/j.ijid.2022.02.047

Gomes, J., Silva, J. B., José, C. V., & Ribeiro, H. (2025). An Opinion on the Supplementation of Folic Acid 1 mg + Iron (Ferrous Sulfate) 90 mg in the Prevention and Treatment of Anemia. BioChem, 5(3), 30. https://doi.org/10.3390/biochem5030030

Gomes, J., Silva, J. B., José, C. V., & Ribeiro, H. (2025). An Opinion on the Supplementation of Folic Acid 1 mg + Iron (Ferrous Sulfate) 90 mg in the Prevention and Treatment of Anemia. BioChem, 5(3), 30. https://doi.org/10.3390/biochem5030030

Gordon, R.C., Rose, M.C., Skeaff, S.A., et al. (2009). Iodine supplementation improves cognition in mildly iodine-deficient children. The American Journal of Clinical Nutrition, Volume 90, Issue 5, November 2009, Pages 1264-1271. https://doi.org/10.3945/ajcn.2009.28145

Gorman, S., Bachman, G., Lin, D., Cutler, S., Lopez, G., & Geller, R. (2003). Atropine availability as an antidote for nerve agent casualties: Validated rapid reformulation of high-concentration atropine from bulk powder. Annals of Emergency Medicine, 41(4), 453–456.

Government of Canada. (2024). Oral rehydration solutions. Travel health and safety. Last updated: May 15 2024. Last accessed: December 6 2025. https://travel.gc.ca/travelling/health-safety/rehydration

Grandière Pérez, L., & Brisse, S. (2025). Diphtheria antitoxin treatment: from pioneer to neglected. Memorias do Instituto Oswaldo Cruz, 120, e240214. https://doi.org/10.1590/0074-02760240214

Green, S. M., Tomaszewski, C., Valente, J. H., Lo, B., & Milne, K. (2024). Use of Topical Anesthetics in the Management of Patients With Simple Corneal Abrasions: Consensus Guidelines From the American College of Emergency Physicians. Annals of emergency medicine, 83(5), 477–489. https://doi.org/10.1016/j.annemergmed.2024.01.004

Guaiana, G., Barbui, C., & Hotopf, M. (2007). Amitriptyline for depression. The Cochrane database of systematic reviews, 2007(3), CD004186. https://doi.org/10.1002/14651858.CD004186.pub2

Gulati, S., & Aref, A. A. (2021). Oral acetazolamide for intraocular pressure lowering: balancing efficacy and safety in ophthalmic practice. Expert review of clinical pharmacology, 14(8), 955–961. https://doi.org/10.1080/17512433.2021.1931123

Gupta, B., Mishra, P. A (2021). systematic review and meta-analysis of the use of succinylcholine to facilitate tracheal intubation in neonates. Ain-Shams J Anesthesiol 13, 68 (2021). https://doi.org/10.1186/s42077-021-00185-z

Gupta, S., Kumaravel, J., Prakash, A., & Medhi, B. (2023). Concept of essential medicines and recent updates. Indian journal of pharmacology, 55(1), 1–5. https://doi.org/10.4103/ijp.ijp_94_23

Haby, M.M., Sosa Leon, L.A., Nicholls, R.S., et al. (2020). Systematic review of the effectiveness of selected drugs for preventive chemotherapy for Taenia solium taeniasis. PLOS Neglected Tropical Diseases, 2020. https://doi.org/10.1371/journal.pntd.0007873

Hall, B. S., Bot, C., & Wilkinson, S. R. (2011). Nifurtimox activation by trypanosomal type I nitroreductases generates cytotoxic nitrile metabolites. The Journal of biological chemistry, 286(15), 13088–13095. https://doi.org/10.1074/jbc.M111.230847

Haney, S., Hancox, R.J. (2005). Rapid onset of tolerance to beta-agonist bronchodilation. Respiratory Medicine, Volume 99, Issue 5, p566-571, May 2005. https://doi.org/10.1016/j.rmed.2004.10.014

Hapsoan, J.H., Icanervilia, A.V., Watts, E., et al. (2025). Real-world vaccine effectiveness of typhoid conjugate vaccine in children and adolescents: a systematic review and meta-analysis. Vaccine, Volume 67, 20 November 2025, 127872. https://doi.org/10.1016/j.vaccine.2025.127872

Hatzl, S., Posch, F., Scholz, L., Geiger, C., Kriegl, L.,m et al. (2025). Comparative efficacy and safety of treatment regimens for Pneumocystis jirovecii pneumonia in people living with HIV: a systematic review and network meta-analysis of randomized controlled trials. CMI, Volume 31, Issue 5, May 2025, Pages 713-723. https://doi.org/10.1016/j.cmi.2024.12.024

He, Q., Li. M., Ji, P., et al. (2024). Comparison of drug-induced liver injury risk between propylthiouracil and methimazole: A quantitative systems toxicology approach. Toxicology and Applied Pharmacology, Volume 491, October 2024, 117064. https://doi.org/10.1016/j.taap.2024.117064

Herrick, J. A., Legrand, F., Gounoue, R., Nchinda, G., Montavon, C., Bopda, J., Tchana, S. M., Ondigui, B. E., Nguluwe, K., Fay, M. P., Makiya, M., Metenou, S., Nutman, T. B., Kamgno, J., & Klion, A. D. (2017). Posttreatment Reactions After Single-Dose Diethylcarbamazine or Ivermectin in Subjects With Loa loa Infection. Clinical infectious diseases : an official publication of the Infectious Diseases Society of America, 64(8), 1017–1025. https://doi.org/10.1093/cid/cix016

Hidalgo, J., Tirupathi, R., Ortiz, J. F., Fabara, S. P., Reddy, D., Rabaan, A. A., & Al-Tawfiq, J. A. (2021). 720. Efficacy of Nifurtimox + Eflornithine in the Treatment of African Trypanosomiasis. Systematic Review. Open Forum Infectious Diseases, 8(Suppl 1), 459. https://doi.org/10.1093/ofid/ofab466.917

Hilder, R., Lockwood, D. (2020). The adverse drug effects of dapsone therapy in leprosy: a systematic review; Leprosy Review; 2020; 91; 3; 232-243; DOI: 10.47276/lr.91.3.232

Hirman, M.M., Chong, C.P. (2024). Evaluation of a Guideline on Potassium Chloride Intravenous Supplementation: Safety, Effectiveness and Cost Implications. Malaysian Journal of Medicine and Health Sciences 20:64-74. eISSN 2636-9346. https://www.researchgate.net/publication/391265943_Evaluation_of_a_Guideline_on_Potassium_Chloride_Intravenous_Supplementation_Safety_Effectiveness_and_Cost_Implications

HIVinfo NIH. (2024). Pneumocystis Pneumonia. Guidelines. National Institute of Health (NIH), US. Last updated: September 092025. Last accessed: November 29 2025. https://clinicalinfo.hiv.gov/en/guidelines/hiv-clinical-guidelines-adult-and-adolescent-opportunistic-infections/pneumocystis

Hoeltzenbein, M., Beck, E., Fiets, A.K., et al. (2017). Pregnancy Outcome After First Trimester Use of Methyldopa: A Prospective Cohort Study. Hypertension, Volume 70, Number 1. https://doi.org/10.1161/HYPERTENSIONAHA.117.09110

Hoffmann, F., Bantel, C., von Rosen, F. T., & Jobski, K. (2020). Regional Differences in Prescribing Patterns of Metamizole in Germany Based on Data from 70 Million Persons. International Journal of Environmental Research and Public Health, 17(11), 3892. https://doi.org/10.3390/ijerph17113892

Honorato, S. B., da Silva, C. C., de Oliveira, Y. S., Mendonça, J. S., Boechat, N., Ellena, J., & Ayala, A. P. (2016). On the Thermal Stability of the Diethylcarbamazine-Fortified Table Salt Used in the Control of Lymphatic Filariasis. Journal of pharmaceutical sciences, 105(8), 2437–2443. https://doi.org/10.1016/j.xphs.2016.06.003

Honore, P.M., Barreto Gutierrez, L. & Spapen, H.D. (2019). Renal protection in sepsis: Is hypertonic sodium (lactate) the solution?. Ann. Intensive Care 9, 28 (2019). https://doi.org/10.1186/s13613-019-0505-z

Hou, W. S., Tsai, J. P., Chiu, Y. H., & Lu, M. L. (2022). Carbamazepine-induced DRESS Syndrome: A Rare Delayed Hypersensitivity Reaction. Journal of psychiatric practice, 28(2), 166–169. https://doi.org/10.1097/PRA.0000000000000619

Hovgaard, H.L., Juhl-Olsen, P. (2021). Suxamethonium-Induced Hyperkalemia: A Short Review of Causes and Recommendations for Clinical Applications. Critical Care Research and Practice, 25 February 2021. https://doi.org/10.1155/2021/6613118

Inshutiyimana, S., Aleu, M.M., Abdinoor, M.A., Janoowalla, M.M., Norhayati, N. (2024). Diloxanide in amoebiasis management: Unravelling the mechanism of action and effectiveness. BCPT, Volume 136, Issue 1, January 2025, e14106. https://doi.org/10.1111/bcpt.14106

Institut für medizinische und pharmazeutische Prüfungsfrgaen (IMPP). Arzneistoffliste Version 2.0. Last updated: November 2024. Last accessed: December 8 2025. https://www.impp.de/files/PDF/Gegenstandskataloge/Medizin/Arzneistoffliste_M2_V2.0_Nov%202024.pdf

International Waldenstrom's Macroglobulinemia Foundation (IWMF). (2021). Cyclophosphamide Fact Sheet. iwmf.com 941-927-4963. Last updated: February 18 2021. Last accessed: November 30 2025. https://iwmf.com/wp-content/uploads/2021/03/English_Cyclophosphamide_Fact_Sheet-English_2.18-1.pdf

Jaiswal, A. K., Rodrigues Gomes, L. G., Ferreira Maciel de Oliveira, A., de Castro Soares, S., & Azevedo, V. (2025). The Critical Role of Penicillin in Syphilis Treatment and Emerging Resistance Challenges. Diseases, 13(2), 41. https://doi.org/10.3390/diseases13020041

Janier, M., Unemo, M., Dupin, N., Tiplica, G. S., Potočnik, M., & Patel, R. (2021). 2020 European guideline on the management of syphilis. Journal of the European Academy of Dermatology and Venereology : JEADV, 35(3), 574–588. https://doi.org/10.1111/jdv.16946

Jayawardana, S., Forman, R., Johnston-Webber, C., et al. (2021). Global consumption of prescription opioid analgesics between 2009-2019: a country-level observational study. eClinicalMedicine, Volume 42, 101198, December 2021. https://doi.org/10.1016/j.eclinm.2021.101198

Jiang, S., & Gunther, M. (2025). A Critical Reappraisal of Haloperidol for Delirium Management in the Intensive Care Unit: Perspective from Psychiatry. Journal of Clinical Medicine, 14(2), 438. https://doi.org/10.3390/jcm14020438

Joint British Diabetes Societies for inpatient care (JBDS-IP). (2023). The Hospital Management of Hypoglycaemia in Adults with Diabetes Mellitus. JBDS-IP. Last updated: January 2023. Last accessed: November 20 2025. https://abcd.care/sites/default/files/site_uploads/JBDS_Guidelines_Current/JBDS_01_Hypo_Guideline_with_QR_code_January_2023.pdf

Jones, A.J., Federspiel, J.J., Ahizechukwu, C.E. (2022). Preventing postpartum hemorrhage with combined therapy rather than oxytocin alone. American Journal of Obstetrics & Gynecology MFM, Volume 5, Issue 2, Supplement, February 2023, 100731. https://doi.org/10.1016/j.ajogmf.2022.100731

Jones, D.W., Ferdinand, K.C., Taler, S.J., et al. (2025). 2025 AHA/ACC/AANP/AAPA/ABC/ACCP/ACPM/AGS/AMA/ASPC/NMA/PCNA/SGIM Guideline for the Prevention, Detection, Evaluation and Management of High Blood Pressure in Adults: A Report of the American College of Cardiology/American Heart Association Joint Committee on Clinical Practice Guidelines. Circulation, Volume 152, Number 11. https://doi.org/10.1161/CIR.0000000000001356

Jrayed, R.H., Alshangiti, S.A., Alhassan, R.A., Alanzi, N.O., et al. (2024). Therapeutic Effectiveness and Safety of Activated Charcoal in Poisoning Management in Emergency Settings: A Systematic Review and Meta-analysis. Journal of Advanced Trends in Medical Research 1(3):p 929-937, Jul–Sep 2024. DOI: 10.4103/ATMR.ATMR_158_24

Jung, HY., Jeon, Y., Park, Y. et al. (2019). Better Quality of Life of Peritoneal Dialysis compared to Hemodialysis over a Two-year Period after Dialysis Initiation. Sci Rep 9, 10266 (2019). https://doi.org/10.1038/s41598-019-46744-1

Jyothirmai, C., Kumar, K.., Rajendar, K. (2022). Comparative Efficacy and Safety of Ibuprofen, Acetaminophen, And Naproxen in The Management of Pediatric Fever and Pain. European Journal of Cardiovascular Medicine, Volume 12, Issue 4, Pages 386-390. DOI: 10.61336/ejcm/22-10-04

Kaasch, A.J., Fätkenheuer, G., Prinz-Langenohl, R. et al. (2015). Early oral switch therapy in low-risk Staphylococcus aureus bloodstream infection (SABATO): study protocol for a randomized controlled trial. Trials 16, 450. https://doi.org/10.1186/s13063-015-0973-x

Kaewkungwal J, Roobsoong W, Lawpoolsri S, Nguitragool W, Thammapalo S, Prikchoo P, Khamsiriwatchara A, Pawarana R, Jarujareet P, Parker DM, Sripoorote P, Kengganpanich M, Ngamjarus C, Sattabongkot J, Cui L. (2024). Effectiveness, Safety, and Acceptability of Primaquine Mass Drug Administration in Low-Endemicity Areas in Southern Thailand: Proof-of-Concept Study. JMIR Public Health Surveill 2024;10:e51993. doi: 10.2196/51993

Kahaly, G. J., Bartalena, L., Hegedüs, L., Leenhardt, L., Poppe, K., & Pearce, S. H. (2018). 2018 European Thyroid Association Guideline for the Management of Graves' Hyperthyroidism. European thyroid journal, 7(4), 167–186. https://doi.org/10.1159/000490384

Kaiti, R., Shyangbo, R., & Sharma, I. P. (2022). Role of Atropine in the control of Myopia Progression- A Review. Beyoglu eye journal, 7(3), 157–166. https://doi.org/10.14744/bej.2022.07742

Kalman, D., Hewlings, S., Madelyn-Adjei, A., & Ebersole, B. (2025). Dietary Heme Iron: A Review of Efficacy, Safety and Tolerability. Nutrients, 17(13), 2132. https://doi.org/10.3390/nu17132132

Kandler MR, Mah GT, Tejani AM, Stabler SN, Salzwedel DM. (2022). Hydralazine for essential hypertension. Cochrane Database of Systematic Reviews 2022, Issue 3. Art. No.: CD004934. DOI: 10.1002/14651858.CD004934.pub4.

Kang, M.S., Chang, M.C. & Kwak, S. (2023). Usefulness of barium sulfate and iohexol as contrast agents for VFSS in visualizing components of swallowing predictable of poor outcomes. Sci Rep 13, 21556 (2023). https://doi.org/10.1038/s41598-023-46297-4

Kara, A.O., Beshr, M.S., Abdelaziz, A.M., Alzubi, A.S., et al. (2025). Efficacy and Safety of Protamine Use following Transcatheter Aortic Valve Implantation for Minimizing Post-Procedural Bleeding Risk: A Systematic Review and Meta-Analysis. Cardiology. https://doi.org/10.1159/000549115

Kardalas, E., Paschou, S. A., Anagnostis, P., Muscogiuri, G., Siasos, G., & Vryonidou, A. (2018). Hypokalemia: a clinical update. Endocrine connections, 7(4), R135–R146. https://doi.org/10.1530/EC-18-0109

Kavanagh, K. (2025). The rise of ‘nightmare bacteria’: antimicrobial resistance in five charts. Nature 646, 526-527. https://doi.org/10.1038/d41586-025-03218-x

Kedia NY, Malaviya AN. (2025). The Cyclophosphamide Story: From Killer Gas to Life-saving Drug for Cancers and Inflammatory Rheumatic and Musculoskeletal Diseases (I-RMDs). Indian Journal of Rheumatology. 2025;0(0). doi:10.1177/09733698251355347

Kendrick, T., Taylor, D., Johnson, C.F. (2019). Which first-line antidepressant? British Journal of General Practice 2019; 69 (680): 114-115. https://doi.org/10.3399/bjgp19X701405

Khandelia, P., Yadav, S., Singh, P. (2023). An overview of the BCG vaccine and its future scope. Indian Journal of Tuberculosis, Volume 70, Supplement 1, 2023, Pages S14-S23. https://doi.org/10.1016/j.ijtb.2023.05.012

Khinda, V., Rao, D., & Sodhi, S. P. S. (2023). Nitrous Oxide Inhalation Sedation Rapid Analgesia in Dentistry: An Overview of Technique, Objectives, Indications, Advantages, Monitoring, and Safety Profile. International journal of clinical pediatric dentistry, 16(1), 131–138. https://doi.org/10.5005/jp-journals-10005-1807

Khoo, A.B., Ali, F.R., Yiu, Z.Z., Ferguson, J.E. (2016). Carbamazepine induced Stevens-Johnson syndrome. BMJ Case Reports. https://doi.org/10.1136/bcr-2016-214926

Kido, K. (2025). Evaluating the Lidocaine's Initial Dosing in Patients With Ventricular Arrhythmias and Heart Failure Admitted in Intensive Care Units. Clinical Cardiology, Volume 48, Issue 4, April 2025, e70126. https://doi.org/10.1002/clc.70126

Kienitz, R., Kay, L., Beuchat, I., Gelhard, S., von Brauchitsch, S., Mann, C., Lucaciu, A., Schäfer, J. H., Siebenbrodt, K., Zöllner, J. P., Schubert-Bast, S., Rosenow, F., Strzelczyk, A., & Willems, L. M. (2022). Benzodiazepines in the Management of Seizures and Status Epilepticus: A Review of Routes of Delivery, Pharmacokinetics, Efficacy, and Tolerability. CNS drugs, 36(9), 951–975. https://doi.org/10.1007/s40263-022-00940-2

Kim, W. O., Song, B. M., & Kil, H. K. (2012). Efficacy and safety of a lidocaine/tetracaine medicated patch or peel for dermatologic procedures: a meta-analysis. Korean journal of anesthesiology, 62(5), 435–440. https://doi.org/10.4097/kjae.2012.62.5.435

Kim, Y. A., Park, Y. S., Youk, T., Lee, H., & Lee, K. (2018). Correlation of Aminoglycoside Consumption and Amikacin- or Gentamicin-Resistant Pseudomonas aeruginosa in Long-Term Nationwide Analysis: Is Antibiotic Cycling an Effective Policy for Reducing Antimicrobial Resistance?. Annals of laboratory medicine, 38(2), 176–178. https://doi.org/10.3343/alm.2018.38.2.176

Kivimäki, M., Vineis, P., Brunner, E.J. (2015). How can we reduce the global burden of disease?. The Lancet. Volume 386, Issue 10010p2235-2237, December 05, 2015. https://doi.org/10.1016/S0140-6736(15)00129-4

Kothari, D., Kothari, S., & Agrawal, J. (2012). Potassium chloride: A high risk drug for medication error. Indian journal of anaesthesia, 56(1), 90–91. https://doi.org/10.4103/0019-5049.93357

Kovich, H., Kim, W., Quaste, A.M. (2023). Pharmacologic Treatment of Depression. Am Fam Physician. 2023;107(2):173-181.

Krismawati, H., Harianja, M., Oktavian, A., et al. (2025). Challenges associated with dapsone for leprosy treatment in Indonesia - urgent need for access to alternative antimicrobial drugs. The Lancet Regional Health - Southeast Asia. Volume 34, March 2025, 100555. https://doi.org/10.1016/j.lansea.2025.100555

Kuemmerle, A., Schmid, C., Bernhard, S., Kande, V., Mutombo, W., Ilunga, M., Lumpungu, I., Mutanda, S., Nganzobo, P., Tete, D. N., Kisala, M., Burri, C., Blesson, S., & Valverde Mordt, O. (2021). Effectiveness of Nifurtimox Eflornithine Combination Therapy (NECT) in T. b. gambiense second stage sleeping sickness patients in the Democratic Republic of Congo: Report from a field study. PLoS neglected tropical diseases, 15(11), e0009903. https://doi.org/10.1371/journal.pntd.0009903

Kumar, M., Chawla, R., & Goyal, M. (2015). Topical anesthesia. Journal of anaesthesiology, clinical pharmacology, 31(4), 450–456. https://doi.org/10.4103/0970-9185.169049

Kumari S, Kumar V, Tiwari RK, Ravidas V, Pandey K, Kumar A. Amphotericin B: A drug of choice for Visceral Leishmaniasis. Acta Trop. 2022 Nov;235:106661. doi: 10.1016/j.actatropica.2022.106661. Epub 2022 Aug 20. PMID: 35998680.

La Vecchia, A., Fontani, E., Catania, M.A., Pietravalle A., et al. (2025). WHO guideline adherence and AWaRe antibiotic use in severe childhood pneumonia in Ethiopia: Associations with treatment outcomes. International Journal of Infectious Diseases, Volume 161, December 2025, 108116. https://doi.org/10.1016/j.ijid.2025.108116

Lago-Deibe, F. I., Valladares-Cabaleiro, M., Fernández-Domínguez, M. J., Fernández-Fernández, I., Clavería, A., Rodríguez-Pastoriza, S., Roca-Pardinas, J., & Martín-Miguel, M. V. (2022). Effectiveness and safety of tetanus vaccine administration by intramuscular vs. subcutaneous route in anticoagulated patients: Randomized clinical trial in primary care. Frontiers in medicine, 9, 1054988. https://doi.org/10.3389/fmed.2022.1054988

Laing, D., Walsh, P.G., Alsweiler, J.M. (2024). Diazoxide for Severe or Recurrent Neonatal Hypoglycemia. JAMA Netw Open. 2024;7;(6):e2415764. doi:10.1001/jamanetworkopen.2024.15764+

Lee, A.G., Charoenkijkajorn, C., Pakravan, M., et al. (2025b). Acetazolamide. Complications in Ophthalmology. American Academy of Phthalmology (AAE). Last updated: October 6 2025. Last accessed: December 7 2025. https://eyewiki.org/Acetazolamide_Complications_in_Ophthalmology

Lee, P.T., Huang, M.H., Sung, P.S., et al. (2025a). Improvement of Oral Anticoagulant Prescription and Long‐Term Clinical Outcomes of Patients With Atrial Fibrillation After Implementation of a Clinical Decision Support System in Outpatient Practice. JAHA, Volume 14, Number 15. https://doi.org/10.1161/JAHA.124.038640

Legese N, Aferu T, Kassa T. (2021). Cost-Effectiveness Analysis of Metronidazole versus Metronidazole with Diloxanide Furoate in the Treatment of Amoebiasis in Ethiopia. Clinicoecon Outcomes Res. 2021;13:611-617. https://doi.org/10.2147/CEOR.S312821

Lehnerer, S., Jacobi, J., Schilling, R., Grittner, U., Marbin, D., Gerischer, L., Stascheit, F., Krause, M., Hoffmann, S., & Meisel, A. (2022). Burden of disease in myasthenia gravis: taking the patient's perspective. Journal of neurology, 269(6), 3050–3063. https://doi.org/10.1007/s00415-021-10891-1

Lejon, V., Lindner, A.K., Franceo, J.R. (2025). Human African trypanosomiasis. The Lancet, Volume 405, Issue 10482, p937-950, March 15, 2025. https://doi.org/10.1016/S0140-6736(25)00107-2

Levecke B, Montresor A, Albonico M, Ame SM, Behnke JM, et al. (2014) Assessment of Anthelmintic Efficacy of Mebendazole in School Children in Six Countries Where Soil-Transmitted Helminths Are Endemic. PLoS Negl Trop Dis 8(10): e3204. doi:10.1371/journal.pntd.0003204

Li M, Ka D, Chen Q. Disparities in availability of new cancer drugs worldwide: 1990-2022. BMJ Global Health. 2024;9:e015700. https://doi.org/10.1136/bmjgh-2024-015700

Liabsuetrakul T, Choobun T, Peeyananjarassri K, Islam QM. (2018). Prophylactic use of ergot alkaloids in the third stage of labour. Cochrane Database of Systematic Reviews 2018, Issue 6. Art. No.: CD005456. DOI: 10.1002/14651858.CD005456.pub3.

Linde, K., & Rossnagel, K. (2017). WITHDRAWN: Propranolol for migraine prophylaxis. The Cochrane database of systematic reviews, 2(2), CD003225. https://doi.org/10.1002/14651858.CD003225.pub3

Liu, B., Zhou, H., Tan, L. et al. (2024). Exploring treatment options in cancer: tumor treatment strategies. Sig Transduct Target Ther 9, 175. https://doi.org/10.1038/s41392-024-01856-7

Liu, H.Y., Han, Y., Chen, X.S., Bai, L., Gui, S.P., Wu, P., Yin, Y.P. (2017). Comparison of efficacy of treatments for early syphilis: A systematic review and network meta-analysis of randomized controlled trials and observational studies. PLOS One, June 28 2017. https://doi.org/10.1371/journal.pone.0180001

Liu, Q., Yin, W., Yang, L., Jia, J., Zhao, L., Yao, H., Xu, B., Lei, M., Fan, S., & Bu, H. (2022). Zhonghua wei zhong bing ji jiu yi xue, 34(7), 736–739. https://doi.org/10.3760/cma.j.cn121430-20210827-01289

Lonjaret, L., Lairez, O., Minville, V., & Geeraerts, T. (2014). Optimal perioperative management of arterial blood pressure. Integrated blood pressure control, 7, 49–59. https://doi.org/10.2147/IBPC.S45292

Løvås, K., & Husebye, E. S. (2008). Replacement therapy for Addison's disease: recent developments. Expert opinion on investigational drugs, 17(4), 497–509. https://doi.org/10.1517/13543784.17.4.497

Lovell, K.K., Momin, R.I., Sangha, H.S. et al. (2024). Dapsone Use in Dermatology. Am J Clin Dermatol 25, 811–822. https://doi.org/10.1007/s40257-024-00879-8

Ludwig, W.D., Mühlbauer, B., Seifert, R. (2025). Arzneiverordnungs-Report 2024. 1st edition. Springer Berlin, Heidelberg. ISBN: 978-3-662-70594-0. https://doi.org/10.1007/978-3-662-70594-0

Luo, H., Xu, L., Chen, Y. (2023). Drug resistance and susceptibility of amikacin in children with extended-spectrum beta-lactamase-producing Enterobacterales: a systematic review with meta-analysis. Diagnostic Microbiology and Infectious Disease. Volume 106, Issue 4, August 2023, 115956. https://doi.org/10.1016/j.diagmicrobio.2023.115956

Lykkesfeldt, J. (2020). On the effect of vitamin C intake on human health: How to (mis)interprete the clinical evidence. Redox Biology, Volume 34, July 2020, 101532. https://doi.org/10.1016/j.redox.2020.101532

Lyu X, Zhao C, Yan Z, Hua H. (2016). Efficacy of nystatin for the treatment of oral candidiasis: a systematic review and meta-analysis. Drug Des Devel Ther. 2016;10:1161-1171. https://doi.org/10.2147/DDDT.S100795

Ma, L. H., Jia, L., & Bai, L. (2023). Safety outcomes of salbutamol: A systematic review and meta-analysis. The clinical respiratory journal, 17(12), 1254–1264. https://doi.org/10.1111/crj.13711

Madukwe, J.C. (2022). For novel cardiovascular therapy discovery and development. Trends in Pharmacological Sciences, Volume 43, Issue 11, 885 - 887. https://doi.org/10.1016/j.tips.2022.09.002

Maertens J, Pagano L, Azoulay E, Warris A. Liposomal amphotericin B-the present. J Antimicrob Chemother. 2022 Nov 25;77(Suppl_2):ii11-ii20. doi: 10.1093/jac/dkac352. PMID: 36426672; PMCID: PMC9693760.

Maggi, L., & Mantegazza, R. (2011). Treatment of myasthenia gravis: focus on pyridostigmine. Clinical drug investigation, 31(10), 691–701. https://doi.org/10.2165/11593300-000000000-00000

Mah GT, Tejani AM, Musini VM. (2022). Methyldopa for primary hypertension. Cochrane Database of Systematic Reviews 2022, Issue 3. Art. No.: CD003893. DOI: 10.1002/14651858.CD003893.pub3.

Mahfuz, M., Tariful Islam Khan, A. K. M., & Yunus, M. (2024). Development of oral rehydration salt solution: A triumph of medical science. The Indian journal of medical research, 160(1), 6–9. https://doi.org/10.25259/IJMR_989_2024

Maier, C., Hildebrandt, J., Klinger, R., Henrich-Eberl, C., Lindena, G., & MONTAS Study Group (2002). Morphine responsiveness, efficacy and tolerability in patients with chronic non-tumor associated pain - results of a double-blind placebo-controlled trial (MONTAS). Pain, 97(3), 223–233. https://doi.org/10.1016/S0304-3959(02)00020-9

Manno E. M. (2011). Status epilepticus: current treatment strategies. The Neurohospitalist, 1(1), 23–31. https://doi.org/10.1177/1941875210383176

Markota, M., Morgan, R.J. & Leung, J.G. (2024). Updated rationale for the initial antipsychotic selection for patients with schizophrenia. Schizophr 10, 74 (2024). https://doi.org/10.1038/s41537-024-00492-y

Marques, L., & Vale, N. (2022). Salbutamol in the Management of Asthma: A Review. International journal of molecular sciences, 23(22), 14207. https://doi.org/10.3390/ijms232214207

Martinez, L., Cords, O., Liu, Q., et al. (2022). Infant BCG vaccination and risk of pulmonary and extrapulmonary tuberculosis throughout the life course: a systematic review and individual participant data meta-analysis. The Lancet Global Health, Volume 10, Issue 9, e1307-e1316, September 2022. https://doi.org/10.1016/S2214-109X(22)00283-2

Masserey, T., Braunack-Mayer, L., Miller, R.S., et al. (2025). A roadmap for understanding sulfadoxine-pyrimethamine in malaria chemoprevention. Parasitology. 2025;152(2):133-142. doi:10.1017/S0031182025000071

Mayans, L., Walling, A. (2018). Acute Migraine Headache: Treatment Strategies. Am Fam Physician. 2018;97(4):243-251

McGlone, A., Cranswick, N. (2008). Evidence Behind the WHO Guidelines: Hospital Care for Children: What is the Evidence of Safety of Gentamicin use in Children?, Journal of Tropical Pediatrics, Volume 54, Issue 5, October 2008, Pages 291–293, https://doi.org/10.1093/tropej/fmn059

Medecins sans frontieres (MSF). (2025a). Dehydration. Clinical guidelines - Diagnosis and treatment manual. MSF. Last updated: 2025. Last accessed: November 24 2025. https://medicalguidelines.msf.org/en/node/181

Medecins sans frontieres (MSF). (2025b). POTASSIUM CHLORIDE 10% = KCl 10% injectable. Essential drugs. MSF. Last updated: 2025. Last accessed: November 25 2025. https://medicalguidelines.msf.org/en/node/1031

Medecins sans frontieres (MSF). (2025d). CO-TRIMOXAZOLE = SULFAMETHOXAZOLE (SMX)/TRIMETHOPRIM (TMP) oral. Last updated: 2025. Last accessed: November 30 2025. https://medicalguidelines.msf.org/en/node/387

Medicines and Healthcare products Regulatory Agency (MHRA). (2020). New restrictions introduced on sales of stimulant laxatives to counter risks from overuse. Press release. Last updated: August 18 2020. Last accessed: December 6 2025. https://www.gov.uk/government/news/new-restrictions-introduced-on-sales-of-stimulant-laxatives-to-counter-risks-from-overuse

Medicines and Healthcare products Regulatory Agency. (2014). Codeine for analgesia: restricted use in children because of reports of morphine toxicity. Drug Safety Updated. Government UK. Last updated: December 11 2014. Last accessed: December 7 2025. https://www.gov.uk/drug-safety-update/codeine-for-analgesia-restricted-use-in-children-because-of-reports-of-morphine-toxicity

Mejia Torres, R. E., Banegas, E. I., Mendoza, M., Diaz, C., Bucheli, S. T., Fontecha, G. A., Alam, M. T., Goldman, I., Udhayakumar, V., & Zambrano, J. O. (2013). Efficacy of chloroquine for the treatment of uncomplicated Plasmodium falciparum malaria in Honduras. The American journal of tropical medicine and hygiene, 88(5), 850–854. https://doi.org/10.4269/ajtmh.12-0671

Miao, Y., Xu, Y., Teng, P. (2022). Efficacy of propylthiouracil in the treatment of pregnancy with hyperthyroidism and its effect on pregnancy outcomes: A meta-analysis. PLOS One, March 10 2022. https://doi.org/10.1371/journal.pone.0265085

Ministerio de Salud y Protección Social (Minsalud). (2022). Albendazole and Mebendazole Are Safe Antiparasitic Drugs. Press release Press Release No. 390, 2022. Last updated: July 14 2022. Last accessed: November 29 2025. https://www.minsalud.gov.co/English/Paginas/Albendazole-and-Mebendazole-Are-Safe-Antiparasitic-Drugs.aspx

Misera, N., & Seifert, R. (2024). Presentation of the obsolete drug reserpine in three German-language pharmacology textbooks. Naunyn-Schmiedeberg's archives of pharmacology, 397(6), 4381–4401. https://doi.org/10.1007/s00210-023-02877-9

Modi, N., Maggs, A.F., Clarke, C. et al. (1998). Gentamicin concentration and toxicity. The Lancet, Volume 352, Issue 9121, p70, July 04, 1998. https://doi.org/10.1016/S0140-6736(05)79557-X

Mohite, D., Saner, K., Sable, P.N., Parmar, N., Sirvi, M., Mohite, V. (2024). Promoting Rational Use of Medicines: A Multifaceted Approach. Int. J. of Pharm. Sci., 2024, Vol 2, Issue 12, 2677-2684. https://doi.org/10.5281/zenodo.14539011

Molloy, S.F., Kanyama, C., Heyderman, R.S., Loyse, A., Kouanfack, C., et al. (2018). Antifungal Combinations for Treatment of Cryptococcal Meningitis in Africa. N Engl J Med 2018; Vol. 378 No. 11, 378:1004-1017. DOI: 10.1056/NEJMoa1710922.

Mombo-Ngoma, G., Ramharter, M., Zoleko Manego, R. et al. (2025). Making the most of existing antimalarial medicines: a single dose cure with sulfadoxine–pyrimethamine plus artesunate–pyronaridine. Malar J 24, 300. https://doi.org/10.1186/s12936-025-05559-4

Montazerlotfelahi, H., Haj Mohamad Ebrahim Ketabforoush, A., Tavakol, M. et al. (2024). Safety and efficacy of levetiracetam and carbamazepine monotherapy in the management of pediatric focal epilepsy: a randomized clinical trial. Naunyn-Schmiedeberg's Arch Pharmacol 397, 5233–5240 (2024). https://doi.org/10.1007/s00210-024-02954-7

Morales-Olivas, F.J. (2024). Diuretics use in the management of hypertension. Hipertensión y Riesgo Vascular, Volume 41, Issue 3, July–September 2024, Pages 186-193. https://doi.org/10.1016/j.hipert.2024.03.004

Mouawad, L. (2022). Review—New Approaches for the Detection of Lithium Carbonate: The First Line Treatment for Bipolar Disorder. ECS Advances, 1 036501. DOI 10.1149/2754-2734/ac8065

Murdan, S., Wei, L., van Riet-Nales, D.A., et al. (2023). Association between culture and the preference for, and perceptions of, 11 routes of medicine administration: A survey in 21 countries and regions. Exploratory Research in Clinical and Social Pharmacy, Volume 12, December 2023, 100378. https://doi.org/10.1016/j.rcsop.2023.100378

Murray H. W. (2012). Leishmaniasis in the United States: treatment in 2012. The American journal of tropical medicine and hygiene, 86(3), 434–440. https://doi.org/10.4269/ajtmh.2012.11-0682

Musa, A.M., Mbui, J., Mohammed, R., Olobo, J., et al. (2023). Paromomycin and Miltefosine Combination as an Alternative to Treat Patients With Visceral Leishmaniasis in Eastern Africa: A Randomized, Controlled, Multicountry Trial, Clinical Infectious Diseases, Volume 76, Issue 3, 1 February 2023, Pages e1177–e1185, https://doi.org/10.1093/cid/ciac643

Najjar, R. (2024). Clinical applications, safety profiles, and future developments of contrast agents in modern radiology: A comprehensive review. iRadiology, Volume 2, Issue 5, October 2024, Pages 430-468. https://doi.org/10.1002/ird3.95

National Health Service (NHS). (2022b). Iron Deficiency Anaemia (IDA) in Adults: Oral and Intravenous Iron Therapy Treatment (662). GGC - Clinical Guidelines. NHS Highland, Scotland. Last updated: May 12 2022. Last accessed: November 30 2025. https://www.rightdecisions.scot.nhs.uk/ggc-clinical-guidelines/haematology/nutritional-deficiencies/iron-deficiency-anaemia-ida-in-adults-oral-and-intravenous-iron-therapy-treatment-662/

National Health Service (NHS). (2024a). Hypoglycaemia in Adults with Diabetes Clinical Guideline. V7.0. NHS, Royal Cornwall Hospitals. Last udpated: December 2024. Last accessed: November 2025. https://doclibrary-rcht.cornwall.nhs.uk/GET/d10367432

National Health Service (NHS). (2024b). Dental Abscess (Antimicrobial). TAM (Treatments and Medicines) NHS Highland, Scotland. Last updated: August 22 2024. Last accessed: November 29 2025. https://www.rightdecisions.scot.nhs.uk/tam-treatments-and-medicines-nhs-highland/adult-therapeutic-guidelines/antimicrobial-guidance/dental-antimicrobial/dental-abscess-antimicrobial/

National Health Service (NHS). (2025a). Intravenous Fluid Therapy for Adults in Hospital Clinical Guideline. V3.0. NHS, Royal Cornwall Hospitals. Last updated: August 2025. Last accessed: November 20 2025. https://doclibrary-rcht.cornwall.nhs.uk/DocumentsLibrary/RoyalCornwallHospitalsTrust/Clinical/CriticalCareAndResuscitation/IntravenousFluidTherapyForAdultsInHospitalClinicalGuideline.pdf

National Health Service (NHS). (2025c). Guideline for the Management of Vitamin D Deficiency in Adults from 18 years of age in Primary Care. NHS, BLMK Area Prescribing Committee. Last updated: May 2025. Last accessed: December 7 2025. https://medicines.bedfordshirelutonandmiltonkeynes.icb.nhs.uk/wp-content/uploads/2025/05/Vitamin-D-Deficiency-in-Adults-in-Primary-Care-May-2025.pdf

National Health Service Scotland (NHS Scotland). (2020). Sodium Lactate Intravenous Infusion, Compound (Hartmann’s Solution for Injection; Ringer-Lactate Solution for Injection). Scotland national midwifery formulary. v2. Last updated: January 27 2020. Last accessed: November 20 2025. https://www.rightdecisions.scot.nhs.uk/media/1276/compound-sodium-lactate-m-ex-v2.pdf

National Health Service. (2023). Clinical Guideline - Amickacin dosing guidelines in Adults aged 16 years and over. NHS, Greater Glasgow and Clyde. Last updated: October 19 2023. Last accessed: November 24 2025. https://www.rightdecisions.scot.nhs.uk/media/rl0e5l0x/amikacin-dosing-guidelines-in-adults-1099.pdf

National Institute for Health and Care Excellence (NICE). (2018). Sore throat (acute): antimicrobial prescribing. NICE guideline, 2018. NG 84. Last updated: January 26 2018. Last accessed: November 29 2025. https://www.nice.org.uk/guidance/ng84/chapter/recommendations

National Institute for Health and Care Excellence (NICE). (2022b). Gout: diagnosis and management. NICE guideline. Reference number: NG219. Last updated: June 9 2022. Last accessed: December 3 2025. https://www.nice.org.uk/guidance/ng219/chapter/Recommendations

National Institute for Health and Care Excellence (NICE). (2022c). Depression in adults: treatment and management. NICE guideline. Reference number: NG222. Last updated: June 29 2022. Last accessed: December 3 2025. https://www.nice.org.uk/guidance/ng222/chapter/recommendations

National Institute for Health and Care Excellence (NICE). (2025a). Bipolar disorder: assessment and management. Clinical guideline. Reference number: CG185. Last udpated: September 2 2025. Last accessed: December 3 2025. https://www.nice.org.uk/guidance/cg185/chapter/Recommendations

National Institute of Allergy and Infectious Diseases (NIAID). (2024). Antiretroviral Drug Discovery and Development. Last updated: February 5 2024. Last accessed: November 13 2025. https://www.niaid.nih.gov/diseases-conditions/antiretroviral-drug-development

Natov, P.S., Schwartz, A.R. (2025). Hydrochlorothiazide Is America's Most Popular Thiazide, But Should It Be? JAHA, Volume 14, Number 6. https://doi.org/10.1161/JAHA.124.035573

Nature. (2025). The Future of Medicine. Nat Med 31, 1 (2025). https://doi.org/10.1038/s41591-024-03464-y

Negarandeh, R., Salehifar, E., Saghafi, F. et al. Evaluation of adverse effects of chemotherapy regimens of 5-fluoropyrimidines derivatives and their association with DPYD polymorphisms in colorectal cancer patients. BMC Cancer 20, 560 (2020). https://doi.org/10.1186/s12885-020-06904-3

Neunert, C., Terrell, D.R., Arnold, D.M., et al. (2019). American Society of Hematology 2019 guidelines for immune thrombocytopenia. Blood Advances, Volume 3, Issue 23, December 10 2019. https://doi.org/10.1182/bloodadvances.2019000966

Nevitt SJ, Marson AG, Tudur Smith C. (2018). Carbamazepine versus phenobarbitone monotherapy for epilepsy: an individual participant data review. Cochrane Database of Systematic Reviews 2018, Issue 10. Art. No.: CD001904. DOI: 10.1002/14651858.CD001904.pub4. Accedida el 03 de diciembre de 2025.

New South Wales (NSW) Government. (2023). Paracetamol. Medication Safety. NSW Government, Clinical Excellence Commission, Australia. Last updated: February 26 2024. Last accessed: December 4 2025. https://www.cec.health.nsw.gov.au/__data/assets/pdf_file/0008/933695/Printable-HRM-Standard-Paracetamol.PDF

Nirk, E.L., Reggiori, F., Mauthe, M. (2020). Hydroxychloroquine in rheumatic autoimmune disorders and beyond. EMBO Mol Med 2020, 12: e12476. https://doi.org/10.15252/emmm.202012476

O'Brien, J., Dong, B.J., Coleman, R.L., et al. (1997). A 5-Year Retrospective Review of Adverse Drug Reactions and Their Risk Factors in Human Immunodeficiency Virus—Infected Patients Who Were Receiving Intravenous Pentamidine Therapy for Pneumocystis carinii Pneumonia, Clinical Infectious Diseases, Volume 24, Issue 5, May 1997, Pages 854–859, https://doi.org/10.1093/clinids/24.5.854

Oda, Y., Yamasaki, H., Osawa, T., Igarashi, M., Ishizu, T. (2025). Rescuing Protamine Anaphylaxis Refractory to Adrenaline Using Extracorporeal Membrane Oxygenation. JACC: Case Reports, Volume 30, Issue 6, Part 1, 19 March 2025, 102966. https://doi.org/10.1016/j.jaccas.2024.102966

Okuno, H., Satoh, H., Morino, S. (2022). Characteristics and incidence of vaccine adverse events after Bacille Calmette–Guérin vaccination: A national surveillance study in Japan from 2013 to 2017. Vaccine, Volume 40, Issue 33, 5 August 2022, Pages 4922-4928. https://doi.org/10.1016/j.vaccine.2022.05.055

Omar Ibrahim, I., Perrot, C., Roumes, H. et al. (2025). Positive impact of sodium L-lactate supplementation on blood acid-base status in preterm newborns. Pediatr Res 98, 611–620 (2025). https://doi.org/10.1038/s41390-025-03963-9

Oncology Nursing Society (ONS). (2025). Oncology Drug Reference Sheet: Doxorubicin Hydrochloride. Last updated: May 20 2025. Last accessed: November 30 2025. https://www.ons.org/publications-research/voice/news-views/05-2025/oncology-drug-reference-sheet-doxorubicin

Paganelli, M. A., & Popescu, G. K. (2015). Actions of bupivacaine, a widely used local anesthetic, on NMDA receptor responses. The Journal of neuroscience : the official journal of the Society for Neuroscience, 35(2), 831–842. https://doi.org/10.1523/JNEUROSCI.3578-14.2015

Palika, R., Dasi, T., Ghosh, S., Peter, R., Parasannanavar, D. J., Pradhan, A. S., Kurpad, A. V., Sachdev, H. S., Kulkarni, B., & Pullakhandam, R. (2022). Efficacy of iron-folic acid treatment for reducing anemia prevalence and improving iron status in women of reproductive age: A one-year longitudinal study. Clinical nutrition ESPEN, 49, 390–397. https://doi.org/10.1016/j.clnesp.2022.03.016

Pan American Health Organization. (2021). Guideline for Preventive Chemotherapy for the Control of Taenia solium Taeniasis. Pan American Health Organization; 2021. 3, Evidence and Recommendations. Last updated: 2021. Last accessed: November 29 2025. https://www.ncbi.nlm.nih.gov/books/NBK587135/

Papageorghiou, A.T., Kihara, A.B., Dunkley-Bent, J., Ahsan, S., Begum, F., et al. (2025). New guidelines for the prevention, diagnosis, and treatment of postpartum haemorrhage: ending the geography of risk. The Lancet Global Health, Volume 13, Issue 11, e1796-e1798, November 2025. https://doi.org/10.1016/S2214-109X(25)00404-8

Papola, D., Ostuzzi, G., Todesco, B., Gastaldon, C., et al. (2023). Updating the WHO Model Lists of Essential Medicines to promote global access to the most cost-effective and safe medicines for mental disorders. The Lancet Psychiatry. Volume 10, Issue 10, October 2023, Pages 809-816. https://doi.org/10.1016/S2215-0366(23)00176-1

Pappas, P.G., Kauffman, C.A., Andes, D.R., Clancy, C.J., Marr, K.A., et al. (2016). Clinical Practice Guideline for the Management of Candidiasis: 2016 Update by the Infectious Diseases Society of America, Clinical Infectious Diseases, Volume 62, Issue 4, 15 February 2016, Pages e1–e50, https://doi.org/10.1093/cid/civ933

Park, J. H., Shim, J. K., Hong, H., & Lim, H. K. (2020). The efficacy of intramuscular ephedrine in preventing hemodynamic perturbations in patients with spinal anesthesia and dexmedetomidine sedation. International journal of medical sciences, 17(15), 2285–2291. https://doi.org/10.7150/ijms.48772

Park, S.J., Shin, K., Kim, IH. et al. Exploring the efficacy of fluorouracil and platinum based chemotherapy in advanced hepatocellular carcinoma to bridge the treatment gap in resource limited settings. Sci Rep 15, 3386 (2025). https://doi.org/10.1038/s41598-025-86523-9

Parums D. V. (2024). A Review of the Resurgence of Measles, a Vaccine-Preventable Disease, as Current Concerns Contrast with Past Hopes for Measles Elimination. Medical science monitor : international medical journal of experimental and clinical research, 30, e944436. https://doi.org/10.12659/MSM.944436

Pasquini, T. L. S., Rohli, K. E., Almeida, F. J., Banerjee, I., Dastamani, A., De Leon, D. D., Lopez, L. N., Thornton, P. S., & Raskin, J. (2025). Real-world experience with the use of diazoxide among people living with congenital hyperinsulinism and their caregivers. Frontiers in endocrinology, 16, 1628125. https://doi.org/10.3389/fendo.2025.1628125

Patel, P., McLendon, K., Preuss, C.V. (2025). Atropine. StatPearls Publishing, Treasure Island (FL). Last updated: July 6 2025. Last accessed: November 24 2025. https://www.ncbi.nlm.nih.gov/books/NBK470551/

Patocka, J., Wu, Q., Nepvimova, E., Kuca, K. (2020). Phenytoin – An anti-seizure drug: Overview of its chemistry, pharmacology and toxicology. Food and Chemical Toxicology, Volume 142, August 2020, 111393. https://doi.org/10.1016/j.fct.2020.111393

Paul, A. K., Smith, C. M., Rahmatullah, M., Nissapatorn, V., Wilairatana, P., Spetea, M., Gueven, N., & Dietis, N. (2021). Opioid Analgesia and Opioid-Induced Adverse Effects: A Review. Pharmaceuticals, 14(11), 1091. https://doi.org/10.3390/ph14111091

Pearson, R., & Butler, A. (2021). Glyceryl Trinitrate: History, Mystery, and Alcohol Intolerance. Molecules (Basel, Switzerland), 26(21), 6581. https://doi.org/10.3390/molecules26216581

Persaud, N., Jiang, M., Shaikh, R., Bali, A., Oronsaye, E., Woods, H., Drozdzal, G., Rajakulasingam, Y., Maraj, D., Wadhawan, S., Umali, N., Wang, R., McCall, M., Aronson, J. K., Plüddemann, A., Moja, L., Magrini, N., & Heneghan, C. (2019). Comparison of essential medicines lists in 137 countries. Bulletin of the World Health Organization, 97(6), 394–404C. https://doi.org/10.2471/BLT.18.222448

Perumal, R., Naidoo, K., Naidoo, A., Ramachandran, G., Requena-Mendez, A., Sekaggya-Wiltshire, C., Mpagama, S. G., Matteelli, A., Fehr, J., Heysell, S. K., & Padayatchi, N. (2020). A systematic review and meta-analysis of first-line tuberculosis drug concentrations and treatment outcomes. The international journal of tuberculosis and lung disease : the official journal of the International Union against Tuberculosis and Lung Disease, 24(1), 48–64. https://doi.org/10.5588/ijtld.19.0025

Philip, R., Beaney, T., Appelbaum, N. et al. (2021). Variation in hypertension clinical practice guidelines: a global comparison. BMC Med 19, 117 (2021). https://doi.org/10.1186/s12916-021-01963-0

Pickering, M.E., Souberbielle, J.C., Boutten, A., et al. (2025). Daily or intermittent vitamin D supplementation in patients with or at risk of osteoporosis: Position statement from the GRIO. Joint Bone Spine, Volume 92, Issue 3, May 2025, 105858. https://doi.org/10.1016/j.jbspin.2025.105858

Piggott T, Nowak A, Brignardello-Petersen R, et al. (2022). Global status of essential medicine selection: a systematic comparison of national essential medicine lists with recommendations by WHO. BMJ Open 2022;12:e053349. doi: 10.1136/bmjopen-2021-053349

Pinheiro, M., Valente, C., Cruz, M., Nascimento Moreira, D., Aguiar, A., & Duarte, R. (2025). European guidelines for the management of tuberculosis screening procedures in migrants: A systematic review. Pulmonology, 31(1), 2482855. https://doi.org/10.1080/25310429.2025.2482855

Plaud, B., Baillard, C., Bourgain, J.L., Bouroche, G., Desplanque, L., Devys, J.M., Fletcher, D., et al. (2020). Guidelines on muscle relaxants and reversal in anaesthesia. Anaesthesia Critical Care & Pain Medicine. Volume 39, Issue 1, February 2020, Pages 125-142. https://doi.org/10.1016/j.accpm.2020.01.005

Pollard, A.J., Bijker, E.M. (2021). A guide to vaccinology: from basic principles to new developments. Nat Rev Immunol 21, 83–100 (2021). https://doi.org/10.1038/s41577-020-00479-7

Popkin, B. M., D'Anci, K. E., & Rosenberg, I. H. (2010). Water, hydration, and health. Nutrition reviews, 68(8), 439–458. https://doi.org/10.1111/j.1753-4887.2010.00304.x

Portalatin, M., & Winstead, N. (2012). Medical management of constipation. Clinics in colon and rectal surgery, 25(1), 12–19. https://doi.org/10.1055/s-0032-1301754

Post, R. M., & Rybakowski, J. K. (2024). What Patients with Bipolar Disorder Need to Know about Lithium. Pharmaceuticals, 17(9), 1223. https://doi.org/10.3390/ph17091223

Prasad, R., Singh, A., Gupta, N. (2021). Adverse Drug Reactions with First-Line and Second-Line Drugs in Treatment of Tuberculosis. Ann Natl Acad Med Sci 2021; 57(01): 15-35. DOI: 10.1055/s-0040-1722535

Price, R.N., von Seidlein, L., Vaecha, N., Nosten, F., Baird, J.K., White, N.J. (2014). Global extent of chloroquine-resistant Plasmodium vivax: a systematic review and meta-analysis. The Lancet Infectious Diseases, Volume 14, Issue 10, 982 - 991. https://doi.org/10.1016/S1473-3099(14)70855-2

Prince RL, Austin N, Devine A, Dick IM, Bruce D, Zhu K. (2008). Effects of Ergocalciferol Added to Calcium on the Risk of Falls in Elderly High-Risk Women. Arch Intern Med. 2008;168(1):103–108. doi:10.1001/archinternmed.2007.3

Qureshi, H., Massey, E., Kirwan, D., et als. (2014). BCSH guideline for the use of anti-D immunoglobulin for the prevention of haemolytic disease of the fetus and newborn. Transfusion Medicine, Volume 24, Issue 1, February 2014, Pages 8-20. https://doi.org/10.1111/tme.12091

Rabbie, R., Derry, S., & Moore, R. A. (2013). Ibuprofen with or without an antiemetic for acute migraine headaches in adults. The Cochrane database of systematic reviews, 2013(4), CD008039. https://doi.org/10.1002/14651858.CD008039.pub3

Rai, A., Misra, S. R., Panda, S., Sokolowski, G., Mishra, L., Das, R., & Lapinska, B. (2022). Nystatin Effectiveness in Oral Candidiasis Treatment: A Systematic Review & Meta-Analysis of Clinical Trials. Life, 12(11), 1677. https://doi.org/10.3390/life12111677

Ramharter, M., Schlabe, S., Hübner, M.P. et al. (2025). Diagnosis, management and prevention of loiasis: guideline of the German Society for Tropical Medicine, Travel Medicine, and Global Health (DTG). Infection 53, 851–872. https://doi.org/10.1007/s15010-024-02443-2

Ramirez, M. S., & Tolmasky, M. E. (2017). Amikacin: Uses, Resistance, and Prospects for Inhibition. Molecules (Basel, Switzerland), 22(12), 2267. https://doi.org/10.3390/molecules22122267

Randle, R. W., Bates, M. F., Long, K. L., Pitt, S. C., Schneider, D. F., & Sippel, R. S. (2018). Impact of potassium iodide on thyroidectomy for Graves' disease: Implications for safety and operative difficulty. Surgery, 163(1), 68–72. https://doi.org/10.1016/j.surg.2017.03.030

Rao, S., Brenner, D.M. (2021). Efficacy and Safety of Over-the-Counter Therapies for Chronic Constipation: An Updated Systematic Review. The American Journal of Gastroenterology, 116(6):p 1156-1181, June 2021. DOI: 10.14309/ajg.0000000000001222

Rastegar-Pouyani, N., Farzin, M.A., Zafari, J. et al. (2025). Repurposing the anti-parasitic agent pentamidine for cancer therapy; a novel approach with promising anti-tumor properties. J Transl Med 23, 258. https://doi.org/10.1186/s12967-025-06293-w

Rawat, P.S., Jaiswal, A., Khurana, A., eta l. (2021). Doxorubicin-induced cardiotoxicity: An update on the molecular mechanism and novel therapeutic strategies for effective management. Biomedicine & Pharmacotherapy, Volume 139, July 2021, 111708. https://doi.org/10.1016/j.biopha.2021.111708

Reimann, F., & Tomlins, M. (2023). Prescribing and peritoneal dialysis. Australian prescriber, 46(1), 5–8. https://doi.org/10.18773/austprescr.2023.001

Reinhart M, Puil L, Salzwedel DM, Wright JM. (2023). First‐line diuretics versus other classes of antihypertensive drugs for hypertension. Cochrane Database of Systematic Reviews 2023, Issue 7. Art. No.: CD008161. DOI: 10.1002/14651858.CD008161.pub3. Accessed 01 December 2025.

Remijn-Nelissen, L., Verschuuren, J. J. G. M., & Tannemaat, M. R. (2022). The effectiveness and side effects of pyridostigmine in the treatment of myasthenia gravis: a cross-sectional study. Neuromuscular disorders : NMD, 32(10), 790–799. https://doi.org/10.1016/j.nmd.2022.09.002

Rezaei-Tavirani M, Ghafourian S, Sayehmiri F, Pakzad R, Safiri S, et al. (2018). Prevalence of Cotrimoxazole Resistance Uropathogenic Bacteria in Iran: A Systematic Review and Meta-Analysis.Arch Clin Infect Dis.2018;13(5):e63256.https://doi.org/10.5812/archcid.63256.

Rockenbach, M. K., Rohweder, R., Schuler-Faccini, L., Sanseverino, M. T. V., & Kowalski, T. W. (2025). Scientific and Public Health Challenges in Folic Acid Supplementation: Insights from Brazil and Global Implications. Nutrients, 17(17), 2752. https://doi.org/10.3390/nu17172752

Rollins, M.D., Carvalho, B., Vallejo, M., Zakowski, M. (2025). Nitrous Oxide. ASA Committee on Obstetric Anesthesia Working Group. American Society of Anesthesiologists (ASA). Last updated: 2025. Last accessed: December 4 2025. https://www.asahq.org/about-asa/governance-and-committees/asa-committees/committee-on-obstetric-anesthesia/nitrous-oxide#:~:text=Nitrous%20oxide%20is%20one%20of,from%20the%20health%20care%20sector.&text=Inhaled%20N2O%20for,labor%20and%20potential%20adverse%20effects.

Roques, C., Morriss, W. & Haylock-Loor, C. (2022). The other opioid crisis: the situation in low-resource countries. Can J Anesth/J Can Anesth 69, 923–929. https://doi.org/10.1007/s12630-022-02273-6

Rosendorff C. (2011). Why are we still using hydrochlorothiazide?. Journal of clinical hypertension (Greenwich, Conn.), 13(12), 867–869. https://doi.org/10.1111/j.1751-7176.2011.00566.x

Roster, K.I., Grad, Y.H. (2024). Estimating changes in antibiotic consumption in the USA with the introduction of doxycycline post-exposure prophylaxis. The Lancet Microbe, Volume 5, Issue 1, e9. https://doi.org/10.1016/S2666-5247(23)00314-2

Rote Liste. (2022). Bupivacain 0,25% JENAPHARM®. Fachinformation. Rote Liste Service GmbH. Last updated: May 2022. Last accessed: December 3 2025. https://www.fachinfo.de/fi/detail/010955/bupivacain-0-25-jenapharm-r

Rote Liste. (2025b). Cotrim-ratiopharm 400 mg/80 mg Tabletten/ Cotrim forte-ratiopharm 800 mg/160 mg Tabletten. Fachinformation. Rote Liste Service GmbH, Germany. Last updated: September 2025. Last accessed: November 30 2025. https://www.fachinfo.de/fi/detail/023540/cotrim-ratiopharm-400-mg-80-mg-tabletten-cotrim-forte-ratiopharm-800-mg-160-mg-tabletten

Rotundo, L., & Pyrsopoulos, N. (2020). Liver injury induced by paracetamol and challenges associated with intentional and unintentional use. World journal of hepatology, 12(4), 125–136. https://doi.org/10.4254/wjh.v12.i4.125

Rudolph, L. (2024). Steckbrief Amphotericin B. Pharmazeutische Zeitung. Last updated: March 20 2024. Last accessed: November 25 2025. https://www.pharmazeutische-zeitung.de/steckbrief-amphotericin-b-146177/

Ryu, H. Y., Kim, J. Y., Lim, H. K., Yoon, J., Yoo, B. S., Choe, K. H., & Lee, S. H. (2007). Bupivacaine induced cardiac toxicity mimicking an acute non-ST segment elevation myocardial infarction. Yonsei medical journal, 48(2), 331–336. https://doi.org/10.3349/ymj.2007.48.2.331

Sammaritano, L.R., Askanase, A., Bermas, B.L., Dall'Era, M., Duarte-García, A., Hiraki, L.T., Rovin, B.H., Son, M.B.F., Alvarado, A., Aranow, C., Barnado, A., Broder, A., Brunner, H.I., Chowdhary, V., Contreras, G., Felix, C., Ferucci, E.D., Gibson, K.L., Hersh, A.O., Izmirly, P.M., Kalunian, K., Kamen, D., Rollins, B., Smith, B.J., Thomas, A., Timlin, H., Wallace, D.J., Ward, M., Azzam, M., Bartels, C.M., Cunha, J.S., DeQuattro, K., Fava, A., Figueroa-Parra, G., Garg, S., Greco, J., Cuéllar-Gutiérrez, M.C., Iyer, P., Johannemann, A.S., Jorge, A., Kasturi, S., Kawtharany, H., Khawandi, J., Kirou, K.A., Legge, A., Liang, K.V., Lockwood, M.M., Sanchez-Rodriguez, A., Turgunbaev, M., Williams, J.N., Turner, A.S. and Mustafa, R.A. (2025), 2024 American College of Rheumatology (ACR) Guideline for the Screening, Treatment, and Management of Lupus Nephritis. Arthritis Care Res, 77: 1045-1065. https://doi.org/10.1002/acr.25528

Sankar, J., Chauhan, A., Singh, R., Mahajan, D. (2024). Isoniazid-historical development, metabolism associated toxicity and a perspective on its pharmacological improvement. Front. Pharmacol., 19 September 2024, Sec. Drug Metabolism and Transport, Volume 15 - 2024. https://doi.org/10.3389/fphar.2024.1441147

Santiago P. (2012). Ferrous versus ferric oral iron formulations for the treatment of iron deficiency: a clinical overview. TheScientificWorldJournal, 2012, 846824. https://doi.org/10.1100/2012/846824

Sattar, S., Ahmed, T., Rasul, C.H., Saha, D., et al. (2012). Efficacy of a High-Dose in Addition to Daily Low-Dose Vitamin A in Children Suffering from Severe Acute Malnutrition with Other Illnesses. PLOS One, March 2012. https://doi.org/10.1371/journal.pone.0033112

Scholar, E. (2007). Sodium Stibogluconate. xPharm: The Comprehensive Pharmacology Reference, 2007, Pages 1-5. https://doi.org/10.1016/B978-008055232-3.62641-8

Schulman, S., Furie, B. (2015). How I treat poisoning with vitamin K antagonists. Blood, 125 (3): 438–442. https://doi.org/10.1182/blood-2014-08-597781

Schwenk, E.S., Sneyd, J.R., Wu, C.L. (2025). The state of local anaesthetic systemic toxicity in 2025: the emergence of lidocaine as our next challenge. BJA, Volume 135, Issue 4, p854-856, October 2025. https://doi.org/10.1016/j.bja.2025.07.042

Sebode, M., Reike-Kunze, M., Weidemann, S., Zenouzi, R., Hartl, J., Peiseler, M., Liwinski, T., Schulz, L., Weiler-Normann, C., Sterneck, M., Lohse, A. W., & Schramm, C. (2020). Metamizole: An underrated agent causing severe idiosyncratic drug-induced liver injury. British journal of clinical pharmacology, 86(7), 1406–1415. https://doi.org/10.1111/bcp.14254

Seghers, F., Taylor, M. M., Storey, A., Dong, J., Wi, T. C., Wyber, R., Ralston, K., & Nguimfack, B. D. (2024). Securing the supply of benzathine penicillin: a global perspective on risks and mitigation strategies to prevent future shortages. International health, 16(3), 279–282. https://doi.org/10.1093/inthealth/ihad087

Shafrin, J., Quddus, S., Marin, A., Scanlon, D. (2023). A Decade of Health Innovation: The Impact of New Medicines on Patient Health and the Implications for NICE’s Size of Benefit Multiplier. Value in Health. Volume 26, Issue 10, October 2023, Pages 1435-1439. https://doi.org/10.1016/j.jval.2023.06.009

Shaheed, C.A., Maher, C.G., McLachlan, A. (2016). Investigating the efficacy and safety of over-the-counter codeine containing combination analgesics for pain and codeine based antitussives. Therapeutic Goods Administration, Australia. Last updated: March 21 2016. Last accessed: December 7 2025. https://www.tga.gov.au/sites/default/files/review-efficacy-and-safety-over-counter-codeine-combination-medicines.pdf

Shamim, A., Afzal, K. & Manazir Ali, S. (2014).Safety and efficacy of isotonic (0.9%) vs. hypotonic (0.18%) saline as maintenance intravenous fluids in children: A randomized controlled trial . Indian Pediatr 51, 969–974 (2014). https://doi.org/10.1007/s13312-014-0542-5

Sharma, B., Sharma, A.K. & Sharma, U. (2022) Sporotrichosis: a Comprehensive Review on Recent Drug-Based Therapeutics and Management. Curr Derm Rep 11, 110–119 (2022). https://doi.org/10.1007/s13671-022-00358-5

Sharma, S., Shamy, O. E., Wilmington, A., Keller, B., Aslam, N., Tran, H., Rutherford, P., & Uribarri, J. (2024). Performance Evaluation of an Automated Peritoneal Dialysis Solution Generation System in Patients Using Automated Peritoneal Dialysis. Kidney international reports, 9(6), 1752–1757. https://doi.org/10.1016/j.ekir.2024.03.010

Shawkat, H., Westwood, M.M., Mortimer, A. (2012). Mannitol: a review of its clinical uses. BJA, Volume 12, Issue 2, P82-85, April 2012. https://doi.org/10.1093/bjaceaccp/mkr063

Shea BJ, Adachi JD, Cranney A, Griffith L, Guyatt G, Hamel C, Ortiz Z, Peterson J, Robinson VA, Tugwell P, Wells G, Zytaruk N. (2004). Calcium supplementation on bone loss in postmenopausal women. Cochrane Database of Systematic Reviews 2004, Issue 1. Art. No.: CD004526. DOI: 10.1002/14651858.CD004526.pub2.

Shields, R. C., McBane, R. D., Kuiper, J. D., Li, H., & Heit, J. A. (2001). Efficacy and safety of intravenous phytonadione (vitamin K1) in patients on long-term oral anticoagulant therapy. Mayo Clinic proceedings, 76(3), 260–266. https://doi.org/10.4065/76.3.260

Shipman, S., Painter, K., Keuchel, M., Bogie, C. (2021). Short-Term Topical Tetracaine Is Highly Efficacious for the Treatment of Pain Caused by Corneal Abrasions: A Double-Blind, Randomized Clinical Trial. Annals of Emergency Medicine, Volume 77, Issue 3, March 2021, Pages 338-344. https://doi.org/10.1016/j.annemergmed.2020.08.036

Sigma-Aldrich. (2025). Water Properties, Polarity and Role as a Universal Solvent. Sigma-Aldrich, Merck KgaA, Darmstadt. Last updated: 2025. Last accessed: November 24 2025. https://www.sigmaaldrich.com/DE/de/technical-documents/technical-article/water-purification/understanding-lab-water/water-properties-polarity-role-as-solvent

Silber, S. A., Diro, E., Workneh, N., Mekonnen, Z., Levecke, B., Steinmann, P., Umulisa, I., Alemu, H., Baeten, B., Engelen, M., Hu, P., Friedman, A., Baseman, A., & Mrus, J. (2017). Efficacy and Safety of a Single-Dose Mebendazole 500 mg Chewable, Rapidly-Disintegrating Tablet for Ascaris lumbricoides and Trichuris trichiura Infection Treatment in Pediatric Patients: A Double-Blind, Randomized, Placebo-Controlled, Phase 3 Study. The American journal of tropical medicine and hygiene, 97(6), 1851–1856. https://doi.org/10.4269/ajtmh.17-0108

Silva, H., Liyanage, A., Deerasinghe, T., Chandrasekara, V., Chellappan, K., & Karunaweera, N. D. (2021). Treatment failure to sodium stibogluconate in cutaneous leishmaniasis: A challenge to infection control and disease elimination. PloS one, 16(10), e0259009. https://doi.org/10.1371/journal.pone.0259009

Silva, J.P., Nogueira, B.V., Mussolini, M.C., eta l. (2026). Full Heparin Reversal With Protamine After Transcatheter Aortic Valve Replacement: A Systematic Review and Meta-Analysis. The American Journal of Cardiology, Volume 258, 1 January 2026, Pages 239-246. https://doi.org/10.1016/j.amjcard.2025.09.057

Sirko, J., Shi, W. (2024). Disruptive innovation events in dentistry. The Journal of the American Dental Association. Volume 155, Issue 10, October 2024, Pages 899-901. https://doi.org/10.1016/j.adaj.2024.07.013

Skarpeid, P. L., & Høye, S. (2018). Phenoxymethylpenicillin Versus Amoxicillin for Infections in Ambulatory Care: A Systematic Review. Antibiotics (Basel, Switzerland), 7(3), 81. https://doi.org/10.3390/antibiotics7030081

Škubník, J., Pavlíčková, V. S., Ruml, T., & Rimpelová, S. (2021). Vincristine in Combination Therapy of Cancer: Emerging Trends in Clinics. Biology, 10(9), 849. https://doi.org/10.3390/biology10090849

Slifka MK, Thomas A, Gao L, Amanna IJ, Orenstein WA. (2025).Lessons learned from successful implementation of tetanus and diphtheria vaccination programs. Clin Microbiol Rev 38:e00031-25.https://doi.org/10.1128/cmr.00031-25

Sodré-Alves, B. M. C., Toledo, M. M., Zimmermann, I. R., Araújo, W. N., & Tavares, N. U. L. (2024). Isoniazid use, effectiveness, and safety for treatment of latent tuberculosis infection: a systematic review. Revista da Sociedade Brasileira de Medicina Tropical, 57, e004022024. https://doi.org/10.1590/0037-8682-0504-2023

Sohn, S.Y., Inoue, K., Rhee, C.M., et al. (2024). Risks of Iodine Excess. Endocrine Reviews, Volume 45, Issue 6, December 2024, Pages 858–879, https://doi.org/10.1210/endrev/bnae019

Solomon, M., Ollech, A., Pavlotsky, F., Barzilai, A., Schwartz, E., Baum, S., Astman, N. (2024). Comparison of Intralesional Sodium Stibogluconate versus Intralesional Meglumine Antimoniate for the Treatment of Leishmania major Cutaneous Leishmaniasis. Acta Dermato-Venereologica, 28 Apr 2024. http://dx.doi.org/10.2340/actadv.v104.35089

Soman, B., Vijayaraghavan, G. (2017). The role of organic nitrates in the optimal medical management of angina. E-Journal of Cardiology Practive, Vol. 15, No. 2. Last updated: April 26 2017. Last accessed: December 2 2025. https://www.escardio.org/Journals/E-Journal-of-Cardiology-Practice/Volume-15/The-role-of-organic-nitrates-in-the-optimal-medical-management-of-angina

Sosa LE, Njie GJ, Lobato MN, et al. (2019). Tuberculosis Screening, Testing, and Treatment of U.S. Health Care Personnel: Recommendations from the National Tuberculosis Controllers Association and CDC, 2019. MMWR Morb Mortal Wkly Rep 2019;68:439–443. DOI: http://dx.doi.org/10.15585/mmwr.mm6819a3.

Sousa, F., Nascimentom C., Ferreira, D., Reis, S., Costa, P. (2023). Reviving the interest in the versatile drug nystatin: A multitude of strategies to increase its potential as an effective and safe antifungal agent. Advanced Drug Delivery Reviews, Volume 199, August 2023, 114969. https://doi.org/10.1016/j.addr.2023.114969

Sousou, J. M., Griffith, E. M., Marsalisi, C., & Reddy, P. (2023). Pyridoxine Deficiency and Neurologic Dysfunction: An Unlikely Association. Cureus, 15(10), e47647. https://doi.org/10.7759/cureus.47647

Spallek, L., Krille, L., Reiners, C., Schneider, R., Yamashita, S., & Zeeb, H. (2012). Adverse effects of iodine thyroid blocking: a systematic review. Radiation protection dosimetry, 150(3), 267–277. https://doi.org/10.1093/rpd/ncr400

Sparo, M., Delpech, G., Allende, N.G. (2018). Impact on Public Health of the Spread of High-Level Resistance to Gentamicin and Vancomycin in Enterococci. Front. Microbiol., 18 December 2018. Sec. Antimicrobials, Resistance and Chemotherapy, Volume 9 - 2018. https://doi.org/10.3389/fmicb.2018.03073

Spencer, S. P. E., & Lowe, S. A. (2019). Ergometrine for postpartum hemorrhage and associated myocardial ischemia: Two case reports and a review of the literature. Clinical case reports, 7(12), 2433–2442. https://doi.org/10.1002/ccr3.2516

Sprenger, T., Viana, M., Tassorelli, C. (2018). Current Prophylactic Medications for Migraine and Their Potential Mechanisms of Action. Neurotherapeutics, Volume 15, Issue 2, April 2018, Pages 313-323. https://doi.org/10.1007/s13311-018-0621-8

Sritharan, S., Sivalingam, N. (2021). A comprehensive review on time-tested anticancer drug doxorubicin. Life Sciences, Volume 278, 1 August 2021, 119527. https://doi.org/10.1016/j.lfs.2021.119527

Stadler, J. A. M., Maartens, G., Meintjes, G., & Wasserman, S. (2023). Clofazimine for the treatment of tuberculosis. Frontiers in pharmacology, 14, 1100488. https://doi.org/10.3389/fphar.2023.1100488

Stanley, S.L. (2010). Antiparasitic agents. Infectious Diseases. Mosby. 3rd edition, Volume 2, 2010, Chapter 150, Pages 1490-1507. https://doi.org/10.1016/B978-0-323-04579-7.00150-7

Starshinova A, Kudryavtsev I, Rubinstein A, Dovgalyuk I, Kulpina A, Churilov LP. and Kudlay D (2025) BCG vaccination: historical role, modern applications, and future perspectives in tuberculosis and beyond. Front. Pediatr. 13:1603732. doi: 10.3389/fped.2025.1603732

Stavleu DC, Mulder RL, Kruimer DM Procedural Local Anesthetics Guideline Panel, et al. (2024). Topical analgesia during needle-related procedures in children: a clinical practice guideline. Archives of Disease in Childhood 2025;110:657-661. https://adc.bmj.com/content/110/8/657

Sudhakar, P., Acharya, K., Kini, T.A. (2025). Ethambutol optic neuropathy. Front. Neurol., 11 September 2025, Sec. Neuro-Ophthalmology, Volume 16 - 2025. https://doi.org/10.3389/fneur.2025.1626909

Sykes, D.A., Moore, H., Stott, L. et al. (2017). Extrapyramidal side effects of antipsychotics are linked to their association kinetics at dopamine D2 receptors. Nat Commun 8, 763 (2017). https://doi.org/10.1038/s41467-017-00716-z

Taglione MS, Persaud N. (2021). Assessing variation among the national essential medicines lists of 21 high-income countries: a cross-sectional study. BMJ Open 2021;11:e045262. doi: 10.1136/bmjopen-2020-045262

Tamargo, J., Agewall, S., Ambrosio, G., Borghi, C., Cerbai, E., Dan, G.A., et al. (2025). New pharmacological agents and novel cardiovascular pharmacotherapy strategies in 2024. European Heart Journal - Cardiovascular Pharmacotherapy, Volume 11, Issue 3, May 2025, Pages 292–317, https://doi.org/10.1093/ehjcvp/pvaf012

Tay, E. M. Y., & Roberts, D. M. (2018). A spotlight on the role, use, and availability of codeine and the implications faced. Expert Review of Clinical Pharmacology, 11(11), 1057–1059. https://doi.org/10.1080/17512433.2018.1537122

The Royal Children's Hospital Melbourne (RCH). (2020). Hypernatraemia. Clinical Practice Guidelines. Last updated: October 2020. Last accessed: November 20 2025. https://www.rch.org.au/clinicalguide/guideline_index/hypernatraemia/

Therapeutic Goods Administration. (2016). Update of codeine safety and efficacy review. Version 1.0. Australian Government, Department of Health. Last updated: December 2016. Last accessed: December 7 2025. https://www.tga.gov.au/sites/default/files/update-codeine-safety-and-efficacy-review.pdf

Thomas, D., Seetharam, G., & Alvarez-Uria, G. (2012). Essential medicines concept for quality assurance of health care facilities. Journal of pharmacy & bioallied sciences, 4(2), 172. https://doi.org/10.4103/0975-7406.94826

Thomas, P.G., Garg, N. (2007). Chapter 60 - Amebiasis and Other Parasitic Infections. Surgery of the Liver, Biliary Tract and Pancreas (Fourth Edition). VOLUME 2, 2007, Pages 935-951. https://doi.org/10.1016/B978-1-4160-3256-4.50072-7

Thour, A., Marwaha, R. (2023). Amitriptyline. StatPearls Publishing, Treasure Island (FL). Last updated: July 18 2023. Last accessed: December 3 2025. https://www.ncbi.nlm.nih.gov/books/NBK537225/

Tobaiqi, M. A., Alshamrani, M. N., Sriram, S., Mahmoud, A. B., Fadlalmola, H. A., & Albadrani, M. (2025). Interferon-Gamma Release Assays Versus Tuberculin Skin Test for Active Tuberculosis Diagnosis: A Systematic Review and Diagnostic Meta-Analysis. Diagnostics (Basel, Switzerland), 15(18), 2343. https://doi.org/10.3390/diagnostics15182343

Tolkien, Z., Stecher, L., Mander, A. P., Pereira, D. I., & Powell, J. J. (2015). Ferrous sulfate supplementation causes significant gastrointestinal side-effects in adults: a systematic review and meta-analysis. PloS one, 10(2), e0117383. https://doi.org/10.1371/journal.pone.0117383

Trangkanont, T., Puwanant, M., & Chotsampancharoen, T. (2025). Clinical Characteristics and Outcomes of Pediatric Vitamin C Deficiency. Nutrients, 17(23), 3755. https://doi.org/10.3390/nu17233755

Trinka, E. (2023). Phenobarbital in Status epilepticus – Rediscovery of an effective drug. Epilepsy & Behavior, Volume 141, April 2023, 109104. https://doi.org/10.1016/j.yebeh.2023.109104

Truelove, S. A., Keegan, L. T., Moss, W. J., Chaisson, L. H., Macher, E., Azman, A. S., & Lessler, J. (2020). Clinical and Epidemiological Aspects of Diphtheria: A Systematic Review and Pooled Analysis. Clinical infectious diseases : an official publication of the Infectious Diseases Society of America, 71(1), 89–97. https://doi.org/10.1093/cid/ciz808

Tsakatikas, S., Papageorgiou, G., Fioretzaki, R., Kosmas, C. (2021). An overview of current results with the vincristine-irinotecan-temozolomide combination with or without bevacizumab in pediatric, adolescence and adult solid tumors. Critical Reviews in Oncology/Hematology, Volume 166, October 2021, 103457. https://doi.org/10.1016/j.critrevonc.2021.103457

U.S. Centers for Diasease Control and Prevention (CDC). (2021). Primary and Secondary Syphilis. Sexually Transmitted Infections Treatment Guidelines, 2021. Last updated: July 22 2021. Last accessed: November 27 2025. https://www.cdc.gov/std/treatment-guidelines/p-and-s-syphilis.htm

U.S. Centers for Diasease Control and Prevention (CDC). (2024a). Clinical Treatment of Lymphatic Filariasis. Last updated: June 13 2024. Last accessed: November 28 2025.https://www.cdc.gov/filarial-worms/hcp/clinical-care/index.html

U.S. Centers for Disease Control and Prevention (CDC). (2024c). Clinical Care of Toxoplasmosis. Last updated: January 22 2024. Last accessed: November 29 2025. https://www.cdc.gov/std/treatment-guidelines/syphilis.htm

U.S. Centers for Disease Control and Prevention (CDC). (2025). Bacille Calmette-Guérin (BCG) Vaccine for Tuberculosis. Tuberculosis (TB). Last updated: January 31 2025. Last accessed: December 6 2025. https://www.cdc.gov/tb/hcp/vaccines/index.html

U.S. Centers for Disease Control and Prevention (CDC). (2025). Fast Facts: Global Tetanus Vaccination. Last updated: November 17 2025. Last accessed: December 6 2025. https://www.cdc.gov/global-tetanus-vaccination/data-research/index.html

U.S. Centers for Disease Control and Prevention (CDC). (2025). Rabies Post-exposure Prophylaxis Guidance. Rabies. Last updated: July 15 2025. Last accessed: December 6 2025. https://www.cdc.gov/rabies/hcp/clinical-care/post-exposure-prophylaxis.html

UK Health Security Agency. (2025). Public health control and management of diphtheria in England. 2025 guidelines. Publishing reference: GOV-19100. Last updated: July 2025. Last accessed: December 6 2025. https://assets.publishing.service.gov.uk/media/68778311f5eb08157f3637d8/National_diphtheria_guidance_July2025.pdf

UL Haq, U., Majeed, N., Kumari, N., et al. (2025). Phenobarbital versus valproate for generalized convulsive status epilepticus in adults. An updated systematic review and meta-analysis. Epilepsy Research, Volume 215, September 2025, 107571. https://doi.org/10.1016/j.eplepsyres.2025.107571

United Nations Children's Fund (UNICEF). (2025). Oral rehydration salts and zinc. UNICEF, Supply Division. Last updated: 2025. Last accessed: December 6 2025. https://www.unicef.org/supply/oral-rehydration-salts-and-zinc

Universitätsklinikum Ulm (UKU). (2020). Leistungsverzeichnis Amikacin FB-PÄ 6 AMIK OE. ID 21297. Last updated: June 05 2020. Last accessed: November 24 2025. https://www.uniklinik-ulm.de/fileadmin/default/09_Sonstige/Klinische-Chemie/Seiteninhalte/Seiteninhalte_A/Amikacin_FB-PAE_6_AMIK_OE.pdf

van der Walt ML, Shean K, Becker PKeddy KH, Lancaster J. (2020). Treatment Outcomes and Adverse Drug Effects of Ethambutol, Cycloserine, and Terizidone for the Treatment of Multidrug-Resistant Tuberculosis in South Africa. Antimicrob Agents Chemother 65:10.1128/aac.00744-20. https://doi.org/10.1128/aac.00744-20

van Eijk, A.M., Stepniewska, K., Khairallah, C., et al. (2025). The impact of sulfadoxine–pyrimethamine resistance on the effectiveness of intermittent preventive treatment for the prevention of malaria in pregnancy in Africa: an updated systematic review and meta-analysis. The Lancet Infectious Diseases. Volume 25, Issue 12, p1336-1346, December 2025. https://doi.org/10.1016/S1473-3099(25)00219-1

van Henten, S., Bialfew, F., Hassen, S., Tilahun, F., van Griensven, J., & Abdela, S. G. (2023). Treatment of Cutaneous Leishmaniasis with Sodium Stibogluconate and Allopurinol in a Routine Setting in Ethiopia: Clinical and Patient-Reported Outcomes and Operational Challenges. Tropical medicine and infectious disease, 8(8), 414. https://doi.org/10.3390/tropicalmed8080414

Van Hout, M.C., Norman, I. (2016). Misuse of non-prescription codeine containing products: Recommendations for detection and reduction of risk in community pharmacies. International Journal of Drug Policy, Volume 27, January 2016, Pages 17-22. https://doi.org/10.1016/j.drugpo.2015.09.007

van Ingen, J. (2024). Why do we use 100 mg of clofazimine in TB and NTM treatment? Journal of Antimicrobial Chemotherapy, Volume 79, Issue 4, April 2024, Pages 697–702. https://doi.org/10.1093/jac/dkae041

Van Netten, C., Vallabhaneni, K., Hardwick, B., Anumba, D., Briley, A. L., Collins, P., Collis, R. E., Deja, E., Gkioni, E., Gyte, G., Hickey, H., Hinshaw, K., Hughes, D. A., Kenyon, S., Lavender, T., Meher, S., Plumpton, C., Robson, S., Rosala-Hallas, A., Saviciute, E., … Weeks, A. (2025). Carboprost versus Oxytocin as the first-line treatment of primary postpartum haemorrhage (COPE): protocol for a phase IV, double-blind, double-dummy, randomised controlled trial and economic analysis. BMJ open, 15(5), e101255. https://doi.org/10.1136/bmjopen-2025-101255

Velioglu, O., Calis, A.S., Koca, H. et al. (2020). Bupivacaine vs. lidocaine: a comparison of local anesthetic efficacy in impacted third molar surgery. Clin Oral Invest 24, 3539–3546 (2020). https://doi.org/10.1007/s00784-020-03224-5

Vos, C. F., Aarnoutse, R. E., Op de Coul, M. J. M., Spijker, J., Groothedde-Kuyvenhoven, M. M., Mihaescu, R., Wessels-Basten, S. J. W., Rovers, J. J. E., Ter Hark, S. E., Schene, A. H., Hulscher, M. E. J. L., & Janzing, J. G. E. (2021). Tricyclic antidepressants for major depressive disorder: a comprehensive evaluation of current practice in the Netherlands. BMC psychiatry, 21(1), 481. https://doi.org/10.1186/s12888-021-03490-x

Wang, W.Z., Wu, J.Z., Ma, G.Y., et al. (2006). Efficacy assessment of phenobarbital in epilepsy: a large community-based intervention trial in rural China. The Lancet Neurology, Volume 5, Issue 1, p46-52, January 2006. https://doi.org/10.1016/S1474-4422(05)70254-4

Wang, X., Arima, H., Yang, J., et al. (2015). Mannitol and Outcome in Intracerebral Hemorrhage: Propensity Score and Multivariable Intensive Blood Pressure Reduction in Acute Cerebral Hemorrhage Trial 2 Results. Stroke, Volume 46, Number 10. https://doi.org/10.1161/STROKEAHA.115.009357

Watson R. (2014). A Review of Phenytoin Monotherapy in Adults With Nonrefractory Status Epilepticus. Journal of Pharmacy Technology. 2014;31(3):99-103. doi:10.1177/8755122514554457

Watson, J., Lee, M. & Garcia-Casal, M.N. Consequences of Inadequate Intakes of Vitamin A, Vitamin B12, Vitamin D, Calcium, Iron, and Folate in Older Persons. Curr Geri Rep 7, 103–113 (2018). https://doi.org/10.1007/s13670-018-0241-5

Weiser, P., Addis, V.M., Fraunfelder, F.W., Jacobs, D.S., et al. (2024). Topical Anesthetics: The Latest on Use for Corneal Abrasions. EyeNet Magazine, Clinical Update, American Academy of Opthalmology. Last updtaed: February 1 2024. Last accessed: December 6 2025. https://www.aao.org/Assets/b5c8a75a-c54e-494a-b4d0-a41d54ba648b/638421570127530000/0224-february-clinical-update-cornea-pdf

Weissel, M. (2010). Propylthiouracil: Clinical Overview of its Efficacy and its Side Effects more than 50 Years after the Introduction of its Use in Thyrostatic Treatment. Exp Clin Endocrinol Diabetes 2010; 118(2): 101-104. DOI: 10.1055/s-0029-1215587

Whorwell, P., Lange, R., & Scarpignato, C. (2024). Review article: do stimulant laxatives damage the gut? A critical analysis of current knowledge. Therapeutic advances in gastroenterology, 17, 17562848241249664. https://doi.org/10.1177/17562848241249664

Wigenstam, E., Artursson, E., Bucht, A., Thors, L. (2022). Supplemental treatment to atropine improves the efficacy to reverse nerve agent induced bronchoconstriction. Chemico-Biological Interactions. Volume 364, 1 September 2022, 110061. https://doi.org/10.1016/j.cbi.2022.110061

Wirth, J. P., Petry, N., Tanumihardjo, S. A., Rogers, L. M., McLean, E., Greig, A., Garrett, G. S., Klemm, R. D. W., & Rohner, F. (2017). Vitamin A Supplementation Programs and Country-Level Evidence of Vitamin A Deficiency. Nutrients, 9(3), 190. https://doi.org/10.3390/nu9030190

World Health Organisation (WHO). (2025d). Global antibiotic resistance surveillance report 2025. WHO Global Antimicrobial Resistance and Use Surveillance System (GLASS). ISBN 978-92-4-011633-7. Last updated: 2025. Last accessed: November 30 2025. https://media.tghn.org/medialibrary/2025/10/Global_antibiotic_esistance_Surveillance_report_2025.pdf

World Health Organization (WHO). (1979). The selection of essential drugs. Report of a WHO Expert Committee. WHO Technical Report Series 641. WHO, Geneva, 1979. ISBN 92 4 120641.

World Health Organization (WHO). (1983). The selection of essential drugs. Report of a WHO Expert Committee. WHO Technical Report Series 685. WHO, Geneva, 1983. ISBN 92 4 120685 3.

World Health Organization (WHO). (1985). The selection of essential drugs. Second report of the WHO Expert Committee. WHO Technical Report Series 722. WHO, Geneva, 1985. ISBN 92 4 120722 1.

World Health Organization (WHO). (1988). The selection of essential drugs. Third report of the WHO Expert Committee. WHO Technical Report Series 770. WHO, Geneva, 1988. ISBN 92 4 120770 1.

World Health Organization (WHO). (1990). The selection of essential drugs. Fourth report of the WHO Expert Committee. WHO Technical Report Series 796. WHO, Geneva, 1990. ISBN 92 4 120796 5.

World Health Organization (WHO). (1992). The selection of essential drugs. Fifth report of the WHO Expert Committee. WHO Technical Report Series 825. WHO, Geneva, 1992. ISBN 9241208252.

World Health Organization (WHO). (1995). The selection of essential drugs. Sixth report of the WHO Expert Committee. WHO Technical Report Series 850. WHO, Geneva, 1995. ISBN 92 4 120850 3.

World Health Organization (WHO). (1997). The selection of essential drugs. Seventh report of the WHO Expert Committee. WHO Technical Report Series 867. WHO, Geneva, 1997. ISBN 92 4 120867 8.

World Health Organization (WHO). (1998). The selection of essential drugs. Eight report of the WHO Expert Committee. WHO Technical Report Series 882. WHO, Geneva, 1998. ISBN 92 4 120882 1.

World Health Organization (WHO). (2000). The selection of essential drugs. Ninth report of the WHO Expert Committee. WHO Technical Report Series 895. WHO, Geneva, 2000. ISBN 92 4 120895 3.

World Health Organization (WHO). (2003). The selection and use of essential medicines. Report of the WHO Expert Committee, 2002. WHO Technical Report Series 914. WHO, Geneva, 2003. ISBN 92 4 120914 3.

World Health Organization (WHO). (2004). The selection and use of essential medicines. Report of the WHO Expert Committee, 2003. WHO Technical Report Series 920. WHO, Geneva, 2004. ISBN 92 4 120920 8.

World Health Organization (WHO). (2006a). The selection and use of essential medicines. Report of the WHO Expert Committee, 2005. WHO Technical Report Series 933. WHO, Geneva, 2006. ISBN 92 4 120933 X.

World Health Organization (WHO). (2007). The selection and use of essential medicines. Report of the WHO Expert Committee, 2007. WHO Technical Report Series 946. WHO, Geneva, 2007. ISBN 978 92 4 120946 5.

World Health Organization (WHO). (2009). The selection and use of essential medicines. Report of the WHO Expert Committee, 2009. WHO Technical Report Series 958. WHO, Geneva, 2009. ISBN 978 92 4 120958 8.

World Health Organization (WHO). (2012). The selection and use of essential medicines. Report of the WHO Expert Committee, 2011. WHO Technical Report Series 965. WHO, Geneva, 2012. ISBN 978 92 4 120965 6.

World Health Organization (WHO). (2014). The selection and use of essential medicines. Report of the WHO Expert Committee, 2013. WHO Technical Report Series 965. WHO, Geneva, 2013. ISBN 978 92 4 120985 4.

World Health Organization (WHO). (2015). The selection and use of essential medicines. Report of the WHO Expert Committee, 2015. WHO Technical Report Series 994. WHO, Geneva, 2015. ISBN 978 92 4 069494 1.

World Health Organization (WHO). (2017). The selection and use of essential medicines. Report of the WHO Expert Committee, 2017. WHO Technical Report Series 1006. WHO, Geneva, 2017. ISBN 978-92-4-121015-7.

World Health Organization (WHO). (2018b). Rabies vaccines: WHO position paper. Weekly epidemiological record, No 16, 2018, 93, 201–220. Last updated: April 2018. Last accessed: December 6 2025. https://www.who.int/publications/i/item/who-wer9316

World Health Organization (WHO). (2019a). The selection and use of essential medicines. Report of the WHO Expert Committee, 2019. WHO Technical Report Series 1021. WHO, Geneva, 2019. ISBN 978-92-4-121030-0.

World Health Organization (WHO). (2019b). Protecting all against tetanus. Guide to sustaining maternal and neonatal tetanus elimination (MNTE) and broadening tetanus protection for all populations. ISBN 978-92-4-151561-0. Last updated: 2019. Last accessed: December 6 2025. https://www.who.int/docs/default-source/tetanus/9789241515610-eng.pdf?sfvrsn=8b4e845a_2

World Health Organization (WHO). (2021). The selection and use of essential medicines. Report of the WHO Expert Committee, 2021. WHO Technical Report Series 1035. WHO, Geneva, 2021. ISBN 978-92-4-004113-4.

World Health Organization (WHO). (2022). Updated WHO recommendations for malaria chemoprevention among children and pregnant women. Last updated: June 3 2022. Last accessed: November 29 2025. https://www.who.int/news/item/03-06-2022-Updated-WHO-recommendations-for-malaria-chemoprevention-among-children-and-pregnant-women

World Health Organization (WHO). (2024a). The selection and use of essential medicines. Report of the WHO Expert Committee, 2023. WHO Technical Report Series 1049. WHO, Geneva, 2024. ISBN 978-92-4-008926-6.

World Health Organization (WHO). (2024b). WHO operational handbook on tuberculosis. Module 1: prevention - tuberculosis preventive treatment, second edition. Geneva: World Health Organization; 2024. ISBN 978-92-4-009777-3. Last updated: 2024. Last accessed: December 7 2025. https://tbksp.who.int/en/node/752

World Health Organization (WHO). (2025e). WHO updates guidelines on opioid dependence treatment and overdose prevention. Last updated: February 9 2025. Last accessed: December 4 2025. https://www.who.int/news/item/09-02-2025-who-updates-guidelines-on-opioid-dependence-treatment-and-overdose-prevention

Xie, X., Fu, Q., Bao, Z., Zhang, Y., Zhou, D. (2020). Clinical value of different anti-D immunoglobulin strategies for preventing Rh hemolytic disease of the fetus and newborn: A network meta-analysis. PLOS One, March 12 2020. https://doi.org/10.1371/journal.pone.0230073

Xue, S., Gu, R., Wu, T., Zhang, M., & Wang, X. (2009). Oral potassium iodide for the treatment of sporotrichosis. The Cochrane database of systematic reviews, 2009(4), CD006136. https://doi.org/10.1002/14651858.CD006136.pub2

Xue, SL., Li, L. (2008). Oral potassium iodide for the treatment of sporotrichosis. Mycopathologia 167, 355–356 (2009). https://doi.org/10.1007/s11046-008-9178

Yilma, D., Stepniewska, K., Bousema, T., Drakeley, C., et al. (2025). Safety and efficacy of single-dose primaquine to interrupt Plasmodium falciparum malaria transmission in children compared with adults: a systematic review and individual patient data meta-analysis. The Lancet Infectious Diseases. Volume 25, Issue 9, p965-976, September 2025. https://doi.org/10.1016/S1473-3099(25)00078-7

Zaccarini, D.J., Lubin, D., Sanyal, S. et al. (2022). Barium Sulfate Deposition in the Gastrointestinal Tract: Review of the literature. Diagn Pathol 17, 99 (2022). https://doi.org/10.1186/s13000-022-01283-8

Zafar, A., Khatoon, S., Khan, M. J., Abu, J., & Naeem, A. (2025). Advancements and limitations in traditional anti-cancer therapies: a comprehensive review of surgery, chemotherapy, radiation therapy, and hormonal therapy. Discover oncology, 16(1), 607. https://doi.org/10.1007/s12672-025-02198-8

Zaloum, S.A., Mair, D., Mchir, A.P., Smith, L.J., et al. (2025). Tackling the growing burden of nitrous oxide-induced public health harms. The Lancet Public Health, Volume 10, Issue 3, e257-e263, March 2025. https://doi.org/10.1016/S2468-2667(24)00298-6

Zanichelli, V., Sharland, M., Cappello, B., Moja, L., Getahun, H., Pessoa-Silva, C., Sati, H., van Weezenbeek, C., Balkhy, H., Simão, M., Gandra, S., & Huttner, B. (2023). The WHO AWaRe (Access, Watch, Reserve) antibiotic book and prevention of antimicrobial resistance [Livre sur les antibiotiques AWaRe (Access, Watch, Reserve) de l'OMS et prévention de la résistance aux antimicrobiens]. Bulletin of the World Health Organization, 101(4), 290–296. https://doi.org/10.2471/BLT.22.288614

Zarzeczny, A., Bradley, C., Ge, L. (2025). How are surgical and clinical innovations in medical practice defined for the purpose of determining regulatory jurisdiction and oversight responsibility?: A scoping review. Ethics, Medicine and Public Health. Volume 33, 2025, 101044. https://doi.org/10.1016/j.jemep.2025.101044

Zhang, L. L., Zeng, L. N., & Li, Y. P. (2011). Side effects of phenobarbital in epilepsy: a systematic review. Epileptic disorders : international epilepsy journal with videotape, 13(4), 349–365. https://doi.org/10.1684/epd.2011.0444

Zhou, M., Liu, A.M., Yang, X.B., Guan, C.P. et al. (2024). The efficacy and safety of high-dose isoniazid-containing therapy for multidrug-resistant tuberculosis: a systematic review and meta-analysis. Front. Pharmacol., 08 January 2024, Sec. Pharmacology of Infectious Diseases, Volume 14 - 2023. https://doi.org/10.3389/fphar.2023.1331371

Zimmermann, M. B., Connolly, K., Bozo, M., Bridson, J., Rohner, F., & Grimci, L. (2006). Iodine supplementation improves cognition in iodine-deficient schoolchildren in Albania: a randomized, controlled, double-blind study. The American journal of clinical nutrition, 83(1), 108–114. https://doi.org/10.1093/ajcn/83.1.108
